# Delayed astrocyte development impairs *Sema6a*-*Plxna2/4*-mediated astrocyte-neuron crosstalk and causes depressive-like behavior

**DOI:** 10.1038/s41467-026-75697-z

**Published:** 2026-07-18

**Authors:** Xin Jiang, Yanqing Qi, Lin Yang, Feihong Yang, Rongliang Guo, Liang Li, Kun Wang, Lichen Sun, Dan Dai, Hanchen Liu, Yanjing Gao, Mengge Sun, Xiaolei Song, Zhuangzhi Zhang, Zhejun Xu, Bin Luo, Yunli Xie, Zhengang Yang, Miao He, Dashi Qi, Xiaodan Zhang, Guoping Liu

**Affiliations:** 1 State Key Laboratory of Brain Function and Disorders, Ministry of Education Frontiers Center for Brain Science, Institutes of Brain Science, Department of Rehabilitation, Huashan Hospital, Fudan University, Shanghai, China; 2 Department of Neuroscience and Kavli Institute for Neuroscience, Yale School of Medicine, New Haven, CT USA; 3 Department of Central Laboratory, Affiliated Hospital of Hebei University of Engineering, Handan Hebei, China; 4 Center for Clinical Research and Translational Medicine, Yangpu Hospital, School of Medicine, Tongji University, Shanghai, P.R. China; 5 Neurovascular Center, Changhai Hospital, Institute of Neuroscience, Key Laboratory of Molecular Neurobiology, NMU, Shanghai, China; 6 Chinese Institutes for Medical Research (CIMR), Beijing, China; 7 Obstetrics & Gynecology Hospital of Fudan University, Shanghai Key Lab of Reproduction and Development, Shanghai Key Lab of Female Reproductive Endocrine Related Diseases, Shanghai, China; 8 State Key Laboratory of Neurology and Oncology Drug Development, Nanjing, China

**Keywords:** Gliogenesis, Glial development

## Abstract

Neural functions and circuit formation rely on intricate crosstalk among various cell types during critical periods. Disruptions or delays in this crosstalk between neurons and astrocytes lead to abnormal neural functions and neurodevelopmental disorders. However, the lack of robust mouse models to study the crosstalk between astrocytes and neurons thus renders unclear the implications of impeding such interactions. In this study, we demonstrate that *Egfr* knockout during the critical period of neuronal maturation results in a transient absence of astrocytes, with recovery observed in adult mice. This model thus provides a unique opportunity to investigate the effects of impaired astrocyte-neuron communication during development. Mechanically, we show that loss of *Egfr* disrupts the *Egfr-pERK-Epb41l2* signaling axis, which in turn prevents glial progenitor cells from migrating outward. More importantly, *Egfr* deficiency during the critical period compromises astrocyte-neuron communication via the *Sema6a-Plxna2/4* ligand-receptor pair. This impaired intercellular crosstalk reduces neuronal dendritic complexity and excitability, ultimately culminating in depressive-like behaviors in adult mice.

## Abstract (web summary)

This study shows that developmental disruption of EGFR signaling leads to transient astrocyte loss and impaired astrocyte–neuron crosstalk. Altered EGFR–pERK–EPB41L2 signaling and Sema6a–Plxna2/4 communication, results in reduced dendritic complexity, neuronal excitability, and long-term depressive-like behaviours.

## Introduction

The development of the nervous system is finely orchestrated, with various cell types being generated at the right time and space, involving dynamic interactions and crosstalk among each cell type^1–3^. The interaction between neurons and astrocytes is particularly critical for the functional integrity of neural networks. These interactions are mediated by a diverse repertoire of molecular and structural components, ranging from membrane-bound and functional proteins to small signaling compounds, the extracellular matrix, membrane projections, and extracellular vesicles^4^. Among these, the specific interactions mediated by signaling molecules are termed cell-cell communication^4^. In cell-cell communication, crosstalk frequently occurs between signaling pathways activated by distinct ligand-receptor pairs^5^.

Neurogenesis peaks during the embryonic stages, followed by gliogenesis in the postnatal period^6^. During the first two weeks of mouse life, neural circuit activity and the crosstalk between neurons and glia shape the morphological properties of neurons, leading to permanent changes in neural structure and function^7–10^. In this critical period, astrocytes expand and fill the cortex, closely interacting and crosstalking with neurons to form functional circuits^10,11^. The interaction between astrocytes and neurons is a multifaceted process that involves complex signaling mechanisms^12–17^. Astrocytes respond to neuronal activity by releasing gliotransmitters, such as glutamate and ATP, which modulate synaptic transmission and plasticity^10,11,13–15^. In turn, neurons can signal back to astrocytes through neuromodulators, creating a bidirectional communication loop that fine-tunes neural circuit function^17,18^. Deep-layer cortical neurons govern the molecular and morphological specialization of astrocytes via secreted Sonic hedgehog, thereby coordinating circuit assembly^19^. Among the various modes of communication, direct contact between astrocytes and neurons stands out as a pivotal mechanism, allowing the exchange of signals and resources^11^. This intricate dance of signaling molecules forms the basis of the astrocyte-neuron unit, a functional entity that is crucial for brain homeostasis, learning, memory, and cognitive processes^10,11,13–15^.

**Fig. 1:**
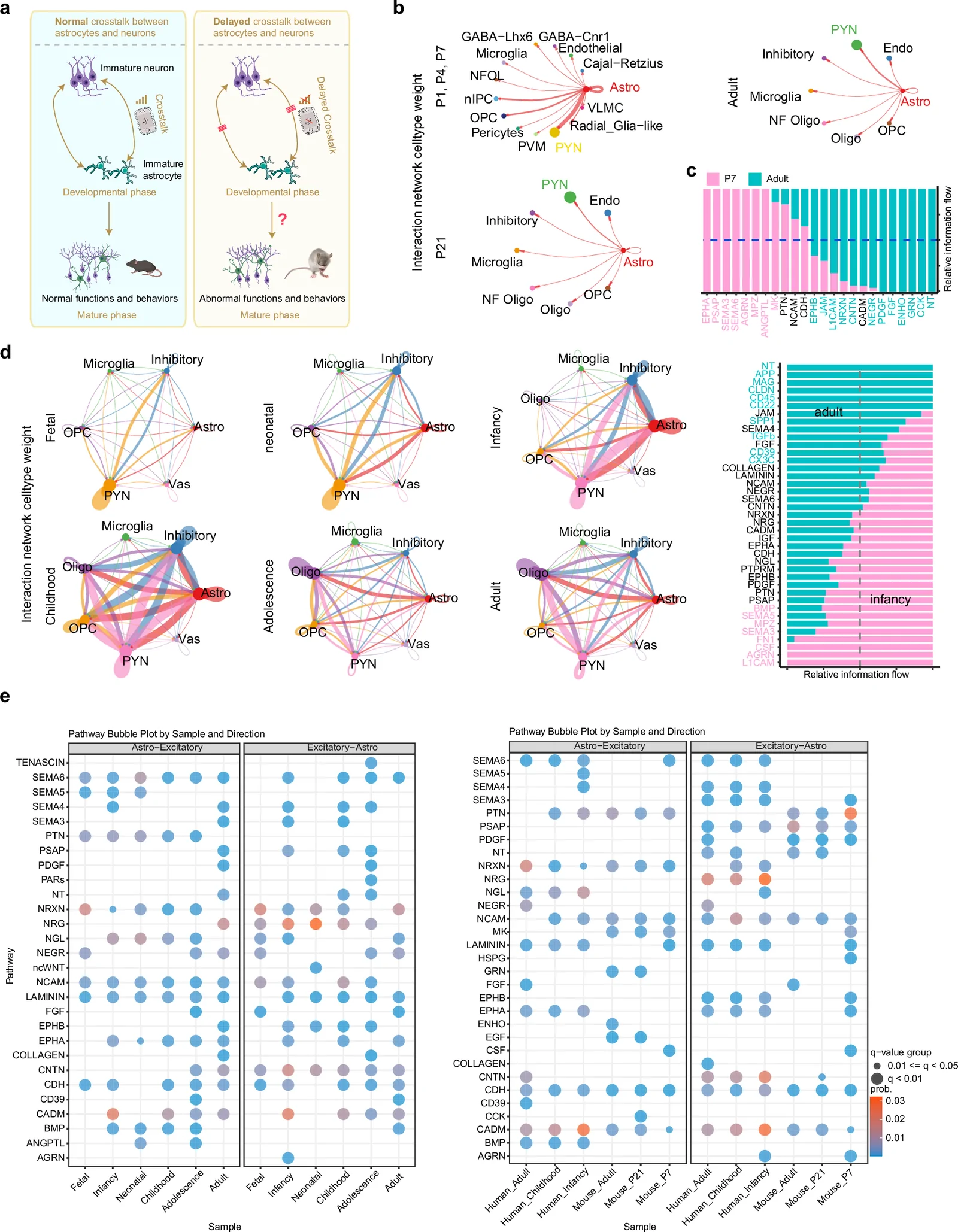
Astrocyte-neuron interaction during early postnatal development in human and mice. **a** Illustration of the crosstalk between astrocytes and neurons. (Created in BioRender. Liu, G. (2026) https://BioRender.com/79dz396). **b** Cell communication analysis of P1, P4, and P7 prefrontal cortex. **c** Bar plot illustrating the specific signaling pathways between the P7 and adult cortex. **d** Circular plots depicting the interaction strength among each cell type in the human prefrontal cortex across different stages, with specific signaling pathways between infancy and adulthood shown on the right. The bar plot on the right shows the pathways involved in astrocyte-neuron communication across all stages of human development. **e** Bubble plot presenting a comparative analysis of pathways involved in astrocyte-neuron interactions between human and mice (*P*-values were derived from one-sided permutation tests with Benjamini-Hochberg correction (see Suppl. Data 1–3 for *q*-values). OL oligodendrocyte, OB olfactory bulb interneuron. OPC Oligodendrocyte precursor cells, EP ependymal cells, Vas vascular cells, nIPC neuron intermediate progenitor cells, PVM perivascular macrophages, VLMC vascular leptomeningeal cells.

Studies have revealed that astrocytes are involved in a wide range of physiological and pathological processes^12,18,20–26^. Disruptions in astrocyte-neuron interactions have been implicated in various neurological disorders, such as Alzheimer’s disease, Parkinson’s disease, and epilepsy, highlighting the importance of understanding this cellular crosstalk for therapeutic interventions^18,23–26^. However, these studies mainly focus on functions observed in adulthood. Due to the lack of robust mouse models, the effects of abnormal astrocyte-neuron interactions during development on neurodevelopment and the potential for mental illness remain unclear (Fig.1a).

In this work, we show a mouse model that allows visualization of the delayed astrocyte-neuron interaction during development. Using this model, we demonstrate that this developmental defect leads to alterations in neuronal functional properties in adulthood and results in specific behavioral abnormalities in mice.

## Results

### Astrocytes closely crosstalk with neurons during infant period

Astrocytogenesis begins at embryonic day 16 (E16) and peaks around perinatal period, occurring in a random manner^6,27–29^. We show that radial glia cells (RGCs) give rise to cortical astrocytes in a lateral-to-medial pattern during the postnatal period (Suppl. Fig. 1a–c). During astrocyte division and expansion, the outgrowth and elaboration of axons and dendrites in cortical pyramidal neurons (PyNs) also occur^17^ (Suppl. Fig. 1d, e). To investigate the molecular basis of bidirectional signaling, we profiled the crosstalk between astrocytes and neurons by mapping intercellular communication networks from single-cell RNA-sequencing data. Notably, astrocytes exhibit close interactions with neurons before P7; after P21, however, their communication dynamics with neurons become similar to those with other cell types (Fig. 1b, and Suppl. Fig. 1e, f, and Suppl. Data 1). During infancy, this crosstalk primarily supports neurogenesis (e.g., SEMA signaling in neurite outgrowth^30^, MPZ signaling in myelination^31^), whereas in adulthood, it is associated with neuroprotection (e.g., PDGF signaling^32^), stem cell regeneration (e.g., FGF signaling^33^), and immunoregulation (e.g., CCK signaling) (Fig. 1c). Certain ligand-receptor pairs, such as NCAM signaling^34^, and EPHB signaling^35^, persist across all stages, promoting neuronal communication and neural circuit maintenance (Fig. 1c). Interestingly, crosstalk between different cell types in the human prefrontal cortex (PFC) exhibits a dynamic pattern as development progresses (Fig. 1d, and Suppl. Data 2). During the fetal stage, PyNs mainly communicate with inhibitory neurons. Starting in the neonatal stage, PyNs begin to interact closely with astrocytes, reaching a climax during the infancy and childhood, which is critical time window for dendritic growth and branching^36^, similar to the pattern observed in mice (Fig. 1b, d). Notably, myelination-related signaling is significantly enriched in the adult human cortex compared to mice, suggesting that myelination in the adult human cortex is more dynamic and active (Fig. 1d).

During human development, astrocyte–neuron interactions display stage-specific features (Fig. 1e). Molecules such as SEMA6, NCAM, and LAMININ are consistently expressed, supporting cell adhesion and axon guidance throughout life^37–39^. Early-stage molecules like SEMA5^40^ (fetal to infancy) and PTN^41^ (persisting into adolescence) are thought to regulate synaptogenesis, neuronal migration, and trophic support, and might be instrumental in driving the formation of initial neural circuits. From infancy onward, NGL and EPHA contribute to synaptic maturation and axon stability^42,43^. It is plausible that molecules like CD39 (regulating ATP metabolism) and CNTN (mediating adhesion), which become prominent during adolescence, could play a role in circuit refinement^44,45^. In adulthood, the network becomes even more complex, incorporating SEMA3/4, PSAP, PDGF, neurotrophins, and EPHB, which together support synaptic remodeling, metabolic coupling, and neural homeostasis^32,35,46–48^.

These interactions are sustained from infancy through adulthood via conserved pathways—NCAM, CADM, NRXN, CDH, and PSAP—though their roles shift over time. Early on, they promote growth and plasticity: facilitating migration, synaptogenesis, angiogenesis, and myelination^49,50^. With maturation, they increasingly stabilize circuits and enable experience-dependent plasticity, supporting lifelong learning and memory^51^. Cell adhesion molecules, especially SEMA3–6, remain central throughout human development, underscoring a focus on precise circuit refinement^30,52^.

**Fig. 2:**
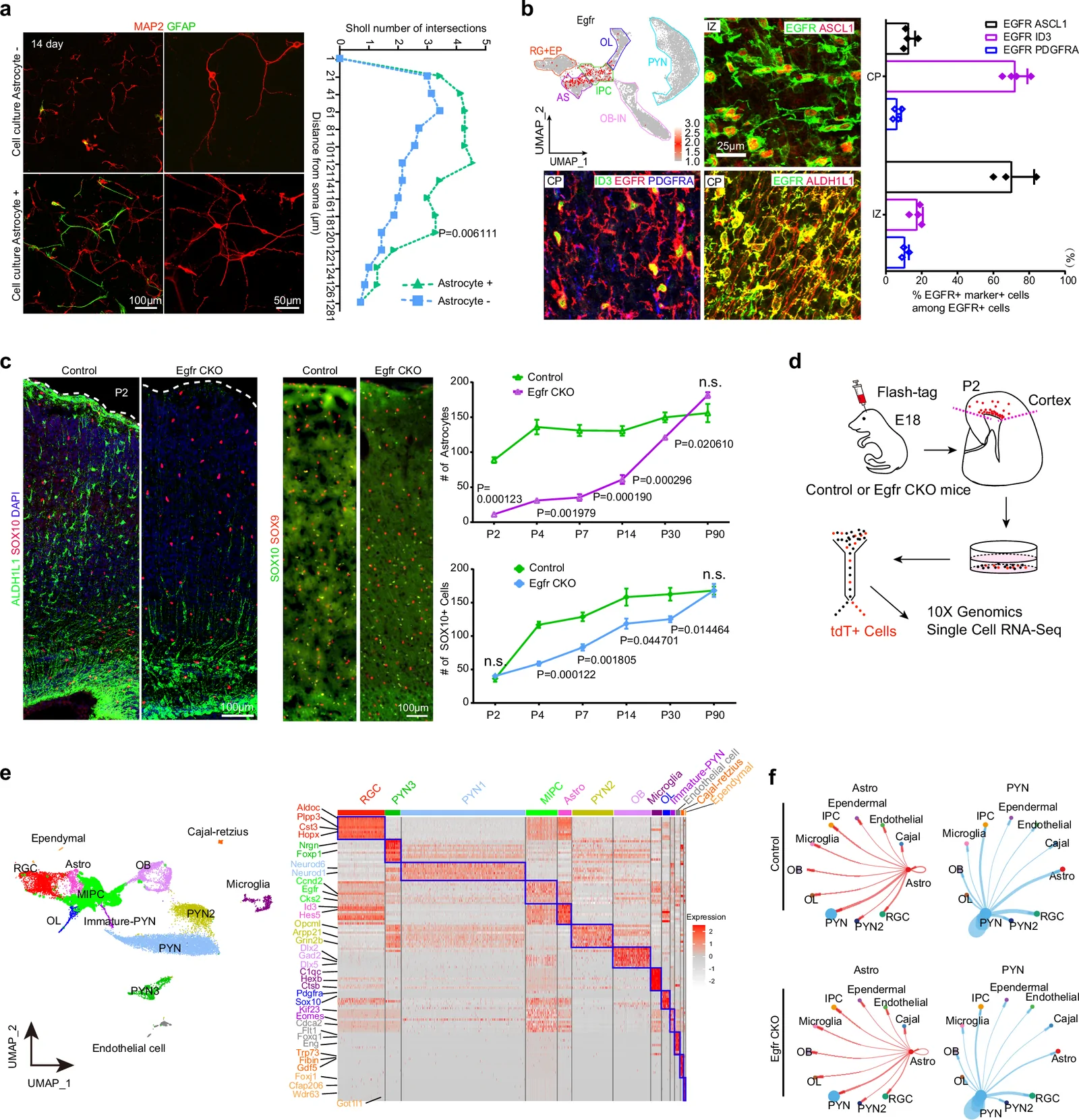
Embryonic EGFR knockout leads to altered astrocytes organization. **a** Left shows primary cell culture of mouse cortex with or without astrocytes co-cultured. Right shows the sholl analysis of cultured neurons (*n* = 7 cells per group, mean values; unpaired two-tailed Student’s *t* test with Welch’s correction). **b** UMAP plot of the expression of *Egfr* for P1 mouse cortex (top left). MIPCs (EGFR ASCL1), APCs (EGFR ALDH1L1 ID3), and OPCs (PDGFRA) in the P1 mouse cortex, and the percentages of the above cells among *Egfr*^+^ cells (right) (*n* = 4 WT mice; EGFR ^+^ ASCL^+^ and EGFR ^+^ PDGFRA^+^ staining, *n* = 3 each, in IZ, mean ± SD). **c** Left shows the expression of ALDH1L1, SOX9, and SOX10 in the cortex of *Egfr* knockout mice and the littermate controls at P2. Right shows the cell numbers per area across P2~P90 (*n* = 4 mice per group (Control: P2–P90, CKO: P2–P30), and 5 mice for CKO P90. mean ± SD; unpaired two-tailed Student’s t test with Welch’s correction). **d** Schematic of single-cell RNA-seq experimental workflow. **e** UMAP plot (left) showing 13 cortical cell types; heatmap showing marker genes for each cell type. **f** Cell chat analysis of the control cortex and the *Egfr-*CKO cortex. CP cortical plate, IZ intermediate zone, RGC radial glia cells, Source data are provided as a Source Data file.

Notable differences exist between humans and mice (Fig. 1e). Humans engage NRG, CNTN, and NGL pathways early to promote pruning and specialization^53–55^, and rely heavily on cell adhesion molecules and ECM components throughout life^56^. In contrast, mouse astrocyte–neuron communication depends more on secreted factors like EGF^57^ and GRN^58^, and metabolic regulators such as ENHO^59^. These differences may reflect distinct evolutionary adaptations: humans prioritize structural stability and metabolic efficiency for complex cognition and longevity^59^, whereas mice emphasize reparative ability and metabolic flexibility for rapid adaptation and survival^60^. To further elucidate the role of crosstalk on neurons, we cultured primary neurons in vitro and found that neurons co-cultured with astrocytes exhibited increased dendritic complexity (Fig. 2a), indicating that astrocyte-neuron crosstalk plays a crucial role in shaping neuronal morphology.

### Delayed crosstalk between astrocytes and neurons modeled by delayed astrocytogenesis

Previous work illustrated that progenitor cells expressing EGFR and ID3 are astrocyte precursor cells (APCs)^6^, and that *Egfr* is required for gliogenesis^61,62^. We observed that EGFR is highly expressed in multipotent intermediate progenitor cells (MIPCs) and persists in astrocyte lineages (Fig. 2b). In addition, the cortex starts to express *Egfr* at around E16, coinciding with the onset of gliogenesis. (Suppl. Fig.2a). This led us to consider whether a conditional mouse allele of *Egfr* could serve as a model for studying crosstalk between astrocytes and neurons. To test this, we generated this mouse allele and crossed it with the *hGFAP-Cre*^63^ mouse to conditionally delete *Egfr* in the stem cells at approximately E13.5 (Suppl. Fig. 2b). As expected, the *Egfr* knockout did not impact the generation or lamination of cortical PyNs, nor did it impact the development of olfactory bulb interneurons (Suppl. Fig. 2c–h). However, the number of astrocytes sharply decreased before the first postnatal month, and recovered in adulthood (Fig. 2c, and Suppl. Fig. 2i, 3a). The effects on oligodendrocytes were significantly mitigated. Therefore, *Egfr* knockout is an attractive model to investigate the interaction between astrocytes and neurons.

To better observe the process of astrocyte recovery, we displayed the entire stained slice across ages (Suppl. Fig. 3a). Interestingly, the recovery of astrocytes in the *Egfr* knockout cortex followed same the lateral-to-medial pattern after birth, as seen during normal development (Suppl. Fig. 1a, 3a). This recovery process was accompanied by an increased proportion of proliferating astrocytes, which exhibited larger cell bodies and nuclei (Suppl. Fig. 3b, c). To further characterize the model, we performed single-cell transcriptome analysis of both the control (All control mice in this study were homozygous for the floxed *Egfr* allele) and the *Egfr* knockout cortices (Fig. 2d, e, f and Suppl. Fig. 4). The number of astrocytes in the *Egfr* knockout cortex decreased significantly compared to the controls within the first two weeks (Suppl. Fig. 4c, and Suppl. Data 3). Additionally, the interaction strength between astrocytes and PyNs was weaker in the *Egfr* knockout cortex (Fig. 2f). The differentially expressed ligand-receptor pairs were primarily associated with cell migration and signal transduction (Suppl. Fig. 4d). Collectively, these findings indicate that embryonic deletion of *Egfr* disrupts crosstalk between astrocytes and neurons due to abnormal astrocyte development during the critical period (infancy), with recovery occurring in adulthood. This makes the *Egfr* knockout model an excellent tool for investigating the consequences of delayed crosstalk between these cell types.

### Migration defects lead to delayed astrocytogenesis via impaired EGFR-pERK-EPB41L2 axis

**Fig. 3:**
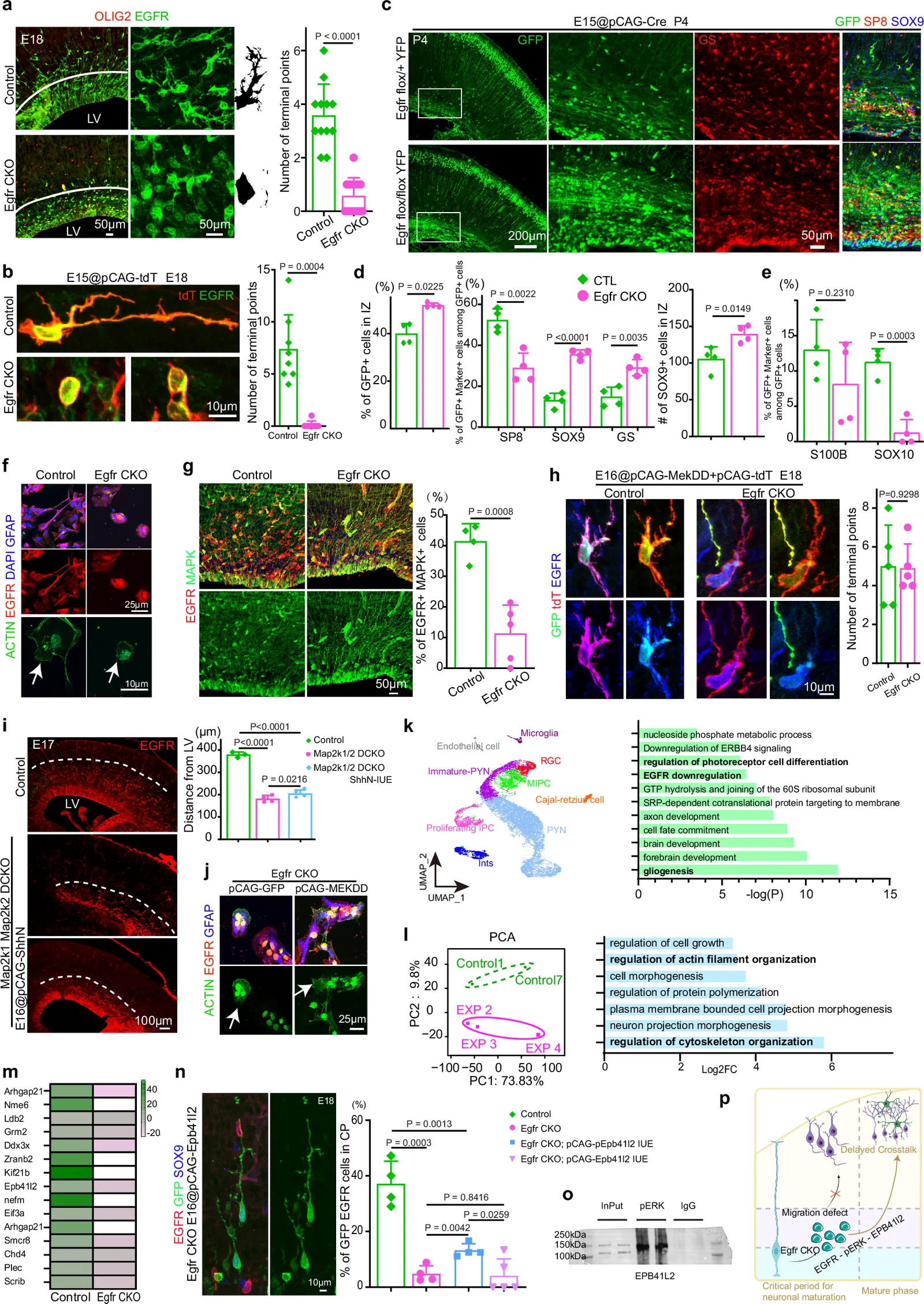
The mechanism by which EGFR knockout leads to delayed crosstalk between astrocytes and neurons. **a** Morphology of EGFR⁺ cortical progenitors in control vs. *Egfr* CKO mice; right: terminal point quantification (*n* = 12 cells from 4 mice). **b** pCAG-tdtomato electroporated at E15, analyzed after 3 days; terminal point quantification (Right, *n* = 8 cells from 3 mice; unpaired two-tailed Student’s *t* test with Welch’s correction). **c**, **d** pCAG-Cre electroporated into *Egfr*^*flox*^ YFP mice at E15, analyzed at P4 (*n* = 4 mice). Percentages of GFP⁺ cells (SP8⁺, SOX9⁺, GS⁺) in IZ; right in (**d**) quantifies SOX9⁺ cells in IZ. **e** pCAG-Cre electroporated into *Egfr*^flox^ H2b mice at E17, analyzed at P21 (*n* = 4 mice); bar plot shows % S100B⁺/GFP and SOX10⁺/GFP cells. **f** P0 neocortex primary culture, analyzed after 3 days (arrows: pseudopodia). **g** EGFR MAPK staining at E18 (left); right shows % MAPK⁺ among EGFR⁺ cells (*n* = 4 control, 5 *Egfr*-CKO mice). **h** Co-electroporation of pCAG-tdtomato and pCAG-*MekDD* at E16, analyzed after 2 days (*n* = 5 mice). **i** Left: EGFR staining in *Map2k1/2* DCKO mice (*n* = 4 mice), and in DCKO mice with ShhN electroporation (*n* = 4 mice), and littermate controls (*n* = 3 mice). Right: maximum migration distance of EGFR⁺ cells from lateral ventricle. **j** pCAG-GFP + pCAG-MEKDD electroporated into *Egfr-*CKO cortex, cultured for 3 days. **k** Flash-tag pulse-labeling of radial glia in DCKO vs. controls at E16, sorted 24 h for scRNA-seq. UMAP and GO terms from MIPCs. **l** P0 cortex phosphopeptide LC-MS; PCA (left) and GO analysis (right). **m** Heatmap of downregulated phosphorylated proteins. **n** Phosphorylated EPB41L2 administered at E16, analyzed after 2 days; left panel, quantification of % GFP⁺EGFR⁺ cells outside IZ (*n* = 4 mice per group, except for IUE *Epb41L2* in *Egfr-*CKO group (*n* = 5)). **o** Co-IP shows pERK binding to EPB41L2. **p** Proposed mechanism(Created in BioRender. Liu, G. (2026) https://BioRender.com/79dz396). Data are mean ± SD; unpaired two-tailed Student’s* t* test with Welch’s correction where needed. Detailed stats in Suppl. Data 5. Representative images/blots from ≥ 3 independent experiments. Source data provided.

To elucidate the mechanism by which *Egfr* deletion delays the crosstalk between astrocytes and neurons, we performed immunostaining to investigate the phenotypes of glial progenitors. Interestingly, glial progenitor cells in the *Egfr* knockout cortex failed to migrate outward, exhibiting a rounded morphology with few or no protrusions, which clearly distinguished them from the control group (Fig. 3a). The conditional *Egfr* knockout allele expresses a truncated, non-functional EGFR protein (lacking a signal peptide), providing an opportunity to trace the progenitor cells (Suppl. Fig. 5a, b). To further confirm the morphological phenotype, we electroporated *pCAG-tdTomato* plasmids (for cell membrane localization) into the cortex (Fig. 3b). We then deleted *Egfr* in a subset of cells via in utero electroporation, and observed that numerous GFP-positive cells (mostly APCs, SOX9^+^ GS^+^) were retained in the subventricular zone and intermediate zone (Fig. 3c, d). The number of GFP-positive glial cells decreased significantly in the adult cortex, suggesting that *Egfr* is crucial for their expansion (Fig. 3e, and Suppl. Fig. 5c–e, g). Similarly, APCs also exhibited a smooth morphology in vitro, failing to form pseudopodia (Fig. 3f, and Suppl. Fig. 5f). We conclude that APCs are unable to migrate in the *Egfr* ablation cortex, failing to contact the neurons and further resulting in reduced interaction with neurons.

How *Egfr* regulates APCs migration? We first examined the *Egfr* downstream target, pERK (MAPK), and found that pERK number were significantly reduced in the *Egfr* knockout cortex (Fig. 3g). Unexpectedly, the expression level of pERK protein was upregulated in the cortex of *Egfr* knockout mice compared to that of the control mice (Suppl. Fig. 6a). This may be due to the upregulation of MAPK expression in the ventricular zone (Fig. 3g). We also observed EGFR expression in the ventricular zone, colocalized with the radial glia cell markers GFAP and SOX9 (Suppl. Fig. 6a, b). This observation prompted us to investigate whether other signaling pathways—such as *Fgf* signaling—might contribute to MAPK activation in the VZ. Both the RGCs and astrocytes expressed high levels of *Fgfr1* and *Fgfr3*, with *Fgf18*^64^ (a ligand capable of activating MAPK signaling through *Fgfr1* and *Fgfr3*) expression upregulated in the postnatal brain, indicating that RGCs may be influenced by Fgf18 signal (Suppl. Fig. 6c). We then expressed *Fgf18* or the constitutively active form of *Mek* in the cortex and found that FGF18 can diffuse widely, and both were able to rescue the phenotypes of migration defects and morphological defects in the *Egfr*-CKO cortex (Fig. 3h, j, and Suppl. Fig. 6e, f).* Map2k1/2*double knockout mice phenocopied the *Egfr* deleted cortex (Fig. 3i). Collectively, these results demonstrate that MAPK signaling plays a central role in the *Egfr* signal cascade (Suppl. Fig. 6g).

To investigate the downstream targets of pERK, we performed single-cell RNA sequencing on a *Map2k1/2* double knockout cortex (Fig. 3k, and Suppl. Fig. 7). Gene Ontology analysis showed that differentially expressed genes (DEGs) were significantly enriched in gliogenesis, forebrain development, and EGFR downregulation, in accordance with observations in *Egfr* knockout mice (Fig. 3k, and Suppl. Fig. 6d, 7c). Furthermore, the *Map2k1/2* CKO cortex exhibited a reduction in communications between astrocytes and neurons (Suppl. Fig. 7e, f). We conducted a comprehensive analysis and functional testing of the commonly shared DEGs of the MIPCs in both *Map2k1/2* double knockout cortex and *Egfr* knockout cortex, but failed to identify a clear key downstream target (Suppl. Fig. 7d). Therefore, we enriched phosphorylated proteins followed by Liquid chromatography–mass spectrometry (LC-MC) (Fig. 3l, and Suppl. Fig. 8a-d). Sixty-five differentially expressed phosphorylated proteins were identified, and most of which were involved in cell morphology, such as cytoskeleton and filament organization (Fig. 3l). Using exhaustive search, we tested down-regulated proteins and found that EPB41L2, which is relevant to the actin cytoskeleton, could partially rescue the altered phenotypes observed in the *Egfr* ablation cortex (Fig. 3m, n, and Suppl. Fig. 8d, e). Co-immunoprecipitation showed that pERK directly binds to EPB41L2 protein (Fig. 3o). Taken together, due to the impaired EGFR-pERK-EPB41L2 axis, APCs cannot migrate outward, thereby failing to contact the neurons and resulting in delayed crosstalk with neurons (Fig. 3p).

### Delayed crosstalk between astrocytes and neurons leads to depressive-like behaviors

What is the impact of delayed crosstalk on neuronal function and mouse behavior?

Guided by the “lateral-medial” developmental pattern of astrocytes, we focused our investigation on neurons in the medial prefrontal cortex(mPFC) to explore the cell-type-specific mechanisms by which astrocytic changes impact neuronal circuits.

**Fig. 4:**
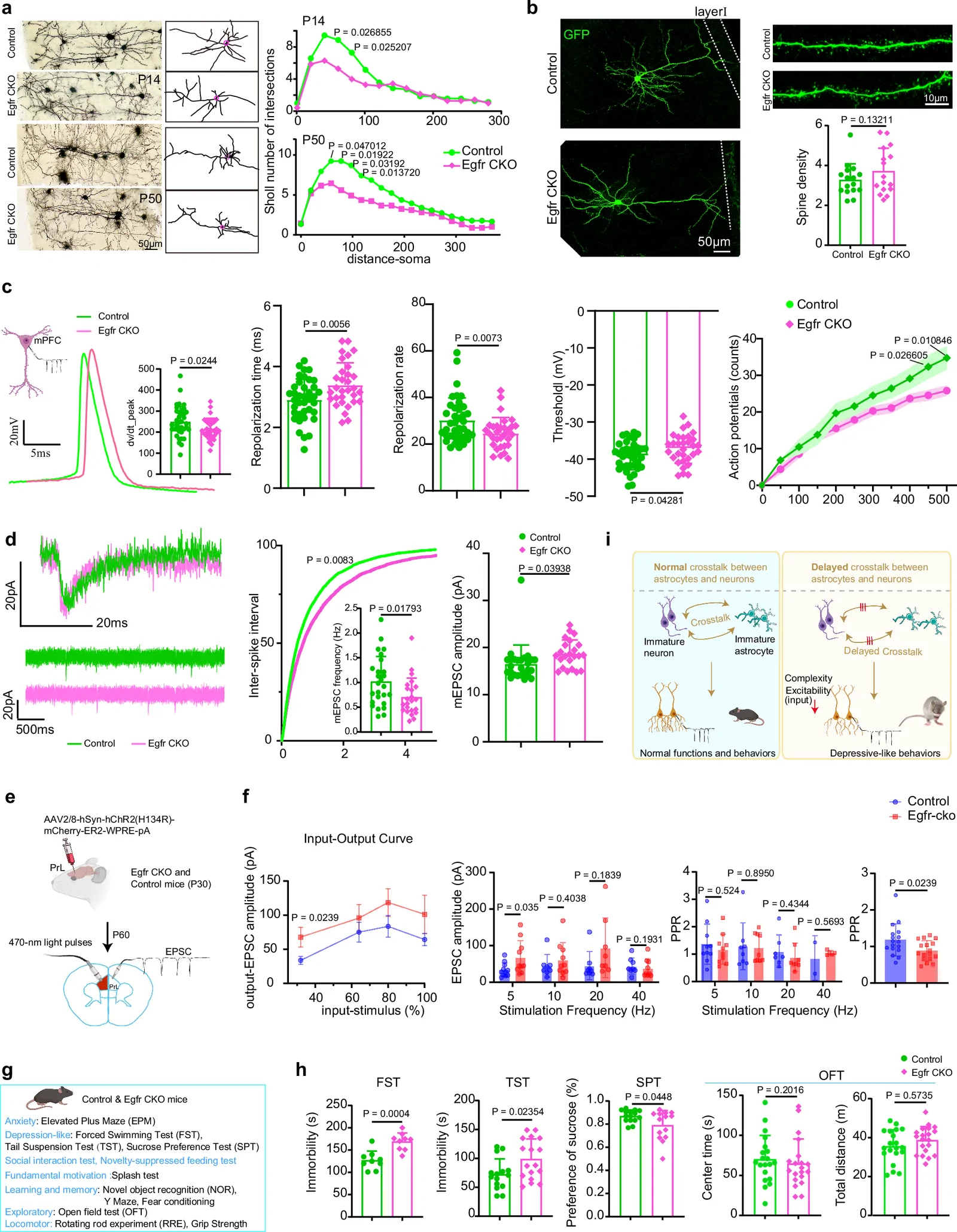
Delayed crosstalk between astrocytes and neurons leads to depressive-like behavior in mice. **a** Golgi-Cox staining of *Egfr* CKO cortex and the littermate controls at P14 (*n* = 7 cells per group) and P50 (*n* = 9 control cells and 7 *Egfr*-CKO cells, mean ± SD; unpaired two-tailed Student’s *t* test). Sholl analysis of the PyNs (right). **b** Biocytin-filled layer Ⅴ PyNs and spine density at P50 (*n* = 16 cells, mean ± SD; unpaired two-tailed Student’s *t* test). **c** Representative traces of action potentials of layer Ⅴ PyNs in the *Egfr* CKO and the control prefrontal cortex at P50. Quantification data represent summary data (*n* = 38 cells from 6 control mice and 31 cells from 5 *Egfr*-CKO mice; mean ± SD; unpaired two-tailed Student’s t test, with Welch’s correction applied for dv/dt). **d** Representative traces of mEPSCs, along with inter-spike interval and amplitudes of mEPSCs (*n* = 4 control mice and 3 *Egfr*-CKO mice; mean ± SD; unpaired two-tailed Student’s t test with Welch’s correction). **e** Optogenetic experimental design. **f** Short-term synaptic dynamics (*n* = 3 mice, mean ± SD; unpaired two-tailed Student’s t test with Welch’s correction). The input-output relationship is depicted in the line chart (left), while the bar plot (center) shows the response amplitude and paired-pulse ratio (PPR) at 32% intensity across various stimulation frequencies, and PPR at 5 Hz/80% intensity (right). **g** Experiments used to detect behavioral tests. **h** Performance of Egfr CKO mice and the controls in the tests mentioned at (**g**) on 2 months (For FST and OFT, *n* = 9 and 20 mice per group, respectively; 15 control mice and 17 *Egfr*-CKO mice for TST; 15 control mice and 14 *Egfr*-CKO mice for SPT; 20 mice per group in OFT; mean ± SD; unpaired two-tailed Student’s *t* test, with Welch’s correction applied for SPT). **i** Diagrams illustrating how delayed crosstalk between astrocytes leads to reduced dendritic complexity and neuronal excitability, contributing to depressive behaviors. Detailed statistics are in Suppl. Data 5. Source data are provided as a Source Data file.

We found that the PyNs in the *Egfr*-deleted PFC exhibit significantly reduced dendrite complexity, while the density of dendritic spines remained unchanged (Fig. 4a, b). We performed ex vivo whole-cell patch clamp recordings of PyNs from the PFC of *Egfr* CKO and the littermate control mice. These recordings revealed a reduced frequency of miniature excitatory postsynaptic currents (mEPSCs) (Fig. 4d), suggesting that the neurons in *Egfr* CKO mice received less complex excitatory input. Furthermore, the neurons showed an increased threshold and a decreased action potential firing rate, indicating reduced neuronal excitability in the *Egfr* CKO mice (Fig. 4c). Changes in other intrinsic properties of the neurons were also observed, including a decreased repolarization rate, although resting membrane potential and rheobase were unaffected (Fig. 4c, d, and Suppl. Fig. 9a). Interestingly, we observed an increase in mEPSC amplitude despite an overall decrease in synapse number (as inferred from reduced dendritic complexity and unchanged synaptic density), implying a potential enhancement in the functionality of the remaining synapses^65^ (Fig. 4d).

To further elucidate synaptic connectivity and network dynamics, we assessed the strength of callosal inputs using an optogenetics approach (Fig. 4e). We found that oEPSC amplitude was increased while PPR was decreased (Fig. 4f). This, together with the higher proportion of neuronal activation observed at low light intensity (Suppl. Fig. 9b, c), supports the conclusion that neurons in the *Egfr* CKO PFC exhibit heightened sensitivity to inputs, reflecting increased presynaptic release probability and enhanced postsynaptic response gain. This suggests that *Egfr* CKO, while impairing the neuron’s structural foundation, triggers a compensatory response via homeostatic plasticity that selectively potentiates the remaining synaptic connections. Furthermore, the RNA sequencing data of the adult prefrontal cortex showed that no significant changes were detected in the expression of genes associated with NMDA receptors, AMPA receptors, or PSD95 in the *Egfr* CKO cortex (Suppl. Figs. 9d, 10b, c). Immunohistochemical analysis pointed towards a post-synaptic strengthening, revealing brighter and larger PSD95 puncta in *Egfr* CKO neurons (Suppl. Fig. 9e, f). This suggests a consolidation of PSD95 protein within individual postsynaptic densities, despite western blotting showing only a non-significant trend towards increased total PSD95 levels. The morphological evidence of synaptic redistribution aligns with the functional enhancement observed in mEPSC amplitude, supporting the conclusion of potentiated remaining synapses. Collectively, these findings indicate that *Egfr* CKO disrupts neuron–astrocyte interactions, leading to impaired structural connectivity (as reflected by reduced dendritic complexity), which in turn triggers functional compensation through strengthening of remaining synapses. Ultimately, this compensatory remodeling disrupts the normal balance of the network.

The transcriptome data showed that the glycosaminoglycan biosynthesis pathway, which is related to neurite growth^66,67^, ranked first (Suppl. Fig. 10b, c). Most DEGs were highly expressed in the PyNs and the endothelial cells, but not in the astrocytes or oligodendrocytes; meanwhile, no significant differences were observed in the morphology of astrocytes and oligdendrocytes and extracellular ATP or in the myelination pattern in the cortex, indicating that the delayed communications primarily affect the morphology and function of neurons, rather than glial cells (Suppl. Fig. 10c–g, and Suppl. Data 4).

A spectrum of behavioral tests, assessing the cortex’s roles in motor control, learning, memory, and emotion, demonstrated that EGFR-CKO mice had largely normal motor and learning functions (Suppl. Fig. 9g), but displayed significant emotional disturbances. Forced swim test (FST) and tail suspension test (TST) measure a shift from active coping (e.g., climbing/struggling) to passive coping (immobility), reflecting behavioral despair rather than direct depression modeling^68,69^. The Sucrose Preference Test (SPT) was performed to assess anhedonia, a core symptom of depression that is characterized by a reduced preference for a sucrose solution over plain water^70^ (Fig. 4g, h, and Suppl. Fig. 9g). To determine whether these changes were permanent, we assessed neuronal morphology and conducted behavioral tests on *Egfr* CKO mice at 6 months of age. We found that the experimental group exhibited reduced dendritic complexity and displayed depressive-like tendencies (Suppl. Fig. 9h, i). From a functional anatomical standpoint, the mPFC is a primary hub that integrates emotion and cognition^71^. Growing evidence suggests that mPFC dysfunction is a key risk factor for depression, which manifests at the molecular level as a failure of neuroplasticity, involving neuronal atrophy and synaptic suppression in this region^72–74^. The behavioral results further validated the functional deficits in the mPFC. Besides this, *Egfr*-CKO mice increased the percentage of open arm entries; it did not alter the percentage of time spent in the open arms (Suppl. Fig. 9g). Further analysis of risk-assessment behaviors indicated that this pattern reflected more frequent, shorter exploratory forays into the aversive open arms, characterized by increased investigative activity (e.g., unprotected head-dipping) upon entry, rather than general hyperactivity (Suppl. Fig. 9g). We therefore interpret the increased open arm entries as a specific reduction in anxiety-like behavior.

Taken together, these findings indicate that delayed crosstalk between astrocytes and neurons leads to reduced dendritic branching complexity, diminished neuronal excitability, and ultimately, depressive-like behaviors (Fig. 4i).

### Astrocytes and neurons crosstalk through *Sema6a-Plxna2/4* ligand-receptor pair

Delayed crosstalk between astrocytes and neurons decreases the complexity and excitability of neurons, leading to depressive-like behaviors. How this delayed crosstalk has such an impact on neurons is worth exploring. Among various crosstalk mechanisms, ligand-receptor interactions (LRIs) represent the predominant mode of regulation. In LRIs, a ligand secreted by one cell binds to its cognate receptor on another, initiating intercellular signal transduction^4^. To explore their interactions, we applied Cellchat on a single-cell RNA sequencing dataset spanning developmental stages from P1 to P7 (Suppl. Figs. 1f, 11a). For an extrinsic signal from astrocytes to elicit the neurogenic effects described above, it must meet the following criteria: first, the receptor must be expressed in the neurons; second, the ligand must be exclusively expressed in immature astrocytes during the critical period of neuron maturation, and not in neurons; third, this ligand-receptor interaction necessitates the elicitation of downstream changes that lead to morphological alteration of neurons.

**Fig. 5:**
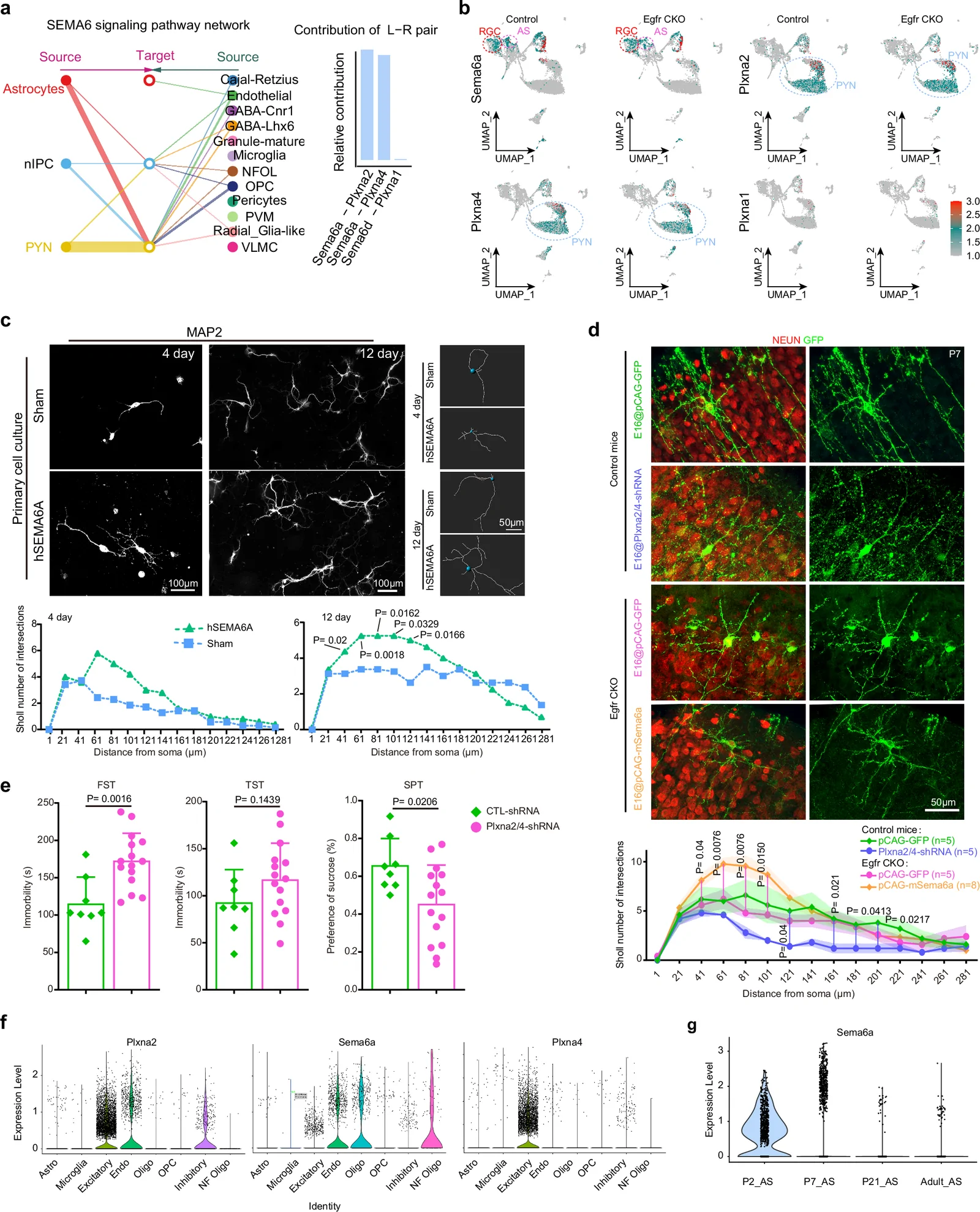
Astrocytes promote neuronal dendrite branching through the SEMA6A signal. **a** The inferred SEMA6 signaling network, and the computed network centrality measures of SEMA6 signaling. **b** UMAP plot showing the expression of genes involved in SEMA6 signaling. **c** The perinatal cortex was dissected for primary neuron culture, and human SEMA6A protein was administered on day 7 and day 2, and analyzed on day 12 and day 4 respectively (*n* = 7 cells (sham) vs 6 cells (hSEMA6A, 4 d) and 8 cells per group at 12 d; mean values; unpaired two-tailed Students’t test with Welch’s correction). **d** *Plxna2/4*-shRNA plasmids and control plasmids were electroporated into the cortex of wild-type mice. Additionally, plasmids overexpressing *mSema6a* were transfected into the EGFR-CKO cortex at E16 and analyzed at P7. Bottom shows the sholl analysis of GFP positive neurons (with line colors consistent for each group) (mean ± SD; unpaired two-tailed Student’s t test, with Welch’s correction applied for control group). **e** *Plxna2/4*-shRNA or control plasmid were electroporated into the mPFC region of mice at E13, and the test relating to depression were performed from P60 (*n* = 8 mice (control), 15 mice (*Plxna2/4*-shRNA), mean ± SD; unpaired two-tailed Student’s *t* test). **f** Violin plot depicting the expression of *Plxna2/4* and *Sema6a* in the adult cortex. **g** Astrocytes extracted from P2 to adulthood were analyzed. Figure shows the expression of *Sema6a* in a violin plot. Source data are provided as a Source Data file.

Screened by the criteria mentioned above, we focus on the SEMA6 signaling pathway, where the ligand *Sema6a* is highly expressed in astrocytes and RGCs, while the receptors *Plxna2/4* are mainly localized in PyNs (Fig. 5a, b, and Suppl. Figs. 11a,12a). To evaluate the function of SEMA6 signaling, we exposed primary neuron cultures to human SEMA6A protein in vitro and expressed mouse *Sema6a* (mSema6a) in the EGFR knockout cortex in vivo. We found that SEMA6A significantly increased the dendritic complexity of neurons either in vitro or in vivo (Fig. 5c, d). Furthermore, knockdown of *Plxna2/4* via in utero electroporation obviously reduced dendritic complexity, consistent with previous reports that loss of Plxna4 function results in dramatic alterations to dendrite process complexity^75,76^ (Fig. 5d). More importantly, adult mice with knockdown of *Plxna2/4* in the mPFC exhibited depressive-like behaviors and showed no significant alterations in synaptic plasticity, as assessed by LTP (Fig. 5e, and Suppl. Fig. 12b). In addition, we observed that neurons originating from distinct regions within the same culture dish exhibited significantly increased area coverage when in contact with astrocytes, thereby underscoring the pivotal role of such direct interactions and supporting the significance of SEMA6 signaling, specifically *Sema6a* as a transmembrane semaphorin, in the model of delayed crosstalk (Suppl. Fig. 12c). Notably, the *Sema6a-Plxna2/4* expression was elevated in the *Egfr*-deleted cortex, further implying that the delayed migration of astrocytes results in delayed communication with neurons (Suppl. Fig. 4d).

We then try to explain why the recovered astrocytes fail to restore the morphological changes of neurons. The expression of receptor *Plxna2/4* persists in adult PyNs neurons, while the ligand *Sema6a* is exclusively expressed in infant astrocytes and its expression ceases after P21 (Fig. 5f, g). Although *Sema6a* is expressed in a subset of PyNs and oligodendrocytes in the adult cortex, the morphological alterations in neurons failed to revert, suggesting that either the signal from astrocytes is indispensable, or the neurons have surpassed their critical period for plasticity, or else both factors are at play (Fig. 5b, f and Suppl. Fig. 12a). Astrocytes in the infant brain mainly express genes relating to RNA processing, widely express various ligand-receptor pairs and closely crosstalking with neurons, while in later stages, astrocytes primarily express genes enriched in metabolic processes, implying that immature astrocytes play a crucial role (Fig. 1b, and Suppl. Fig. 1f, 12d). Given that dendritic growth and branching of neurons occur during the infant period^36,77^, and considering the temporal expression pattern of *Sema6a* in astrocytes, we conclude that the impaired crosstalk between astrocytes and neurons during the critical period, bears primary responsibility for failing to restore the morphological changes of neurons in the adulthood.

### Mendelian randomization link astrocytes to major depressive disorder (MDD)

**Fig. 6:**
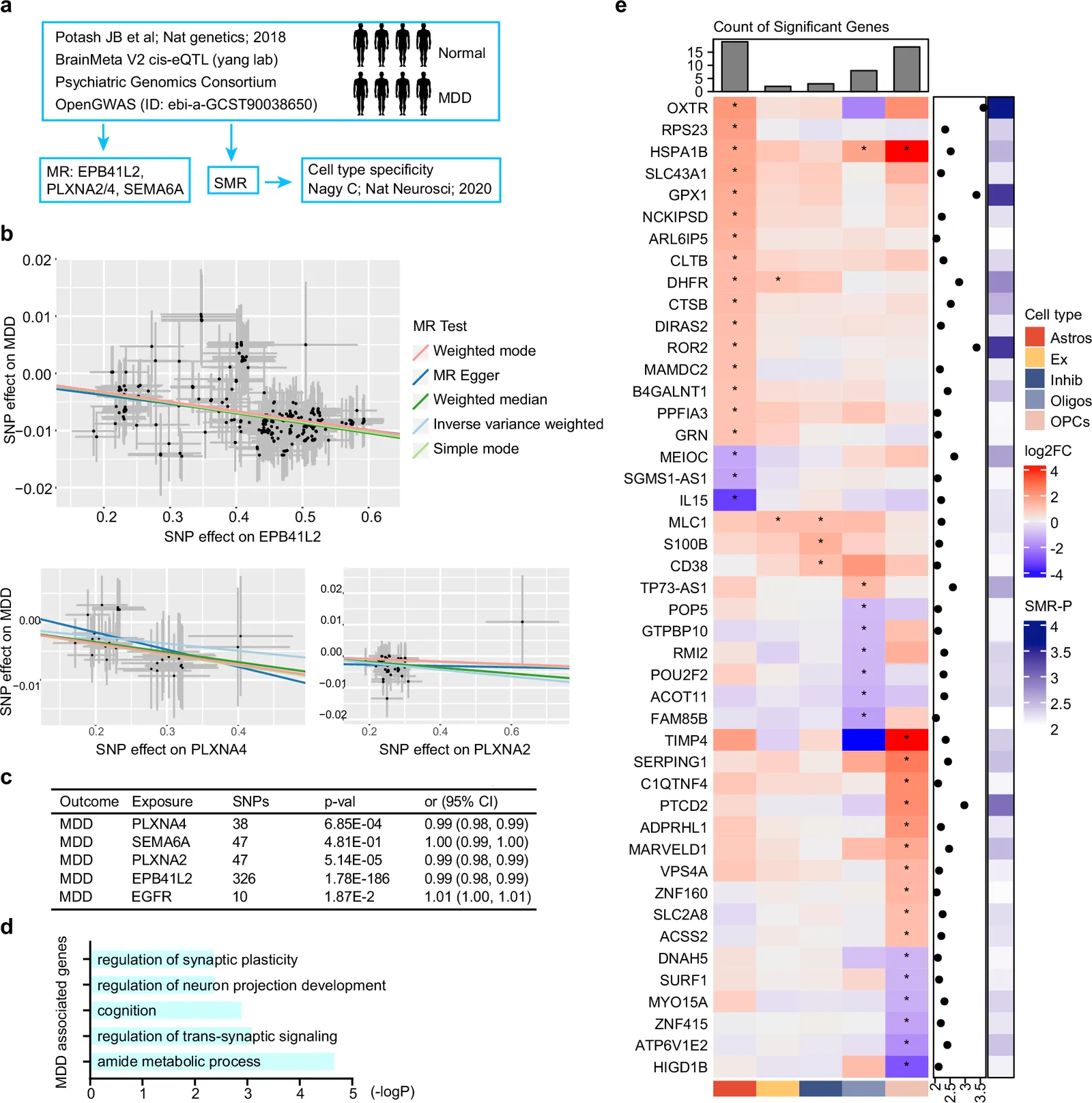
Clinical data indicate that genes related to the development and function of astrocytes are associated with major depressive disorder (MDD). **a** Workflow of mendelian randomization (MR) analysis. **b** Scatter plot of individual single-nucleotide polymorphism (SNP) effects (EPB41L2, PLXNA2, PLXNA4) and estimates from different Mendelian randomization methods for the effect of MDD. **c** Inverse-variance weighted estimates for the association between a genetically determined unit increase in exposure on the risk of MDD. **d** Genes that were both significantly associated with MDD and differentially expressed in MDD brains were used for GO analysis. **e** Heatmap depicting fold changes in single-cell pseudo-bulk expression, along with Summary-data-based mendelian randomization (SMR) analysis, is presented for MDD. This heatmap displays the log2 fold change in pseudo-bulk expression levels across various cell types. Asterisks (*) are used to denote genes that exhibit significant alterations between control and MDD groups. The top bar indicates the count of significant genes, whereas the sidebar represents the SMR *p*-values associated with these genes.

Migration defects of APCs lead to impaired crosstalk with neurons, contributing to depressive-like behaviors in mice. To assess the applicability of this model in depression, we conducted Mendelian randomization using clinical data^78–80^ from MDD (Fig. 6a). GWAS summary statistics for MDD, including 135, 458 cases and 344, 901 controls, were obtained from the Psychiatric Genomics Consortium^81^, the largest consortium in psychiatry. Our analysis revealed that the downregulation of EPB41L2, PLXNA2, and PLXNA4 is significantly associated with MDD (Fig. 6b, c). No evidence of heterogeneity and sensitivity were found in the MR analysis (*P* > 0.05). These data further suggest that delayed crosstalk between astrocytes and neurons contributes to depression both in mice and humans.

Informed by the results above, we sought to test the connection between astrocytes and depression. By using brain eQTL summary data as the exposure and MDD GWAS data as the outcome, we identified 850 SNPs across 850 genes that were significantly associated with MDD. Additionally, differential expression analysis using scRNA-seq data^82^ revealed changes in gene expression across cell types between control and MDD groups. A total of 45 genes were both significantly associated with MDD and differentially expressed. GO analysis indicated that these genes are mostly related to amide metabolic processes and synaptic functions (Fig. 6d). Notably, a large portion of these genes (19/45) were linked to astrocytes (Fig. 6e). Collectively, these findings highlight the critical role of astrocytes in MDD.

## Discussion

Astrocytes regulate synaptogenesis and synaptic pruning, and neuronal plasticity, which are essential for shaping neural circuits^12–16,20,22^. These processes depend on close crosstalk between astrocytes and neurons during critical periods; however, the precise nature of their interactions and the resulting consequences remain unclear. This work demonstrated that *Egfr* knockout mice can serve as an excellent model to study the crosstalk between astrocytes and neurons. In these mice, the APCs fail to migrate outward due to disruption of the EGFR-pERK axis, preventing their contact with PyNs during infancy. As a result, the neurons exhibit reduced neuronal excitability and decreased dendritic complexity in adulthood, resulting in depressive-like behaviors. (Suppl. Fig. 13). Neuronal alterations are a key pathological basis of psychiatric disorders. In autism spectrum disorder (ASD), dysfunction of actin filaments disrupts neuronal connectivity^83^, while major depression involves alterations in dendritic morphology and selective neuronal loss^84–86^. Research also indicates that early-life inflammation can induce depression by promoting excessive microglial engulfment of neuronal spines^87^.

Recently, there have been increasing studies focusing on the role of astrocytes in neural circuit assembly. Traditional approaches, such as genetic targeting using astrocyte-specific promoters or viral vectors for stereotactic delivery, have limitations, including a lack of temporally and cell type-specific astrocyte markers^88,89^. This study highlights the crucial role of astrocytes during the infancy period and their direct interplay with neurons. However, we have yet to determine whether all immature astrocytes or a specific subset contribute to dendritic development. Maybe some subtype-specific markers of astrocytes are needed to address this issue. We show that direct interaction through SEMA6A-PLXNA2/4 ligand-receptor pair is critical for neuronal dendritic development; however, due to the limitations in sequencing depth of single-cell RNA sequencing, some relevant genes may not have been detected, which could still influence the observed effects. In addition, it is important to note that the *hGFAP-cre* driver used in this study is active in radial glia and their progeny^63^. Although the combined evidence strongly points to an astrocyte-specific mechanism, future studies using astrocyte-specific Cre lines, when they become available, would be valuable to conclusively rule out potential contributions from other cell types. Overall, our model offers a promising opportunity to study the direct crosstalk between astrocytes and neurons during the critical period of neural circuit assembly. The limitation of our study is that the developmental results of astrocytes are mostly based on data from the motor and sensory cortex regions, with only a subset of results covering the specific location of the PFC (Suppl. Fig. 3a). Nevertheless, this does not affect our judgment regarding the developmental trend in the PFC region. This is because we found that astrocytes follow a lateral-to-medial gradient in their distribution during development, with the mPFC being one of the last regions where these cells populate.

Cortical PyNs and astrocytes share a common neuroepithelial origin and are generated in a temporally defined manner throughout embryogenesis and perinatal periods^6,90,91^. While the neurogenesis process—comprising generation, migration, and maturation—is well-characterized, gaps remain in our understanding of astrocyte development. Neurogenesis follows an inside-out pattern and neurons migrate along the radial glia fibers, but it remains unknown how APCs migrate outwardly and the mechanisms underlying their migration. Tabata et al. showed that APCs migrate in an erratic, blood vessel-guided pattern, with Cxcr4/7 playing a critical role in this process^92^. Here, we show that the EGFR-pERK-EPB41L2 signaling axis plays a significant, though not exclusive, role in APC morphogenesis and migration. Phosphorylated proteins identified in the P0 cortex are primarily involved in cytoskeleton organization and filament assembly, emphasizing the crucial role of phosphorylation cascades in glial cell morphology and migration (Fig. 3l). However, we cannot rule out the involvement of other genes or signaling pathways, such as those related to cytoskeleton organization or MAPK activation. Further investigations are needed to fully elucidate astrocyte development.

Our study established that EGFR-pERK-EPB41L2 axis as a mechanism for astrocyte-neuron crosstalk in synaptic regulation. Although direct evidence for this specific axis in other neuropsychiatric conditions is sparse, its components are independently linked to key pathologies. ERK/MAPK dysregulation drives synaptic and inflammatory deficits in ASD^93^ and Obsessive Compulsive Disorder(OCD)^94^. EPB41L2 dysfunction disrupts postsynaptic density integrity^95^, and astrocytic EGFR in the amygdala gates fear behavior^96^. Aberrations in regulators of the ERK/MAPK signaling cascade contribute to a spectrum of neurodevelopmental and psychiatric disorders, including ASD, attention deficit hyperactivity disorder, Fragile X syndrome, and Schizophrenia^97^. Thus, we propose that disturbances in this axis may represent a convergent pathway for synaptic dysfunction across disorders. Future studies should validate its role in ASD, OCD, and related conditions, leveraging our findings as a mechanistic foundation.

Numerous hypotheses regarding the causes of depression have been proposed, including deficiencies in neurotransmitters^74^ (e.g., dopamine, serotonin), inflammation^96^, disruptions in neuroplasticity^72^, and disturbances in the microbiota-gut-brain axis. Astrocytes, the most prevalent glial cell in the CNS, play an important role in the pathogenesis of depression, through mechanisms such as astrocyte-mediated neuroinflammation^98^, impaired ATP release^87^, alterations in connexins, and dysfunctional tripartite synapses with neurons. Our RNA-seq data showed that the expression of most serotonin and dopamine receptors (e.g., Htr1a-Htr7, Drd1-Drd5) and the dopamine transporter (Slc6a3) remained unchanged, but we observed a significant upregulation of two key catabolic enzymes, MAOB and COMT. This specific finding suggests a potential disruption in monoamine metabolism rather than direct receptor signaling. Increased degradation of dopamine and norepinephrine by these enzymes may reduce monoamine availability in synaptic clefts, contributing to depressive-like behaviors independently of receptor alterations. Early astrocyte loss might indirectly impair monoaminergic circuits via disrupted glutamate homeostasis or neurotrophic support. However, the role of astrocyte development in depression remains unclear. This study suggests that defects in astrocyte development, leading to impaired neurodevelopment, ultimately result in depressive-like behaviors in mice. Furthermore, genes associated with glial progenitor migration (e.g., EPB41L2) and astrocyte-neuron crosstalk (e.g., PLXNA2/4, SEMA6A) are positively correlated with MDD, supporting the hypothesis that abnormal astrocyte development during critical periods disrupts neural circuit assembly, contributing to psychiatric disorders.

Mendelian randomization study identifies a causal link between EPB41L2, PLXNA2, PLXNA4, and MDD, with potential clinical implications. Firstly, as cell surface receptors, PLXNA2 and PLXNA4 represent ‘druggable’ targets. Therapeutic strategies could be explored to modulate their signaling activities, for instance, by developing monoclonal antibodies or soluble receptor fragments. Secondly, the genetic variants or expression levels of these genes could potentially serve as biomarkers for identifying an MDD subgroup characterized by dysregulated Semaphorin-Plexin signaling, paving the way for stratified medicine in psychiatry. While translating these findings into clinics requires overcoming significant challenges—such as elucidating their precise roles in specific neural circuits and achieving brain-targeted drug delivery—our findings highlight novel and promising avenues for understanding MDD etiology and developing mechanism-based therapeutics.

## Methods

### Animals

All animal procedures were performed in accordance with the institutional guidelines and were approved by the Laboratory Animal Research Center, Shanghai Medical College, Fudan University (No. 20220228-140). All mice were housed under specific pathogen-free conditions with a 12:12 h light-dark cycle (lights on at 7:00 AM), at an ambient temperature of 22 ± 2 °C and relative humidity of 50 ± 10%. Food and water were provided ad libitum. Mice were maintained on a mixed genetic background of C57BL/6 J and ICR strains. *hGFAP-Cre*^63^, *Rosa-H2B-GFP floxed*^99^, *Rosa-YFP floxed*^100^ *mice* were obtained from The Jackson Laboratory. Conditional knockout alleles *Map2k1* (*floxed* exon 2)*, Map2k2* (*floxed* exon 4–9)^101^*, Egfr* (*floxed* exon 2) (Figure S2b) were generated by GemPharmatech Co., Ltd. (Nanjing, China). The date of the vaginal plug was designated as E0.5, while the day of birth was identified as P0. Both male and female mice were used in all experiments. Mice were euthanized by cervical dislocation. All efforts were made to minimize suffering, and death was confirmed by cessation of respiration and lack of response to toe pinch.

### Plasmid construction

The vector backbone was digested with appropriate restriction enzymes. The target gene fragment was amplified with primers containing 15–20 bp homology arms matching the ends of the linearized vector. Homologous recombination technology (Beyotime, D7010M) was employed to ligate the target fragment into the vector. The constructed plasmids were subsequently transformed into F-DH5α competent cells (WEIDI, DF1001). Transformed cells were plated on LB agar containing ampicillin and incubated overnight at 37 °C. Single colonies were picked for plasmid extraction, and the correct construction was verified by Sanger sequencing. For detailed information on plasmid construction, see Suppl. Data 6.

### In utero electroporation

We delivered plasmid to the mouse cortex from E13.5 to P0 via in utero electroporation (IUE). The amount of plasmid was 2 μg of each embryo, which was mixed with 0.05% Fast Green (Sigma). Before electroporation of embryonic mice, the pregnant mouse was anesthetized using isoflurane. A 2 cm incision was made along the midline of the abdomen to expose the mouse uterus. We injected the mixture into the lateral ventricle of embryos using a beveled pulled glass micropipette. For the E13.5 embryos, five electrical pulses (duration: 50 ms) were applied at 31 V across the uterine wall with a 950 ms interval between pulses. For each additional day of embryo age, we increased the voltage by 2 V, while keeping the other parameters unchanged. For P0 mouse, the voltage was 95–100 V. Embryo near the cervical opening was excluded from electroporation. Electroporation was carried out using a pair of 7 mm platinum electrodes (BTX, Tweezertrode 45-0488, Harvard Apparatus) connected to an electroporator (BTX, ECM830). After electroporation, the wound of the pregnant mouse was carefully sutured. Mouse brains were collected and analyzed at various time points.

### Fixation and sectioning of the brain tissue

Postnatal mouse brains were harvested after deep anesthesia and intracardiac perfusion with 4% DEPC-PFA. Embryos were collected from deeply anesthetized pregnant mice, and the brains were carefully dissected out, followed by fixation in 4% DEPC-PFA. The brains were then fixed overnight in 4% PFA at 4 °C, cryoprotected in 30% sucrose for at least 24 h, and embedded in O.C.T. (Sakura Finetek) for freezing. All mouse brains in this study were sectioned coronally at either 20 μm or 50 μm using slicer (LEICA CM1950).

### Immunohistochemistry

Immunohistochemistry was performed as standard protocol^6^. The brain slices were washed three times with tris-buffered saline (TBS) and incubated in 0.5% Triton X-100 for 30 min to permeabilize the membrane. Blocking Buffer (5% donkey serum and 0.5% Triton X-100 in TBS) was incubated at room temperature (RT) for 2 h, followed by incubation with the primary antibody solution at 4 °C overnight. The next day, TBS was used to wash away the primary antibody, and the Alexa Fluor-labeled secondary antibodies were incubated at RT for 2 h, protected from light. Finally, 4′,6-Diamidino-2-phenylindole was used to stain the cell nuclei.

For the detection of biocytin-filled cells in electrophysiological experiment, we employed an alternative method. The recorded slices were fixed in 4% PFA for at least 48 h. After fixation, the slices were washed three times with phosphate-buffered saline (PBS) in a 48-well plate and then incubated in 0.5% Triton X-100 for 30 min. Next, the slices were incubated overnight at 4 °C with streptavidin (1:500, Invitrogen, S11223) solution containing 0.2% Triton X-100. The following day, PBS was used to wash away any excess antibody. Finally, the sections were cover slipped and observed.

The following primary antibodies were used: GFP (Chicken, Aves Labs GFP-1020, 1:3000), tdTomato (Goat, SICGEN Ab8181, 1:1000), ASCL1 (Rabbit, Cosmo Bio SKT01-003, 1:1000), SP8 (Goat, Santa Cruz Biotechnology Sc-104661, 1:500), KI67 (Mouse, BD Pharmingen 556003, 1:1000), OLIG2 (Rabbit, OasisBiofarm OB-PRB066, 1:500), ALDH1L1 (Rabbit, Abcam, ab87117, 1:1000), EGFR (Goat, R&D Systems, AB-355937, 1:1000), GFAP (Rat, Invitrogen, 13-0300, 1:1000), SOX10 (Guinea pig, R&D Systems, AF2864, 1:1000), SOX9 (Rabbit, Abcam, ab185966, 1:2000). HOPX (Rabbit, Proteintech, 11419-1-AP, 1:500), PDGFRA (Rat, BD Pharmingen, 558774, 1:1000), ID3(Rabbit, Biocheck, BCH-4/17-3, 1:3000), S100B (Rabbit, DAKO, Z0311, 1:1000), GS (Rat, oasis biofarm, OB-PRT016, 1:1000), NEUN (Rabbit, Abcam, ab177487, 1:1000), CUX1 (Rabbit, Santa Cruz Biotechnology Sc-13024, 1:5000), MAP2 (Rabbit, Proteintech, 17490-1-AP, 1:1000), beta-ACTIN (Mouse, abclonal, AC004, 1:200), CB (Rabbit, Swant, 1:1000), FOXP1(Rabbit, Abcam, ab314488, 1:1000), PH3 (Rabbit, Millipore 06-570, 1:1000), FOXP2 (Goat, Santa Cruz, sc-21069, 1:500), Cleave Caspase-3 (Rabbit, Cell Signaling, 9661, 1:1000), Tyrosine Hydroxylase (TH; mouse, sc-25269, 1:1000), PSD95(Rabbit, Abcam, 238135, 1:1000).

### Image acquisition and analysis

Stained tissue sections were acquired using the Olympus FV3000 confocal microscope system and the Olympus VS120 automated Slide Scanner. All cell counts were performed either on Z-stack confocal images or images from the Olympus VS120 Automated Slide Scanner with 20× objective magnification. Normally, at least 3 sections per cortex and 3 cortices were analyzed. Cell counts were processed using Adobe Photoshop CC, while Image-Pro Plus (IPP) software was utilized to assess expression levels.

### Two-dimensional (2D) reconstruction

The sections were imaged using a 20× objective on a 30 µm z-axis stack with a VS120 automated slide scanner. Individual complete neurons (filled with dye and exhibited minimal overlap with adjacent cells) and dendritic spines (50~100 μm from the soma) of neurons in the 5^th^(mainly) layers of the prelimbic (PrL) and infralimbic (IL) of the prefrontal cortex (PFC) were reconstructed using the filament function of Imaris 10.1.1 software.

### Sholl analysis

To quantify dendritic arborization complexity, Sholl analysis was performed on reconstructed pyramidal neurons using Fiji with the Sholl Analysis plugin. The analysis commenced at a radius of 1 μm from the soma and extended to 281 μm, with a radial increment of 20 μm. The primary metric quantified was the number of intersections at each concentric circle (the intersection count). The spine density (50~100 μm from the soma) and number of terminal points can be acquired in Imaris data.

### Western blot analysis

Cortical tissue samples were dissected from P0 *Egfr*-conditional knockout (*Egfr*-CKO) and control mice under a stereomicroscope. Proteins were extracted using RIPA lysis buffer (Beyotime, P0013K) supplemented with a protease inhibitor cocktail (Sigma, 11873580001) and a phosphatase inhibitor (Sangon, C500018). A precast 4–20% protein plus gel (Yeasen, 36264ES10) was used to separate equal amounts of protein from different groups at a constant voltage of 100 V for 80 min using the Novex^®^ Blot Module (Invitrogen). Subsequently, protein was transferred to a 0.45 μm nitrocellulose membrane (Invitrogen) by blotting at constant 100 V for 70 min. The membrane was blocked with 5% (w/v) non-fat milk in TBS, then incubated overnight at 4 °C with primary antibodies diluted in blocking buffer (Goat anti-EGFR, R&D Systems, AB-355937; Rabbit anti pERK, Cell Signaling Technology, 4370; Rabbit anti-EPB41L2, Abclonal, A9101; Mouse anti-β-actin, Abclonal, AC004). Following three washes with TBST (TBS containing 0.1% Tween-20), the membrane was incubated for 1 h at room temperature with horseradish peroxidase (HRP)-conjugated secondary antibodies (anti-goat or anti-rabbit IgG (H + L), Beyotime). After washing, the blots were imaged using e-blot biomolecular imager (ebiotrade, Shanghai, China) after incubating with BeyoECL Star (Beyotime, P0018AS) detection solution. Image Pro Plus software was used to quantify bands.

### Co-Immunoprecipitation

Co-Immunoprecipitation was performed using a commercial Immunoprecipitation Kit with Protein A/G Magnetic Beads (Beyotime, P2179S). Briefly, the mouse P0 cortex was lysed in buffer containing 1× phosphatase inhibitor complex I and a protease inhibitor cocktail. The clarified lysate was incubated with 5 μg of rabbit anti‑pERK antibody (Cell Signaling, 4370) for 2 h at RT with gentle rotation. Subsequently, pre‑washed Protein A/G magnetic beads were added to the lysate‑antibody mixture and incubated for an additional 1 h at RT under constant rotation. Finally, the bound immune complex was dissociated from the beads using 1× SDS-PAGE Sample Loading Buffer for subsequent western blot analysis.

### Golgi Stain

The animal brains were stained using the FD Rapid GolgiStain™ kit (FD NeuroTechnologies, INC, PK401). The morphology of layer V pyramidal neurons in PrL and IL cortices was reconstructed and analyzed. Animals were deeply anesthetized, and the brains were quickly removed from the skull to minimize tissue damage. After rinsing with deionized water, the tissue was immersed in the impregnation solution (solution A/B) for 2 weeks at RT in the dark. Impregnation solution was replaced after the first 6 h of immersion or on the next day. Subsequently, the tissue was transferred to Solution C, which was replaced after 6~ 24 h, and kept in the dark for an additional 72~120 h. Afterwards, the brains were sectioned into 100 μm thick slices and allowed to dry naturally overnight at RT. To develop staining according to the protocol, brain slices were washed in distilled water (2 × 4 min) and then submerged in solution D and E for 10 min. The sections were dehydrated through sequential rinses in 50%, 75%, 95%, and 100% ethanol, with each rinse lasting 4 min. The sections were then cleared in xylene three times, with each rinse lasting 4 min. Finally, the slices were cover slipped for observation.

### Assessment of behavior

We conducted multiple experiments to explore whether *Egfr*-CKO mice exhibit behavioral abnormalities. All behavioral experiments utilized 8~11 weeks old mice, with littermate homozygote mice serving as controls. To minimize stress carryover effects, tests were administered in order of increasing aversiveness: Open Field Test-Novel Object Recognition-Y-Maze-Elevated Plus Maze-Rotarod Test-Grip Strength Test-Fear Conditioning-Sucrose Preference Test-Tail Suspension Test-Forced Swim Test. A 7-day interval was maintained between fear conditioning and sucrose preference testing. Only one behavioral test was performed per day. Male and female mice were employed equally in the behavioral tests, except for the social interaction test. The female mice were nulliparous. All experimental groups were composed of offspring from a minimum of 6 independent litters. In a separate set of experiments to investigate the role of *PlxnA2/4*, its expression was knocked down in the embryonic mPFC of CD1 mice via IUE of targeting shRNAs at E13.5. These mice were then subjected to a battery of depression-related behavioral tests, including the sucrose preference, tail suspension, and forced swim tests, at 8~9 weeks of age. In all behavioral tests, the mice were transported to the testing room in their home cages and allowed to acclimate to the environment for at least 30 min before the tests began.

### Open field test

The open field arena was a square chamber (40 × 40 × 40 cm) monitored by an overhead camera connected to an automated tracking system. Mice were gently placed in the center of the box and allowed to explore freely for 10 min. Their movement paths were recorded by the camera, and EthoVision XT 14 software (Noldus, Wageningen, the Netherlands) was used to calculate the total distance traveled and the time spent in the central area. The chamber was cleaned with 75% ethanol after each test to eliminate any residual olfactory cues.

### Tail suspension test

Depression-like behavior was assessed using the tail suspension test. Mice were individually placed in a cubic observation chamber (30.5 × 30.5 × 30.5 cm) equipped with opaque partitions between compartments to prevent visual and physical contact.

Medical tape was applied approximately 1 cm from the tip of the tail, and each mouse was suspended by its tail from a horizontal bar, ensuring the head was positioned about 30 cm above the chamber floor. The experimenter immediately left the room after suspension. Each session was video‑recorded for 6 min using a digital camera (Hikvision). Immobility time, defined as the absence of any active limb or body movement, was manually scored during the final 4 min of the recording. The chamber was cleaned promptly with 75% ethyl ethanol between trials.

### Forced swimming test

The test was conducted in a transparent glass cylinder (height: 25 cm, diameter: 15 cm), filled with the water to a depth of 20 cm. The water temperature was maintained at 22 ± 1 °C. The animal was swiftly placed into the water, and care was taken to ensure that the experimental mice could not see each other. Afterward, the experimenter left the area. The mouse’s behavior in the water was recorded by a camera for 6 min, and the immobility time during the last 4 min was analyzed. Immobility is defined as the mouse floating on the surface with no other activity, except for necessary movements to keep from being submerged. The water was replaced after each test.

### Sucrose preference test

Anhedonia, a core symptom of depression, was assessed using the sucrose preference test. Mice were first habituated to single housing for several days. They were then provided with two identical bottles—one containing 1% (w/v) sucrose solution and the other with plain drinking water—for a 48 h adaptation period, with the bottle positions swapped every 12 h to prevent side-preference bias. After the adaptation phase, the mice were deprived of food and water for 12 h. For the test itself, both bottles were again presented for 24 h, with the positions of the bottles switched every 12 h. Bottles were weighed before and after the test to measure consumption. Sucrose preference was calculated as: [sucrose solution intake (g)] / [total fluid intake (sucrose + water, g)] × 100%.

### Elevated plus maze test

The elevated plus maze was used to assess anxiety responses in rodents. The apparatus, placed in a quiet and dimmed room, consisted of four intersecting arms (35 cm length × 6 cm width) elevated 74.5 cm above the floor. Two opposing arms were enclosed by 20 cm‑high transparent walls (closed arms), while the other two had only 0.8 cm‑high ledges (open arms).

Each mouse was placed in the central zone maze facing an open arm and allowed to explore the equipment freely for 5 min. Behavior was recorded and tracked automatically using EthoVision XT 14 software (Noldus). The time in open arm, entries in open arm and closed arm was analyzed. The maze was cleaned with 75% ethanol and dried between tests to ensure the absence of olfactory cues.

### Y maze test

Spontaneous alternation behavior, reflecting working memory, was assessed using a Y‑maze. The apparatus consisted of three identical gray arms (each 30 × 6 × 10 cm) oriented at 120° angles from a central area. Each mouse was placed at the distal end of one randomly selected arm and allowed to freely explore for 8 min. Behavior was recorded by an overhead camera and analyzed using EthoVision XT 14 software (Noldus). A spontaneous alternation was defined as consecutive entries into all three different arms within a triplet of arm visits (e.g., ABC, BCA). The percentage of spontaneous alternation was calculated as: [(Number of alternations) / (Total arm entries − 2)] × 100. A lower alternation rate suggested impaired short-term memory.

### Accelerating rotarod test

The accelerated rotarod test assessed motor learning and balance in animals by measuring the time mice remain on the rotating rod as its speed increases. We used a rotarod machine (Ugo Basile, 47650) that features both constant and accelerating speed functions, equipped with a trip box magnetic sensor to detect the time of mouse fell. The experiment consisted of a training phase followed by a testing phase, with a 24 h interval in between. During training, the rotarod machine was set to a constant speed of 10 rpm, allowing the mice to habit for 3 min; if they fell before the 3 min were up, they were immediately returned to the rod. The training was repeated three times, with at least a 1 h interval between each session. Testing was conducted 24 h later for a duration of 5 min, with an acceleration range of 5~40 rpm. The time was recorded as soon as a mouse fell off the rod or began to rotate on the rotarod without running.

### The grip strength test

Forelimb muscle strength was assessed using a grip strength meter (YLS-13A, Shangdong Yiyan Technology). Each mouse was allowed to grasp a horizontal metal grid with its forepaws. The mouse was then gently pulled backward by the tail until it released its grip. The peak force exerted immediately before release was automatically recorded by the meter. Each mouse performed three consecutive trials, and the mean of these trials was calculated as the final grip strength score.

### Novel Object Recognition (NOR) test

NOR test utilized the natural exploratory behavior of mice towards new objects to assess their memory capabilities by measuring the duration of exploration of both new and familiar objects. The experimental setup included an open field apparatus and three objects (A, B, and C), with objects A and B being identical. In the familiarization phase, two identical objects (A and B) were placed in the center (ensuring the objects were odorless and firmly fixed in position), with the objects positioned at opposite corners along the diagonal of the box. The mouse was placed at an equal distance from both objects, and its exploration time on each object was recorded. The test lasted for 5 min. The testing phase was usually conducted 2 h after the familiarization phase, where one of the two identical objects was replaced with a novel object (C). Similarly, the mouse was placed equidistant from the objects with its back facing them and allowed to explore for 5 min. The Recognition Index (RI) was then calculated as follows: RI = Time spent exploring the novel object / (Time spent exploring the novel object + Time spent exploring the familiar object).

### Cued and contextual fear conditioning test

Cued and contextual fear conditioning represented a form of associative learning. The test was performed with the Fear Conditioning System (Ugo basile), which was equipped with a stainless-steel rod floor and connected to a shock delivery unit for foot shocks. Our test paradigm was in principle adapted from the recommended example of Any-Maze software, but with several modifications. The Any-Maze software was used to analyze the freezing time of the mice. The training day, the mouse was placed in the chamber to adapt 2 min, then conducted a 12.5 min fear conditioning training. The training involved a 30 s sound stimulus (80 dB) followed by a 2 s shock (0.6 mA). After a 2 min interval, the next cycle was repeated, and the stimulation ended after 5 cycles. The test was conducted the day after the training was completed. First, contextual detection was performed by turning off the shock and recording the mice’s freezing time for 5 min in the same static environment as the day before the test. Two hours later, a cued fear conditioning test was carried out, introducing some background panels to create a new environment and providing a sound cue. The mice were first placed in the cage to freely explore for 2 min, followed by 60 s of sound stimulation, during which the freezing time of the mice was recorded for 120 s after the sound stimulus began. The freezing time was recorded only if it lasted for at least 1 consecutive second. Additionally, locomotor patterns were screened for darting behavior using objective criteria^102^; no such events were identified in any subject.

### Novelty-suppressed feeding test

Mice were food-deprived for 24 h prior to the novelty-suppressed feeding test (NSFT)^103^. Each mouse was placed in the same corner of a novel, brightly lit (50 lux) plastic cage (40 × 40 × 40 cm) that it had not been exposed to previously. A single food pellet was positioned at the center of the cage, and the test lasted for 10 min. The latency to begin eating, defined as the time from placement in the cage until the mouse grasped the pellet with its forepaws and bit it, was recorded.

#### Social interaction/avoidance test (SI)

We employed the SI test to evaluate stress susceptibility in *Egfr*-CKO mice, following previously established protocols^104–106^. Prior to SI testing, experimental mice underwent a 10 min physical interaction session with a male CD-1 aggressor (AGG). The SI test was conducted 24 h after this exposure^106^. Initially, each mouse was placed in an open-field arena (40 cm × 40 cm × 40 cm) equipped with a small wire mesh cage at one side. Their movement was automatically monitored and recorded for 2.5 min using a video tracking system (Noldus, Wageningen, the Netherlands) to assess baseline exploratory behavior and locomotion in the absence of a social target (AGG). After this period, the mouse was temporarily removed, and the arena was cleaned. Subsequently, exploratory behavior was reassessed over 2.5 min in the presence of a novel male CD-1 social target enclosed within the wire cage. The time spent in the interaction zone and corner zones, as well as overall locomotion, were compared between the two conditions. The SI ratio was calculated as the time spent in the interaction zone when the AGG was present divided by the time spent in the same zone when the AGG was absent. Based on this ratio, mice were classified as stress-susceptible (SS) if their SI ratio was below 1.0, and as resilient (RES) if it was above 1.0. Only adult male mice were used in this study.

### Splash test

To assess motivated self-grooming behavior, we conducted the splash test under red light^104^. Briefly, a 10% sucrose solution was sprayed onto the dorsal coat of the mice, and grooming behavior was video-recorded for 5 min. The latency to initiate grooming was subsequently scored by an observer blinded to the experimental groups.

### Single-cell RNA sequencing

Cell Trace Yellow (Life Technologies, #C34567, 0.5 μL of 10 mmol/L) was injected into the lateral ventricle of control, *Egfr*-CKO and *Map2k1/2* CKO embryos. The labeled cortices area was collected on time and dissociated into a single-cell suspension^6^. The labeled activated single-cells were sorted using a BD FACSAriaII (BD Biosciences). According to the manufacturer’s instructions of the Chromium droplet-based sequencing platform, the scRNA-seq library was constructed. After purification and quantification using the Agilent 2100 Bioanalyzer, the cDNA libraries were sequenced on the Illumina HiSeq 4000.

When removed low quality sequences and joints, high quality sequences (clean reads) were acquired, and then Cell Ranger software was used to process those clean reads to acquire quantitative information on gene expression. Cells were filtered out if they had fewer than 200 detected genes, more than 10% of their genes derived from mitochondrial DNA. Genes were excluded in fewer than three cells. The global scale normalization method Log Normalize was used to normalize the raw read counts. Data were scaled, and batch effect correction was applied to mitigate technical and biological biases. Principal component analysis was performed to determine the important principal components. The default parameters of the Seurat package were used to generate cluster markers and UMAP plots. Marker genes among clusters were identified by comparing the cells within each cluster to all other cells.

### RNA-sequencing

PFC tissue encompassing the PrL, IL, and ACC cortices, along with a minimal portion of the primary motor cortex (approximately Bregma 1.98~1.70 mm), was dissected from *Egfr*-CKO mice and same-litter homozygous control mice (*n* = 4 per genotype). Direct-zol RNA Miniprep kit (Zymo, catalog #R2050) was used to isolate total RNA following the manufacturer’s instructions. The quality of RNA was assessed by measuring RNA concentration and purity, as well as evaluating RNA integrity (RIN > 7). After removing rRNA from the sample, purified mRNA was obtained. Following cDNA synthesis, a sample library was constructed and subjected to sequencing analysis. Gene expression levels were reported as fragments per kilobase of exon model per million mapped reads (FPKM). *P*-value < 0.05 grip was defined as differentially expressed genes.

### Primary cortical neural cell culture and hSEMA6a treatment

P0 mice were anesthetized on ice, and the cerebral cortex tissue, with the vascular membrane removed, was collected. The tissue was cut into small pieces with scissors and digested at 37 °C for 30 min. The digestion solution consisted of 5% papain (Worthington Biochemical, LK003178), 1 × DNase I (Roche, DNase Ⅰ,1010415901) dissolved in DMEM medium (DMEM, gibco). The cells were resuspended into a single-cell suspension in DMEM supplemented with 20% fetal bovine serum and seeded at a density of 15 × 10^4^ cells per well in a 24-well plate pre-coated with 0.5 mg/mL poly‑L‑lysine (Sigma) for 30 min, 24 h prior to seeding. To enrich primary neuronal cells, after culturing for 4 h in a 37 °C incubator with 5% CO_2_, the medium was replaced with Neurobasal A (gibco,10888022) culture medium supplemented with 1% B27 (gibco,17504044) supplement and 1× Glutamax (gibco, 35050061). We changed the medium every 2~3 days, depending on the cell growth status. The cells were treated with hSEMA6a protein (MCE, HY-P76057) at a concentration of 200 ng/mL.

### Phosphoproteomics Analysis

Cortical tissue from P0 mice (2 controls and 3 *Egfr*-CKO) was homogenized in lysis buffer. Total protein concentration in the supernatant was determined by a BCA assay, and sample quality was verified by SDS-PAGE prior to downstream processing. For digestion, 300 μg of protein per sample was processed in 100 mM TEAB. Proteins were reduced with 10 mM TCEP at 37 °C for 60 min and alkylated with 40 mM iodoacetamide at RT in the dark for 40 min. Proteins were then precipitated with pre-chilled acetone (6:1 v/v, acetone:sample) at –20 °C for 4 h. After centrifugation, the pellet was redissolved in 100 μL of 100 mM TEAB and digested overnight at 37 °C with sequencing-grade trypsin at a 1:50 (w/w) enzyme-to-substrate ratio. Peptides were vacuum-dried, reconstituted in 0.1% trifluoroacetic acid, and desalted using hydrophilic-lipophilic balance (HLB) columns. After another round of vacuum drying, peptide concentration was determined with a Peptide Quantification Kit (Thermo, Cat. 23275). The peptides were labeled with TMT reagents (ThermoFisher, 90111). For each sample, 100 μg of peptides were labeled with one channel of TMT reagent for 2 h at room temperature. The reaction was quenched with hydroxylamine. All labeled samples were then combined at a 1:1 ratio based on peptide quantity, vacuum-dried, and desalted. Phosphopeptide enrichment was performed by the Majorbio Bio-Pharm Technology Co., Ltd. provider following their optimized protocol. The TMT-labeled peptide pool was redissolved in the proprietary Binding/Wash Buffer and loaded onto pre-equilibrated enrichment microcolumns. The TMT-labeled peptide pool was redissolved in a proprietary binding/wash buffer, loaded onto enrichment columns, and incubated for 30 min. After sequential washes with the binding buffer and LC-MS grade water, bound phosphopeptides were eluted twice with a basic elution buffer. The combined eluates were acidified with 20% trifluoroacetic acid, desalted using StageTips, and vacuum-dried for LC-MS/MS analysis.

Peptides were separated by nanoflow liquid chromatography on a Vanquish Neo system (Thermo Scientific) using a C18 column (75 μm × 25 cm, Thermo Scientific). A 180 min linear gradient was delivered at 300 nL/min with mobile phase A (2% acetonitrile, 0.1% formic acid) and B (80% acetonitrile, 0.1% formic acid) as follows: 0~5% B in 2 min, 5~23% B in 123 min, 23~35% B in 23 min, 35~48% B in 1 min, 48~100% B in 1 min, maintained at 100% B for 6 min, then returned to 0% B in 4 min and re-equilibrated.

MS analysis was conducted on a Q Exactive HF-X mass spectrometer (Thermo Scientific). The instrument was operated in positive ion mode with a DDA method. Full MS scans were acquired over the mass range of (m/z) 350~1300 with a resolution of 70,000. The AGC target was set to 3e6 with a maximum injection time of 20 ms. For each cycle, the top 20 most intense precursor ions were selected for fragmentation via HCD. MS/MS scans were performed at a resolution of 35,000 with an AGC target of 1e5 and a maximum injection time of 50 ms. A dynamic exclusion window of 18 s was applied to prevent repeated sequencing of abundant ions.

The raw MS/MS data were processed using Proteome Discoverer 2.4 (Thermo Scientific) and searched against the UniProt mouse reference proteome (taxonomy ID: 10090). Search parameters included: trypsin digestion (max two missed cleavages); 20 ppm precursor and 0.05 Da fragment mass tolerances. Static modifications were carbamidomethylation (C) and TMTpro labeling (K, N-terminus). Dynamic modifications included oxidation (M), N-terminal acetylation, and phosphorylation (S, T, Y). Peptide identification was filtered at a ≤ 1% FDR using Percolator.

The obtained search results were then subjected to data statistics and bioinformatics analysis. This experiment focused on the analysis of phosphorylated peptides and phosphorylated proteins. Differentially expressed peptides were clustered based on expression patterns using the gplot package in R for further analysis.

### EGFR protein structure prediction

The structure of EGFR protein and truncated EGFR protein were predicted using AlphaFold2(https://colab.research.google.com/github/sokrypton/ColabFold/blob/main/AlphaFold2.ipynb#scrollTo=_sztQyz29DIC).

### VSV-G retrovirus injection

VSV-G retrovirus (titer:1 × 10^8^ TU/mL) was injected in the lateral ventricles of mice at developmental stages ranging from E18 to P3 at a dose of 1 μL per mouse. Separately, P5 and P7 mice underwent stereotaxic surgery for precise intracerebroventricular injection of the same viral preparation. The labeled glia cells were analyzed at P30.

### Mendelian randomization and summary-data-based Mendelian randomization analysis

To investigate the causal relationships involving astrocytes, candidate genes (PLXNA2/4, SEMA6A, EPB41L2, EGFR), and Major Depressive Disorder (MDD), we conducted a two-step analytical framework integrating Summary-data-based Mendelian Randomization (SMR) and standard Mendelian Randomization (MR)^107^.

Data Sources: Human brain *cis*-expression quantitative trait loci (eQTL) summary data, serving as genetic instruments for gene expression, were obtained from the BrainMeta V2 database (http://yanglab.westlake.edu.cn/software/smr). MDD genome-wide association study (GWAS) summary statistics were sourced from the OpenGWAS database (ID: ebi-a-GCST90038650).

MR Analysis: The MR analysis rests on three core assumptions: (i) the selected genetic variants are strongly associated with the exposure; (ii) the instrumental variables are independent of known confounders; and (iii) the instruments influence the outcome (MDD risk) exclusively via the exposure, with no alternative pathways (the exclusion restriction criterion).

For each candidate gene, instrumental variable SNPs associated with expression levels at genome-wide significance (*P* < 5 × 10^−8^) were identified. To ensure independence and mitigate linkage disequilibrium, a clumping procedure was applied (r^2^ < 0.001, window size  = 10,000 kb). Instrument strength was assessed using the F-statistic, with SNPs having F > 10 retained for analysis to avoid weak instrument bias.

Causal estimates were derived as follows: the Wald ratio method was used when a single SNP was eligible, while the inverse-variance weighted meta-analysis under a random-effects model was applied when two or more independent SNPs were available. To assess robustness and potential pleiotropy, complementary methods were employed, including weighted median, MR-Egger regression, simple mode, and weighted mode. The Steiger directionality test was conducted using the directionality_test() function from the ‘TwoSampleMR’ package to verify the assumed direction of causality (exposure → outcome). A significance threshold of *P* < 0.01 was applied, followed by Bonferroni correction for multiple testing (*α* = 0.05/5 candidate proteins). All analyses were conducted in R using the TwoSampleMR (v0.5.6) and MRPRESSO (v1.0) packages.

SMR Analysis: SMR analysis was performed to test whether the MDD GWAS signal at a given locus is mediated by the expression level of a specific gene. The same brain cis-eQTL (BrainMeta v2) and MDD GWAS (OpenGWAS) summary datasets were used, with a filter criterion of *p*-value < 5 × 10⁻³. SMR analysis was conducted with smr-1.3.1 software, with significance thresholds set at p_SMR < 0.05, p_HEIDI > 0.05, and nsnp_HEIDI > 3.

### Cell-cell communication

CellChat was used to analyze intercellular communication between distinct cell types. The standard CellChat workflow was followed to process the data^108^. First, cells were analyzed by transcriptomics to identify over-expressed genes per cell group. Then, CellChat models communication probabilities and identifies significant communications. Finally, communication networks between astrocytes and neurons were visualized. The line thickness in CellChat networks reflects normalized communication probability, calculated as (ligand expression×receptor expression)/cell count^108^. This per-capita normalization ensures thickness represents signaling strength per cell rather than population size.

### Stereotactic injection

To investigate synaptic connectivity and network dynamics in the PFC, stereotaxic injections of AAV2/8-hSyn-hChR2(H134R)-mCherry-ER2-WPRE-pA were performed into the prelimbic region of the PFC of ~P30 mice under isoflurane anesthesia (coordinates relative to bregma: AP + 1.95 mm, ML ± 0.25 mm, DV − 1.58 mm). Following a one-month incubation period to allow robust transgene expression, acute coronal brain slices (300 μm) containing both the injected and contralateral PFC were prepared.

### Optogenetics experiments

In mice injected with optogenetic viruses, to validate viral expression, we recorded from mCherry-expressing pyramidal neurons near the injection site. These neurons were reliably activated to fire action potentials upon illumination with 470 nm blue light pulses delivered through a 60× objective, confirming successful viral transduction and functional ChR2 expression.

To assess synaptic connectivity, whole-cell patch-clamp recordings were performed from Layer 5 neurons in the PFC contralateral to the injection site. ChR2-expressing axonal terminals were optically stimulated with 470-nm light pulses (1 ms duration) via the 60× objective. Excitatory postsynaptic current (EPSC) was recorded under voltage-clamp at a holding potential of −70 mV to evaluate the strength of the transcallosal synaptic connections. A detailed input-output relationship was characterized by applying light stimuli at varying intensities (16%, 32%, 64%, 80%, and 100% of maximum laser power) and across a range of frequencies (5, 10, 20, and 40 Hz) to systematically probe the synaptic connectivity and network dynamics.

### Long Term Potential (LTP)

To record LTP, a concentric bipolar stimulating electrode was positioned in Layer 2/3 of the mouse medium PFC. Whole-cell patch-clamp recordings were obtained from Layer 5 pyramidal neurons expressing green fluorescent protein under voltage-clamp mode to monitor changes in EPSC.

A baseline synaptic response was established by delivering single stimuli at 20-second intervals for 5 min. Subsequently, high-frequency stimulation (HFS) was applied, consisting of 3 to 5 trains of 100 Hz stimuli (500 ms per train) delivered at 10-second intervals. Following the HFS protocol, synaptic responses were monitored for at least 25 min by continuing to deliver single stimuli every 20 s.

To isolate excitatory synaptic transmission, the GABAA receptor antagonist bicuculline was added to the artificial cerebrospinal fluid (ACSF) throughout the experiment to block inhibitory postsynaptic potentials. For the purpose of maintaining cell viability over long-term recordings, all electrophysiological data were acquired at 25 °C.

Neurons were voltage-clamped at a holding potential of −70 mV. The holding current was adjusted as necessary to keep the membrane potential within a ± 2 mV deviation from the target holding potential of −70 mV.

### Cortical slice preparation and in vitro electrophysiology

The mice, approximately 60 days old (P60), were anesthetized with isoflurane and transcardially perfused with an ice-cold, oxygenated cutting solution (95% O₂, 5% CO₂) at pH 7.3~7.4 and an osmolarity of 290-300 mOsm. The solution contained the following (in mM): 110 chlorine-Cl, 10 Na ascorbate, 3.1 Na pyruvate, 2.5 KCl, 1.25NaH_2_PO_4_, 4MgSO_4_, 1CaCl_2_, 26NAHCO_3_, 11 D-glucose. Coronal slices were sectioned at a thickness of 300 µm using a vibratome (Leica VT1200S). The slices were incubated in a chamber filled with cutting solution at 34 °C for 30 min. After this, the slices were transferred to working ACSF (pH 7.35~7.4, 290–300 mOsm) containing the following components (in mM): 124NaCl, 2.5KCl, 26NaHCO_3_, 1.25NaH_2_PO_4_, 11D-glucose, 2MgSO_4_, 2CaCl_2_. They were then incubated at RT for 1 h. Finally, the slices were transferred to the recording chamber of a vertical microscope (Olympus BW51) filled with ACSF (continuously perfused and keeping in 34~35 °C). Whole-cell patch clamp was performed in the deep layer of the mPFC with the subcortical white matter and the corpus callosum as primary landmark.

A Sutter P1000 puller was used to pull patch pipettes from borosilicate glass capillaries with filament (1.5 mm outer diameter and 0.86 mm inner diameter, Sutter BF150-86-10). The resulting pipettes exhibited a resistance of 5-8 MΩ when filled with internal solution and measured in ACSF. The following solution (in mM) was used to fill the patch pipettes for recording cell properties: 120 K-gluconate, 16 KCl, 2 MgCl_2_, 10 HEPES, 0.2 EGTA, 2.5 MgATP, 0.5 Na3GTP, 10 Na-phosphocreatine, pH 7.35, 280-310 mOsm and biocytin (0.2%-0.5%, Invitrogen, B1592). In voltage-clamp mode, the membrane potential was maintained at −70 mV, whereas in current-clamp mode, no holding current was applied; however, series resistance ( < 30 MΩ) was compensated. Signals were recorded at 20 kHz and filtered at 3 kHz (Digidata 1550 A, Molecular Devices). The properties of the single action potential (AP) were measured from the first spike fired at rheobase. The threshold of an action potential was defined as the voltage at which the derivative of the membrane potential (dV/dt) reached 25 mV/ms. Rheobase was determined by applying increasing depolarization current steps of 10 ~ 50 pA (1000 ms).

For recording miniature excitatory currents (mEPSC), the patch pipettes were filled with the following solution (in mM): 120 Cs-methanesulfonate, 15 CsCl, 10 HEPES, 2 Mg-ATP, 0.3 Na3-GTP, 1 EGTA, 10 Na2-phosphocreatine, and 2.5 QX-314, pH 7.35, 280–310 mOsm. The same internal solution was used, with the addition of the following channel blockers added (in μM): 1 tetrodotoxin, and 10 bicuculline. During recording, cells were voltage-clamped at −70 mV. Signals were recorded at 10 kHz and filtered at 2 kHz. Data were analyzed with Multiclamp 700B, MiniAnalysis 6.0 and pClamp 10.7.

In terms of optogenetic experiments, the patch pipettes were filled with an internal solution containing (in mM): 120 K-gluconate, 16 KCl, 2 MgCl₂, 10 HEPES, 0.2 EGTA, 2.5 MgATP, 0.5 Na₃GTP, 10 Na-phosphocreatine, and 0.2–0.5% biocytin (pH 7.35, 280–310 mOsm). Throughout the recordings, the chamber was continuously perfused with oxygenated ACSF maintained at 34–35 °C. Neurons were voltage-clamped at −70 mV. Series resistance was compensated and monitored below 30 MΩ. Signals were digitized at 20 kHz and filtered at 3 kHz using a Digidata 1550 A system.

**Table:** 

Software and Algorithms		
Clampex 10.7	Molecular Devices	https://www.moleculardevices.com/
MultiClamp 700B Commander	Molecular Devices	https://www.moleculardevices.com/
MiniAnalysis 6.0	Synaptosoft	https://www.synaptics.com/cn/homepage

### Extracellular ATP measurement

We detected the PFC extracellular ATP using an ATP assay kit (Beyotime, S0026)^87^. Briefly, the PFC slices were incubated in ice-cold oxygenated ACSF for 12 min. The ACSF was then collected for ATP measurement. Protein levels were normalized to the total protein content of each sample for quantification.

The ectonucleotidase inhibitor 6-*N*,*N*-diethyl-β-γ-dibromomethylene-D-adenosine-5-triphosphate FPL 67156 (ARL 67156 trisodium salt) was added to the ACSF to inhibit ATP hydrolysis. Luminescence was measured with a microplate reader (Tecan). ATP levels in the samples were calculated based on the calibration curve generated from ATP standards.

### Statistical analysis

We performed statistical analysis with GraphPad Prism 9.0. Data were presented as mean values, mean ± SD or violin plots, and a two-tailed Student’s *t*-test was used to assess differences between two groups. Welch’s correction is applied for groups with unequal variances. The unit of study was defined according to the experimental paradigm. For single-cell assays (e.g., electrophysiology, imaging, and staining quantification), *n* represents the number of cells, all obtained from at least three independent biological replicates (distinct mice for in vivo; independently prepared cultures from different donors for in vitro). For biochemical measurements, *n* represents the number of independent biological replicates (mice or culture preparations), with each condition tested in at least three replicates. For behavioral assays, *n* represents the number of individual mice, with each group consisting of at least 6 mice. Detailed statistical information and source data are provided in the Suppl. materials.

### Reporting summary

Further information on research design is available in the Nature Portfolio Reporting Summary linked to this article.

## Source data

Source Data

## Data Availability

The Single-cell RNA-seq and bulk RNA-seq that support the findings of this study are available at Gene Expression Omnibus under accession number GSE282783. Proteome data are available at iPROX (PXD058068, https://www.iprox.cn//page/project.html?id=IPX0010286000). Human^109^ (fetal to adult) and mouse^110,111^ PFC Single-cell RNA-sequencing data (postnatal and adult) are from GEO datasets. No custom code was used. Source data are provided with Source Data file. All other data are available from the corresponding authors upon request. Source data are provided with this paper.

## Suppl. information

Suppl. Information
Description of Additional Suppl. Files
Suppl. Data 1
Suppl. Data 2
Suppl. Data 3
Suppl. Data 4
Suppl. Data 5
Suppl. Data 6
Reporting Summary
Transparent Peer Review file
