# Supplementary material for: Delayed astrocyte development impairs Sema6a-Plxna2/4-mediated astrocyte-neuron crosstalk and causes depressive-like behavior
Source: Nat Commun. 2026 Jul 18;17:8800. doi: 10.1038/s41467-026-75697-z (PMC13494039; doi:10.1038/s41467-026-75697-z)
Supplement: Supplementary file 1 [file 41467_2026_75697_MOESM10_ESM.pdf]

nature portfolio

Peer Review File

Delayed Astrocyte Development Impairs Sema6a-Plxna2/4-Mediated Astrocyte-Neuron Crosstalk and Causes Depressive-like Behavior

Corresponding Author: Dr Guoping Liu

This file contains all reviewer reports in order by version, followed by all author rebuttals in order by version.

Version 0:

Reviewer comments:

Reviewer #1

(Remarks to the Author)
In, "Delayed crosstalk between astrocytes and neurons leads to depressive-like behaviors," Jiang and Qi et al., investigate early developmental interactions between astrocytes and neurons and how may contribute to adult pathology. First, the authors show that prior to P7, astrocytes display close connections with pyramidal neurons, which is reduced at P21 and into adulthood. In humans, pyramidal neurons increase their interactions with astrocytes in the neonatal period, strengthening in infancy and adolescence. Disruption of Egfr in the cortex during an early critical period reduces the number of astrocytes in the early post-natal period and this reduces interactions between pyramidal neurons and astrocytes. This occurs because Egfr functional KO in the cortex blunts migration of astrocyte progenitor cells through MAPK, and partially through function of EPB41L2. Egfr cortical KO altered dendritic arborization but not dendritic spine numbers in the PFC in adolescence. Egfr cKO altered neuronal signaling as well as some coping and anhedonia behaviours. SEMA6-Plxn4a was highlighted as a signaling pathway between astrocytes and neurons that contributes to the changes in dendritic arborization following Egfr2 cKO. Finally, utilizing currently available GWAS data demonstrated that astrocyte associated genes were associated with MDD.

My main concern is that the authors keep saying disruption of astrocyte-neuron crosstalk, without showing a lot of specific interactions (and very few outside of RNAseq interaction analysis, which is only utilized for the initial experiments, but not the subsequent follow up). A key experiment here would be to perform this interaction analysis as well in the MAP2K KO and show that it is attenuated in the Fgf18 and EPB41L2 overexpression studies. Further, the specific ligand pairs (SEMA6) could be interrupted during this early time period to show that specific astrocyte-neuron crosstalk is important in mediating these behaviours, not just dendritic arborization (as alterations in signaling in the cortex was not necessarily linked mechanistically to behaviour).

Additionally, there are some areas where clarifications could be implemented:

Results and Rationale Clarifications
1. Figure S1A – how did the authors confirm these were astrocytes?
2. The authors assert RGC give rise to astrocytes in a lateral to medial pattern, but there is staining in the medial regions with those injected at E18? Perhaps instead, there is more broad patterning from earlier time points. Same for SF3 EGFR KO.
3. Fig 1 title should be updated, the figure doesn't show that embryonic EGFR KO leads to delayed crosstalk. The authors should include aspects of SF4e in Figure 1 and instead say that Egfr cKO leads to altered astrocyte organization. What time point is FS4e from? Are there just less connections because there are less astrocytes?
4. How Fgfr 1 and 3 and Fgf18 relate to MAPK signaling and then both to Egfr could be better described in the text.
5. While EPB41L2 certainly attenuates the effects of Egfr KO, it doesn't fully rescue it, so some toning down of language here is warranted.
6. Golgi analysis performed at P40 and biocytin performed at P50. Do the authors think there is potentially a difference there? Were both analyzed from Layer V? Were the dendrites analyzed for spines ones that were from distances from the soma where there were differences observed (50-100 um apx?) or further out (200 onward)?
7. Please provide citations for the neuronal alterations and psychiatric disorders. Where in the PFC were these analyzed?

Do the authors mean the pre- or infralimbic or cingulate cortices (medial PFC) specifically or are these from anywhere from those regions to the M1 and S1 cortices? This is especially important as the prelimbic and infralimbic cortices can have opposing actions on behaviour.
8. Since the authors also utilized females, did they analyze darting in the fear conditioning responses?
9. Yes, the FST, TST and SPT are often utilized to assess "depressive-like behaviours" (or rather anti-depressant therapeutic screening). However, to consider being more specific and discuss that these leads to a reduction of active coping or a switch to passive coping in the FST and TST and an increase in anhedonia in SPT.
10. Why do the authors think there was an increase in open arm entries in the EPM? This does not necessarily indicate a locomotor effect. And the mice do not spend more time there, so what is happening?
11. Did the authors consider assessing any additional behaviours, risk perception, repetitive etc, in their behavioural tasks?
12. Why would Sema6a-Plxna2/4 expression be elevated in the Egfr cKO, if this pathway is proposed to be part of the disrupted signaling between astrocytes and neurons?
13. Saying that "EPB41L2, PLXNA2, and PLXNA4 has a direct and significant causal effect on MDD" (Line 299), is an overstatement. Consider using associated with MDD, as is in the figure legend.

Methods Clarifications
14. Please confirm how many animals per litters were used for each endpoint. In general, no more than 2 mice per sex should be included unless combining for a single "n". Please confirm if litters were considered. Were dams nulliparous prior to experiment or no? Just to also confirm, were males and females equally utilized throughout all experiments?
15. At what age were mice considered adults? 7-8 week old is still late adolescence to very early adulthood. Were they P60 or 7-8 weeks old (P49-P56) for behaviour?
16. Please indicate the anatomical coordinates and sub-regions included in PFC for RNAseq studies (and the rest of the studies). Unclear the differentiation of regions in Figure S1b, there is potentially some overlap in those regions divided out. None of them would technically include the IL or PL, correct? But later in the manuscript, these are proposed as important regions for mediating these effects.
17. Missing many details about the many of the specific overexpression (when during development or sequences) in the text.
18. The authors should consider a nested t-test for their ephys data to use animal as the "n."
19. What was the order of the behavioural tasks? Were any performed on the same day?
20. Statistics should be reported (i.e., t- and p-values should be reported in a supplemental table).

Reviewer #3

(Remarks to the Author)
I co-reviewed this manuscript with one of the reviewers who provided the listed reports. This is part of the Nature Communications initiative to facilitate training in peer review and to provide appropriate recognition for Early Career Researchers who co-review manuscripts.

Reviewer #4

(Remarks to the Author)
This manuscript presents a comprehensive investigation into the role of delayed astrocyte-neuron crosstalk on neurodevelopment and its behavioral consequences using an Egfr knockout mouse model. The study effectively elucidates the critical importance of astrocyte-neuron interactions during the developmental critical period, highlighting the molecular mechanisms underlying impaired crosstalk and its impact on neuronal morphology, excitability, and depressive-like behaviors. The findings offer valuable insights into the potential link between astrocyte dysfunction and major depressive disorder (MDD), suggesting novel therapeutic targets for psychiatric disorders.
The manuscript is well-structured, with clear articulation of hypotheses, detailed experimental design, and thorough data analysis. The integration of single-cell RNA sequencing, electrophysiology, and behavioral assays provides a robust and multidimensional approach to understanding the complex interplay between astrocytes and neurons. However, some points require clarification and elaboration.

Major Comments:
Additional investigation into the compensatory mechanisms would strengthen the paper. The authors show that neurons exhibit reduced excitability and dendritic complexity in adulthood, but it remains unclear whether these changes are permanent or potentially reversible. This has important implications for the fundamental roles of neuron-astrocyte communication that should be addressed.

The manuscript presents compelling evidence linking the impaired EGFR-pERK-EPB41L2 axis to delayed astrocyte migration. However, the downstream effects on neuronal circuits are not fully elucidated. Additional experiments that validate the functional consequences of this impaired signaling on synaptic connectivity and network dynamics would strengthen the findings.

The role of the Sema6a-Plxna2/4 receptor pair is convincingly shown to influence dendritic complexity. Yet, its impact on synaptic plasticity and long-term potentiation (LTP) should be explored to provide a more comprehensive understanding of the behavioral phenotypes observed.

While the depressive-like behaviors in Egfr knockout mice are well-documented, it would be beneficial to include additional behavioral tests to further substantiate the link to MDD, such as the social interaction test or novelty-suppressed feeding test.

This would provide a more comprehensive behavioral profile and strengthen the translational relevance of the findings.
The specificity of the observed behavioral effects on depression-like phenotypes needs more thorough investigation. Additional behavioral tests examining other domains (anxiety, cognition, social behavior) would help establish the specificity of the phenotype.
The authors should discuss the potential influence of other neural circuits (e.g., serotonergic or dopaminergic pathways) on the observed depressive-like behaviors, as these pathways are also implicated in MDD.

The Mendelian randomization analysis linking EPB41L2, PLXNA2, and PLXNA4 to MDD is compelling. However, the manuscript would benefit from a more detailed discussion of the potential clinical implications, including how these findings could inform therapeutic strategies for MDD. Additionally, a comparative analysis of the temporal dynamics of astrocyte-neuron crosstalk between mice and humans would enhance the translational impact of the study.

Minor Comments:
Expand on the discussion regarding the relevance of the EGFR-pERK-EPB41L2 axis in other neuropsychiatric conditions, considering its role in synaptic regulation.

Some of the figure panels would benefit from additional quantification, particularly in Figure 2, where some morphological changes are shown qualitatively.

The authors should provide more detail about the age-specific differences in astrocyte-neuron communication patterns in the human data presented in Figure 1d.

Statistical analyses should be more clearly described in some figure legends, particularly regarding the number of biological replicates used (For example, Fig. 2a, 4c, and 4d).

Additional controls for the specificity of the EGFR knockout effects on astrocytes versus other cell types would be valuable.

Fig. 2a, b: The criteria for morphological analysis of cells should be more explicitly defined in the Methods section.

Reviewer #5

(Remarks to the Author)
The authors have investigated the consequences of conditional Egfr deletion. They find that this manipulation affects the maturation of astrocytes, cell marker expression, and cellular morphology. They also reveal that it leads to altered action potential firing and changes of synaptic transmission and neuronal morphology. The involvement of ERK-related signaling pathways and semaphorin 6A is investigated as well. There are concerns regarding, for instance, some experimental approaches and the presentation of the results.

1) Many experiments use the conditional deletion of Egfr using a commercially provided mouse line in which exon 2 of Egfr is 'floxed' and which was not fully characterized, crossed with a hGFAP-cre mouse line. It is unclear where the latter came from because a review is cited instead of an original paper characterizing the mouse line. This is problematic because it is well known that various cell types transiently show GFAP promoter activity and that, therefore, cre expression and cre-dependent recombination are not restricted to a single cell type (Casper and McCarthy, 2006). Without a careful characterization of the cell types from which Egfr is deleted and their respective percentages the data obtained with these animals is difficult to interpret.

2) The manuscript is difficult to read and understand because terms are not well explained. It seems that 'crosstalk' refers to all potential interactions that astrocytes and neurons are capable of, which includes everything from the astrocytic role in neuronal maturation and synaptogenesis to their role as signaling partners defining synaptic function, plasticity and complex behaviors. This seems too broad and unspecific to be useful given the current knowledge of the many ways that astrocytes contribute to brain function. The same applies to the term 'communication', which is also used in a very broad fashion. Inferring such communication from single-cell RNA sequencing data (e.g. lines 84-85) seems to ignore most of the complexity of cell-to-cell communication in the brain.

3) The data is incompletely described. The number of experiments, mice, samples and cells are mostly missing. There is insufficient information given about statistical tests and their results in the manuscript. There are incompletely labeled figures and unexplained abbreviations. For instance, in the experiments with Sema6a expression in Fig. 4d, it is unclear how often these experiments were performed, whether there are significant differences, and the x-axis label is missing. Consequently, it is often not possible to follow the conclusions presented by the authors.

Casper KB, McCarthy KD (2006) GFAP-positive progenitor cells produce neurons and oligodendrocytes throughout the CNS. Mol Cell Neurosci 31:676–684.

Version 1:

Reviewer comments:

Reviewer #1

(Remarks to the Author)
Overall the authors have addressed the reviewers' concerns. However, in a few instances, inclusions of the points made to reviewers should be included in the manuscript and aren't (in general, for the future, recommend indicating where in the manuscript different aspects of the revisions are included). I have included a non-exhaustive list, but encourage the authors to double check the reviewers' comments and make sure that the responses, when able to be, are updated in the manuscript text.

Reviewer 1
Point 1. Thank you to the authors for their summary of crosstalk. However, as multiple reviewers mentioned, there's a conflation of cell-cell interactions broadly versus one specific ligand-receptor interaction. Encourage the authors to be specific in their title and their conclusions.
Point 2, are the violin plots in the updated manuscript?
Point 7 about references was meant to indicate to include them in the text.
Points 14, 15, 19. Please make sure this information is included in the text.

In addition to those updates, I think the authors could include a bit more of a link between their original findings, which are outside of the regions they cite as being important for behaviours (Reviewer 1 Point 16). Can they include any IHC of the Pre- or Infra-limbic cortices with regards the altered astrocyte organization (Figure 2,3, Supplemental Figure 1-3, 5, 8, etc)? If not, this should be discussed in the manuscript.

Missing references for sentences on lines:
60-67
70-74
125-126
133-135
207-210
257-259
297-302
409-413
448-462

Reviewer #3

(Remarks to the Author)
I co-reviewed this manuscript with one of the reviewers who provided the listed reports. This is part of the Nature Communications initiative to facilitate training in peer review and to provide appropriate recognition for Early Career Researchers who co-review manuscripts.

Reviewer #4

(Remarks to the Author)
This revision presents a solid and comprehensive mechanistic framework linking astrocyte developmental timing with neuronal circuit maturation and mood regulation. The addition of long-term behavioral and electrophysiological validation greatly enhances the manuscript's impact and suitability for publication.

Reviewer #5

(Remarks to the Author)
The authors have not convincingly addressed my previous comments. Regarding the first comment, for example, they now cite the paper describing the hGFAP-cre mouse line (Zhuo et al., 2001). This study demonstrates that cre expression in this line begins around embryonic day 13 in cells that later give rise to neurons, astrocytes, and other cell types. Both this and other work (Casper and McCarthy, 2006; Zhuo et al., 2001) further show that cre expression is heterogeneous, with reporter activity observed only in subpopulations of certain cell types. From this relatively poorly defined population of cells, Egfr is deleted before and during their differentiation. It is also deleted from cells within the postnatal neurogenic niches that exhibit GFAP promoter activity. It remains unclear to this reviewer how solid conclusions can be drawn from this rather undefined experimental manipulation.

Version 2:

Reviewer comments:

Reviewer #1

(Remarks to the Author)
The revision has addressed all our concerns

Reviewer #3

(Remarks to the Author)
I co-reviewed this manuscript with one of the reviewers who provided the listed reports. This is part of the Nature Communications initiative to facilitate training in peer review and to provide appropriate recognition for Early Career Researchers who co-review manuscripts.

Open Access This Peer Review File is licensed under a Creative Commons Attribution 4.0 International License, which permits use, sharing, adaptation, distribution and reproduction in any medium or format, as long as you give appropriate credit to the original author(s) and the source, provide a link to the Creative Commons license, and indicate if changes were made.
In cases where reviewers are anonymous, credit should be given to 'Anonymous Referee' and the source.
The images or other third party material in this Peer Review File are included in the article's Creative Commons license, unless indicated otherwise in a credit line to the material. If material is not included in the article's Creative Commons license and your intended use is not permitted by statutory regulation or exceeds the permitted use, you will need to obtain permission directly from the copyright holder.
To view a copy of this license, visit https://creativecommons.org/licenses/by/4.0/

Responses to Reviewers:

Reviewer #1 (Remarks to the Author)

We are extremely grateful for your constructive suggestions, which have helped substantiate the conclusions. We have now carefully addressed all of your suggestions, either by providing clarification or by performing new experiments. Point-by-point responses to your comments are given below, with your comments given verbatim in black text, and our responses in purple:

In, "Delayed crosstalk between astrocytes and neurons leads to depressive-like behaviors," Jiang and Qi et al., investigate early developmental interactions between astrocytes and neurons and how may contribute to adult pathology. First, the authors show that prior to P7, astrocytes display close connections with pyramidal neurons, which is reduced at P21 and into adulthood. In humans, pyramidal neurons increase their interactions with astrocytes in the neonatal period, strengthening in infancy and adolescence. Disruption of Egfr in the cortex during an early critical period reduces the number of astrocytes in the early post-natal period and this reduces interactions between pyramidal neurons and astrocytes. This occurs because Egfr functional KO in the cortex blunts migration of astrocyte progenitor cells through MAPK, and partially through function of EPB41L2. Egfr cortical KO altered dendritic arborization but not dendritic spine numbers in the PFC in adolescence. Egfr cKO altered neuronal signaling as well as some coping and anhedonia behaviours. SEMA6-Plxn4a was highlighted as a signaling pathway between astrocytes and neurons that contributes to the changes in dendritic arborization following Egfr2 cKO. Finally, utilizing currently available GWAS data demonstrated that astrocyte associated genes were associated with MDD.

We thank you for your insightful comments and positive evaluations.

1. My main concern is that the authors keep saying disruption of astrocyte-neuron crosstalk, without showing a lot of specific interactions (and very few outside of RNAseq interaction analysis, which is only utilized for the initial experiments, but not the subsequent follow up).

Cell-cell interactions (CCIs) constitute the fundamental basis of multicellular life, enabling cellular populations to coordinate and execute complex biological functions[1-3]. Specifically, CCIs involve processes wherein cells activate signaling pathways in neighboring cells through specific molecules—such as ligands, receptors, and functional proteins—as well as membrane-associated structures like the extracellular matrix, membrane protrusions, and extracellular vesicles. These interactions regulate gene expression and drive corresponding cellular functions. Among various CCI mechanisms, ligand-receptor interactions (LRIs) represent the predominant mode of regulation. In LRIs, a ligand secreted by one cell binds to its

cognate receptor on another, initiating intercellular signal transduction[1]. This form of LRI-mediated communication, also referred to as cell-cell communication (CCC), plays a pivotal role in both physiological and pathological processes. For instance, during neural development, microglia promote the proliferation of GABAergic neurons by secreting IGF1, which acts on neuronal IGF receptors (IGFR)[2]. Similarly, cortical astrocytes regulate the formation of synapse-specific connections through neuronal Sonic hedgehog (Shh) signaling via the Ptch1 receptor[3]. Furthermore, RABID-seq has identified Sema4D-PlexinB1, Sema4D-PlexinB2, and Ephrin-B3–EphB3 as key ligand-receptor pairs mediating microglia-astrocyte interactions[4]. These interactions not only contribute to the pathogenesis of central nervous system disorders but also represent promising therapeutic targets for neurodegenerative diseases. In summary, CCIs exert precise and extensive control over neurodevelopment, inflammatory modulation, synaptic plasticity, and emotional behaviors through intricate ligand-receptor signaling networks in both health and disease.

In our study, we propose that Sema6a–Plxna2/4 ligand-receptor pairs play critical roles in the development of pyramidal neurons. In accordance with your and the other reviewers' insightful suggestions, we are conducting additional experiments to further validate the functions of this ligand-receptor pair (please refer to the following responses and the manuscript).

References :

1 Zhou, X. et al. Circuit Design Features of a Stable Two-Cell System. Cell 172, 744-757.e717 (2018). https://doi.org/10.1016/j.cell.2018.01.015

2 Rouault, H. & Hakim, V. Different cell fates from cell-cell interactions: core architectures of two-cell bistable networks. Biophys J 102, 417-426 (2012). https://doi.org/10.1016/j.bpj.2011.11.4022

3 Bonnans, C., Chou, J. & Werb, Z. Remodelling the extracellular matrix in development and disease. Nat Rev Mol Cell Biol 15, 786-801 (2014). https://doi.org/10.1038/nrm3904

4 Armingol, E., Baghdassarian, H. M. & Lewis, N. E. The diversification of methods for studying cell-cell interactions and communication. Nat Rev Genet 25, 381-400 (2024). https://doi.org/10.1038/s41576-023-00685-8

5 Yu, D. et al. Microglia regulate GABAergic neurogenesis in prenatal human brain through IGF1. Nature (2025). https://doi.org/10.1038/s41586-025-09362-8

6 Xie, Y. et al. Astrocyte-neuron crosstalk through Hedgehog signaling mediates cortical synapse development. Cell Rep 38, 110416 (2022). https://doi.org/10.1016/j.celrep.2022.110416

7 Clark, I. C. et al. Barcoded viral tracing of single-cell interactions in central nervous system inflammation. Science 372 (2021). https://doi.org/10.1126/science.abf1230

2. A key experiment here would be to perform this interaction analysis as well in the MAP2K KO and show that it is attenuated in the Fgf18 and EPB41L2 overexpression studies.

We thank the reviewer for their insightful suggestions. In response, we performed new single-cell RNA sequencing on postnatal day 2 Map2K1/2 KO cortex and analyzed neuron-astrocyte interactions (shown below, Fig S7e, f). This analysis confirmed a slight reduction in these interactions in the KO cortex. Since our study shows that Egfr knockout impairs astrocyte migration, thereby preventing them from contacting excitatory neurons, we hypothesized that the most impaired interactions are likely mediated by direct-contact ligand-receptor pairs. Subsequent analysis identified the SEMA6A-PLXNA2/4 pair as pivotal. Interestingly, the expression of these genes did not change significantly in either the Egfr KO or Map2k1/2 KO cortex (shown below). Furthermore, the early postnatal lethality of Map2k1/2 KO mice precluded a direct analysis of the neuronal morphology and function rescued by Fgf18 or EPB41L2. Therefore, as an alternative approach, we assessed the rescue effects of Fgf18 and EPB41L2 by measuring the migration distance of EGFR-positive cells.

Fgf18 overexpression can rescue the migration and morphological phenotype of Map2k1/2 KO mice. (shown blew). As in the Egfr KO cortex, EPB41L2 can partial rescue the phenotypes (shown blew).

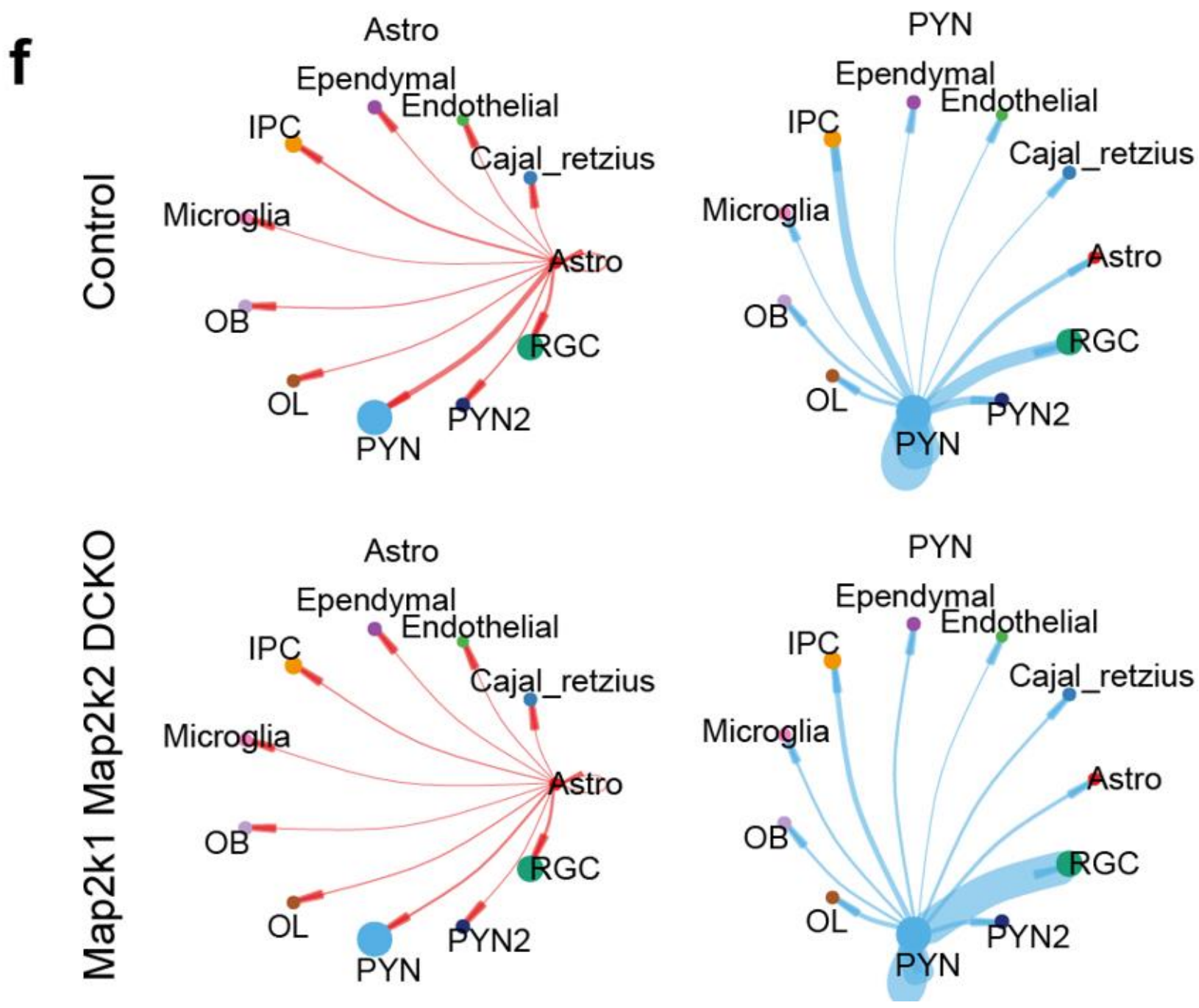

Analysis of astrocyte-neuron communication in the P2 Map2k1/2 CKO and control cortex.

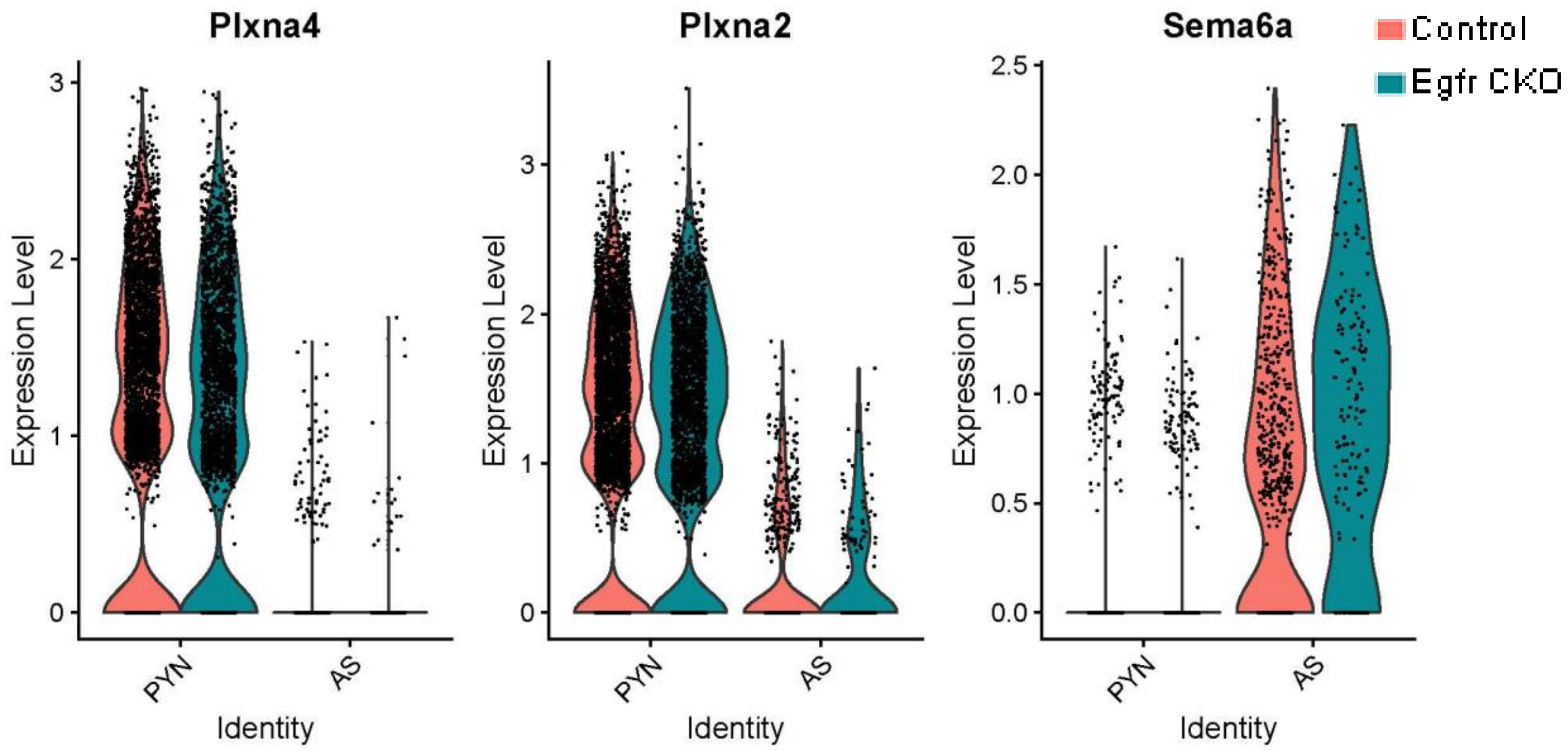

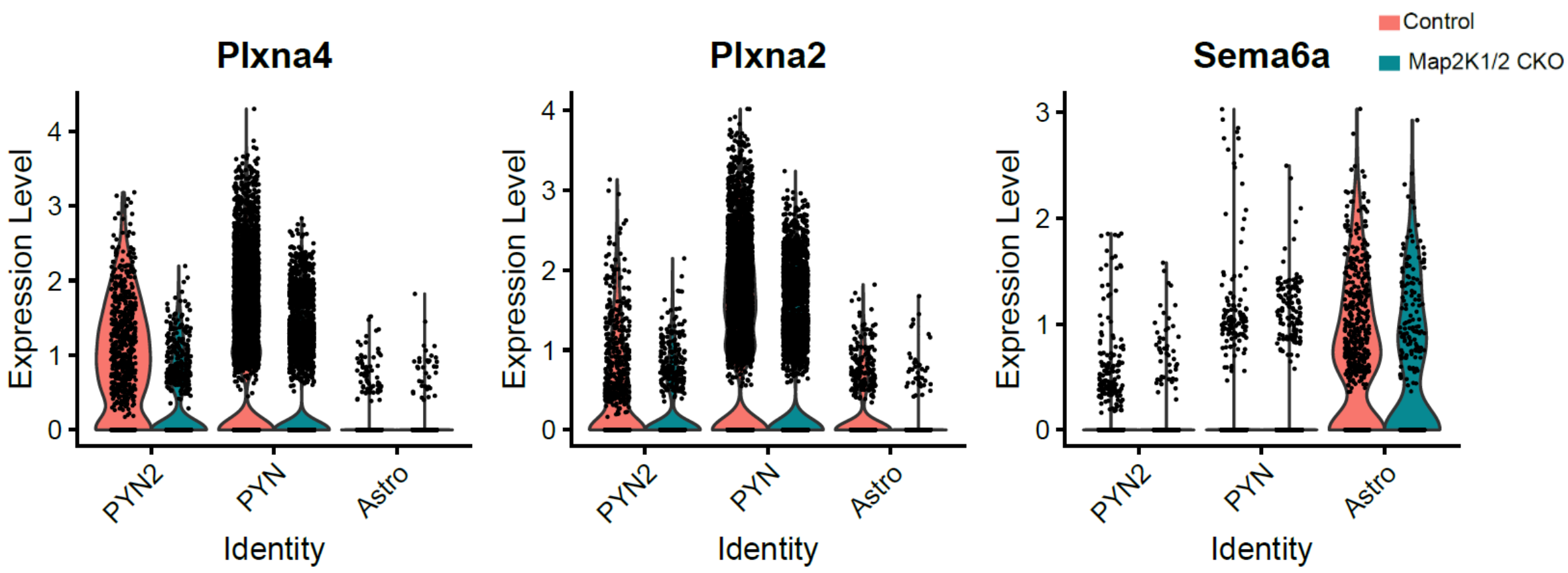

Violin plot shows the expression of Sema6a-Plxna2/4 recpetor pair in the Egfr CKO and Map2k1/2 dCKO cortex.

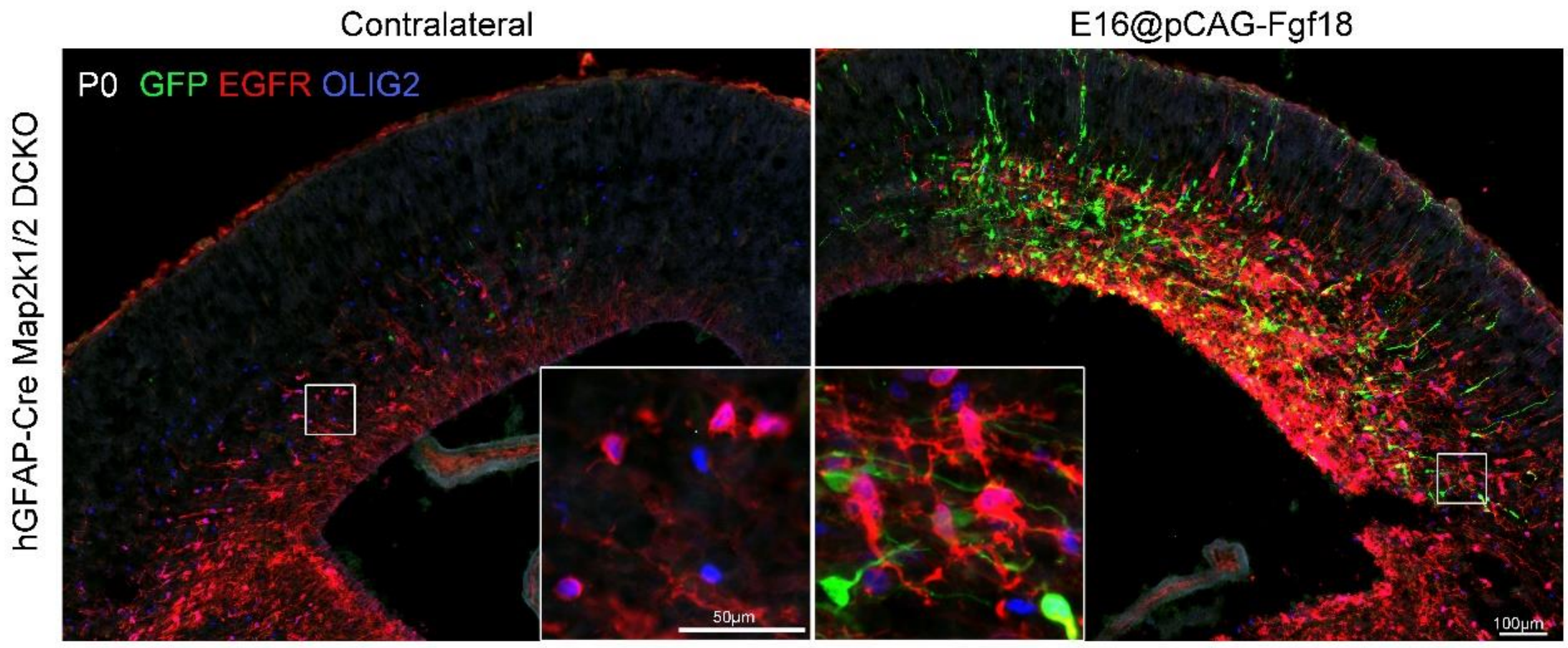

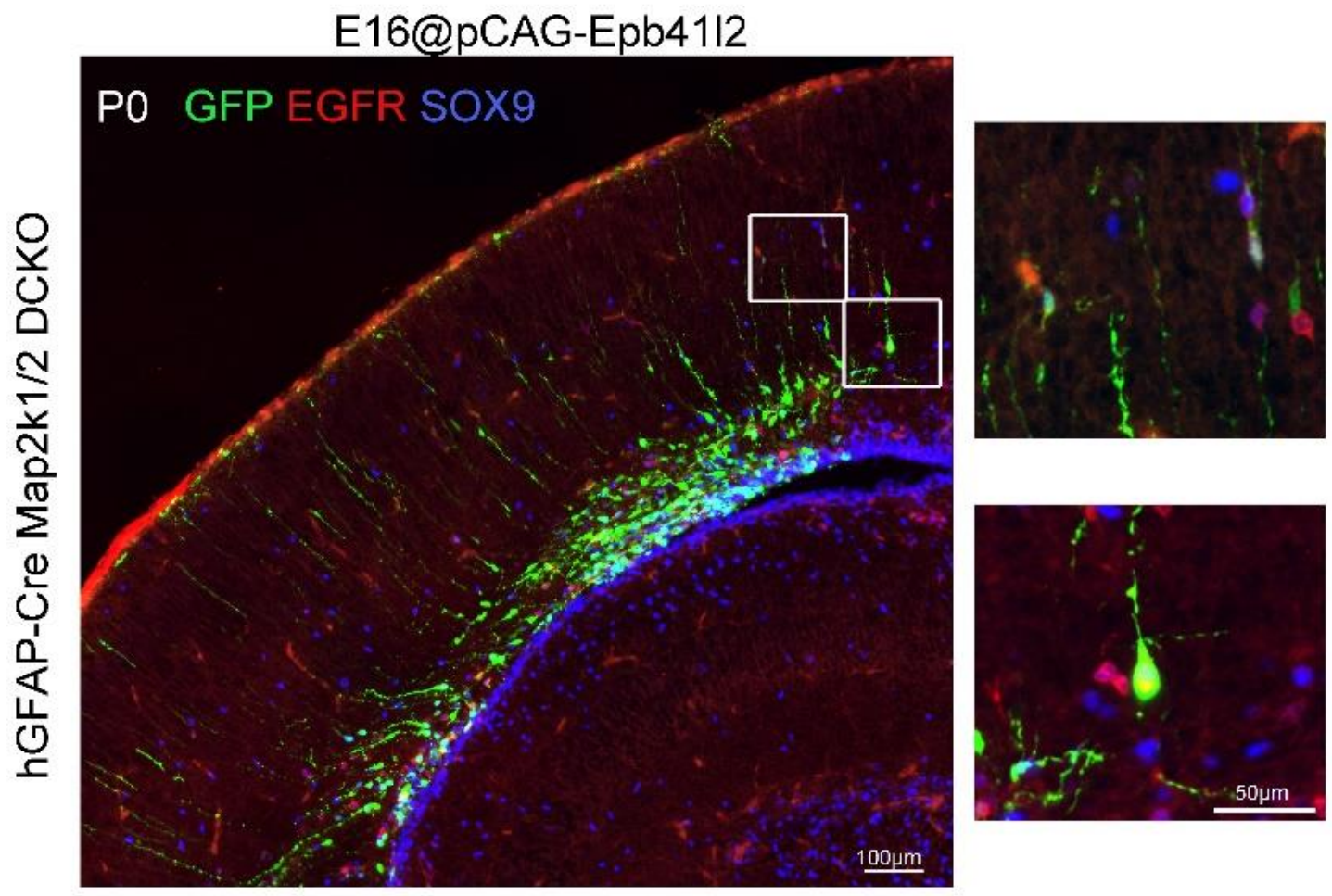

Fgf18 or Epb41l2 were electroporated into the Map2k1/2 DCKO cortex at E16, and analysed at P0.

Further, the specific ligand pairs (SEMA6) could be interrupted during this early time period to show that specific astrocyte-neuron crosstalk is important in mediating these behaviours, not just dendritic arborization (as alterations in signaling in the cortex was not necessarily linked mechanistically to behaviour).

Following your suggestions, we knockdown Plxna2/4 in the PFC region via in utero electroporation at embryonic day 13 and performed behavioral tests including the FST, TST, and SPT at P60 (Shown below, Fig. 5e). These mice exhibited depressive-like behaviors, similar to those observed in Egfr KO mice. In addition, we recorded long-term potentiation (LTP) in Plxna2/4-knockdown neurons at P60. Our data demonstrate that Plxna2/4 knockdown reduced dendritic complexity but did not affect dendritic spine density or the amplitude of LTP, as shown in the subsequent figures. These experiments significantly enhance the depth and validity of our study.

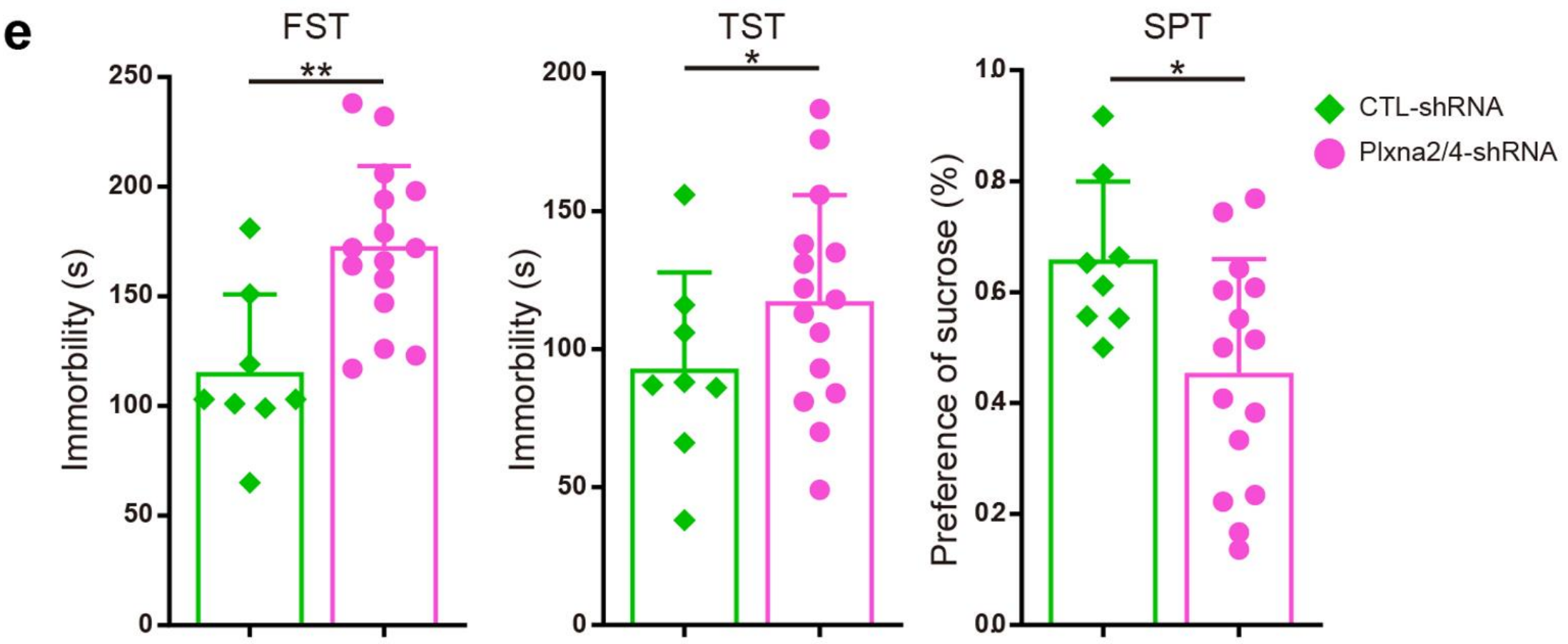

Plxna2/4-shRNA were electroporated in the PFC region at embryonic day 13 and performed behavioral tests including the FST, TST, and SPT at P60.
Plxna2/4-shRNA was electroporated into the PFC at E13. A series of behavioral tests, including the FST, TST, and SPT, were performed from P60.

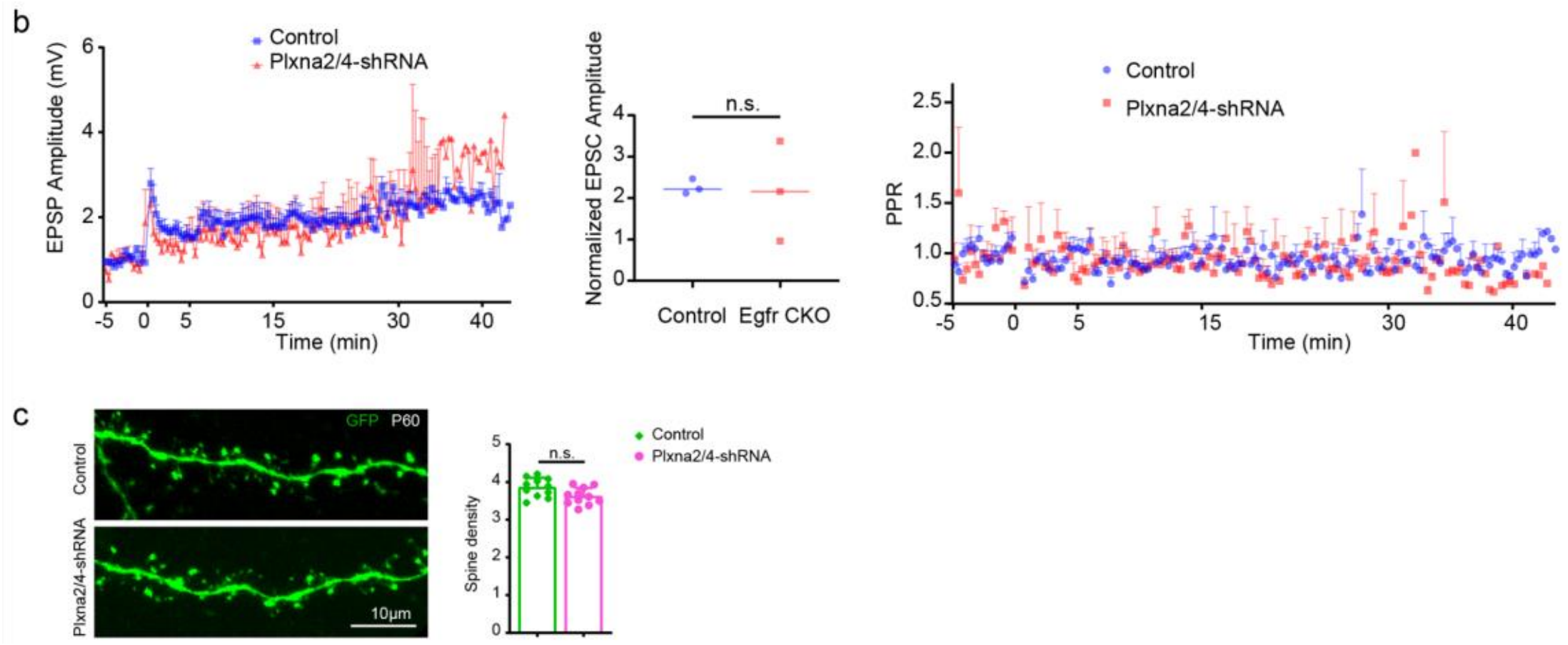

b-c. Knockdown of Plxna2/4 was performed in the mPFC via in utero electroporation at E13 and analyzed at 2 months. The data showed that neither the amplitude of long-term potentiation (LTP) nor dendritic spine density was significantly altered in the Plxna2/4-knockdown cortex compared with controls.

Additionally, there are some areas where clarifications could be implemented:
Results and Rationale Clarifications
1. Figure S1A – how did the authors confirm these were astrocytes?

We confirm these were astrocytes through SOX9 staining. We apologize for not displaying this staining (updated in Figure S1a).

2. The authors assert RGC give rise to astrocytes in a lateral to medial pattern, but there is staining in the medial regions with those injected at E18? Perhaps instead, there is more broad patterning from earlier time points. Same for SF3 EGFR KO.

We apologize for the inaccuracies in the description within our manuscript. As noted by Ge and colleagues, astrogenesis in the postnatal cortex occurs through local proliferation of astrocyte precursors rather than from radial glial cells (RGCs) [8]. Furthermore, Clavreul et al. demonstrated that postnatal cortical RGCs generate astrocytes in a stochastic manner [9]. In our study, we observed that RGCs produce astrocytes following a lateral-to-medial pattern during the first postnatal week.

Reference:

8 Ge, W. P., Miyawaki, A., Gage, F. H., Jan, Y. N. & Jan, L. Y. Local generation of glia is a major astrocyte source in postnatal cortex. Nature 484, 376-380 (2012). https://doi.org/10.1038/nature10959

9 Clavreul, S. et al. Cortical astrocytes develop in a plastic manner at both clonal and cellular levels. Nat Commun 10, 4884 (2019). https://doi.org/10.1038/s41467-019-12791-5

3. Fig 1 title should be updated, the figure doesn't show that embryonic EGFR KO leads to delayed crosstalk. The authors should include aspects of SF4e in Figure 1 and instead say that Egfr cKO leads to altered astrocyte organization. What time point is FS4e from? Are there just less connections because there are less astrocytes?

We fully agree with your suggestion and have implemented the changes accordingly. We have exchanged panel SF4e with Fig. 2f and updated the title of Figure 1. Additionally, please note that the images in Figure S4e are from postnatal day 2 (P2) samples, as marked in panel Fig. 2d.
The line thickness in CellChat networks reflects normalized communication probability, calculated as (ligand expression × receptor expression) / cell count [10]. This per-capita normalization ensures thickness represents signaling strength per cell rather than population size.

Reference:

10 Jin, S. et al. Inference and analysis of cell-cell communication using CellChat. Nat Commun 12, 1088 (2021). https://doi.org/10.1038/s41467-021-21246-9

4. How Fgfr 1 and 3 and Fgf18 relate to MAPK signaling and then both to Egfr could be better described in the text.

Thank you for your reminder. EGFR and FGFR synergistically regulate the ERK/MAPK pathway[11] and both are required for optimal cell proliferation and cell migration[11-14]. We observed a decrease in MAPK-positive cells in the subventricular zone (SVZ) and intermediate zone (IZ) of Egfr KO mice at P0, while an upregulation of MAPK protein levels was detected in the ventricular zone (VZ). This observation prompted us to investigate whether other signaling pathways—such as Fgf signaling—might contribute to MAPK activation in the VZ. scRNA-Seq data revealed expression of

Fgfr1 and Fgfr3 in radial glial cells (RGCs) of Egfr-CKO mice (Fig. S2C). In support of this, Hasegawa et al. reported that cortical neurons express Fgf18, a ligand capable of activating MAPK signaling through Fgfr1 and Fgfr3[15]. Therefore, we sought to determine whether MAPK activation by Fgf18 could ameliorate the phenotypic defects observed in Egfr-CKO mice.

Reference:

11 Sütterlin, P. et al. The molecular basis of the cooperation between EGF, FGF and eCB receptors in the regulation of neural stem cell function. Mol Cell Neurosci 52, 20-30 (2013). https://doi.org/10.1016/j.mcn.2012.10.006

12 Chen, T. et al. Fibroblast growth factor 18 promotes proliferation and migration of H460 cells via the ERK and p38 signaling pathways. Oncol Rep 37, 1235-1242 (2017). https://doi.org/10.3892/or.2016.5301

13 Li, Z., Liu, S. & Cai, Y. EGFR/MAPK signaling regulates the proliferation of Drosophila renal and nephric stem cells. J Genet Genomics 42, 9-20 (2015). https://doi.org/10.1016/j.jgg.2014.11.007

14 Ornitz, D. M. & Itoh, N. The Fibroblast Growth Factor signaling pathway. Wiley Interdiscip Rev Dev Biol 4, 215-266 (2015). https://doi.org/10.1002/wdev.176

15 Hasegawa, H. et al. Laminar patterning in the developing neocortex by temporally coordinated fibroblast growth factor signaling. J Neurosci 24, 8711-8719 (2004). https://doi.org/10.1523/jneurosci.3070-04.2004

5. While EPB41L2 certainly attenuates the effects of Egfr KO, it doesn’t fully rescue it, so some toning down of language here is warranted.

We agree with the reviewer's comment. We have toned down our language in the Results and Disscussion to accurately clarify that EPB41L2 attenuates but does not fully rescue the phenotypes resulting from Egfr KO.

6. Golgi analysis performed at P40 and biocytin performed at P50. Do the authors think there is potentially a difference there? Were both analyzed from Layer V? Were the dendrites analyzed for spines ones that were from distances from the soma where there were differences observed (50-100 um apx?) or further out (200 onward)?

We conducted Golgi staining analysis across multiple developmental stages (P7, P14, P40, P50, and 6 months) and observed a reduction in dendritic complexity in Egfr KO mice at the time points after P7. In accordance with your suggestion, we replaced the P40 time point with P50 in the analysis (Figure 4a).

This study focuses on the structural plasticity of dendritic spines located on the proximal basal dendrites (50–100 μm from the soma) of layer 5 pyramidal neurons in

the prelimbic cortex. These dendritic segments primarily receive excitatory synaptic inputs from local intracortical projections, particularly from neurons in layers 2/3 and 4 [16], and occupy a critical position in prefrontal microcircuit integration. Although this study does not directly address the downstream projection targets of mPFC or subcortical structures, the systematic analysis of spine density and morphology on L5 pyramidal neurons provides structural insight into local cortical information processing. These findings may contribute to understanding circuit-level dysfunctions in the mPFC associated with depressive-like states.

Reference：

16 Petreanu, L., Mao, T., Sternson, S. M. & Svoboda, K. The subcellular organization of neocortical excitatory connections. Nature 457, 1142-1145 (2009). https://doi.org/10.1038/nature07709

7. Please provide citations for the neuronal alterations and psychiatric disorders. Where in the PFC were these analyzed? Do the authors mean the pre- or infralimbic or cingulate cortices (medial PFC) specifically or are these from anywhere from those regions to the M1 and S1 cortices? This is especially important as the prelimbic and infralimbic cortices can have opposing actions on behaviour.

Neuronal alterations play a critical role in the pathogenesis of psychiatric disorders, as evidenced by structural and functional changes in the cytoskeleton and cellular morphology. In autism spectrum disorder (ASD), abnormalities in actin filament polymerization and associated proteins disrupt neuronal connectivity, contributing to behavioral symptoms and clinical manifestations[17]. Similarly, postmortem studies in major depression reveal significant cellular-level alterations, including dendritic atrophy or hypertrophy, changes in neuronal density, and selective neuronal loss in key brain regions[18-20]. Cao et.al found that early life inflammation promotes excessive microglial engulfment of glutamatergic neuronal spines in ACC and causes depressive symptoms in adolescence[21]. These findings highlight how cytoskeletal dysregulation and neuronal remodeling underlie the neurobiological basis of psychiatric conditions, offering potential targets for therapeutic intervention.

We apologize for the imprecision in our description of the brain regions. The Golgi stain and electrophysiological experiments were conducted in the prelimbic (PrL) and infralimbic (IL) cortices, two subregions of the medial prefrontal cortex (mPFC). These regions are anatomically adjacent but functionally dissociable: The PrL has been associated with the expression of goal-directed and high-arousal behaviors, whereas the IL is more involved in inhibitory control, fear extinction, and affective regulation[22-24]. Our study focused on whether neurons in both areas exhibit similar cytoskeletal and electrophysiological alterations following delayed development of astrocytes. We did not aim to compare their potentially opposing influences on behavior, which we agree is an important question for future work. We have clarified these points in the revised Methods.

Reference:

17 Falougy, H. E., Filova, B., Ostatnikova, D., Bacova, Z. & Bakos, J. Neuronal morphology alterations in autism and possible role of oxytocin. Endocr Regul 53, 46-54 (2019). https://doi.org/10.2478/enr-2019-0006

18 Stockmeier, C. A. et al. Cellular changes in the postmortem hippocampus in major depression. Biol Psychiatry 56, 640-650 (2004). https://doi.org/10.1016/j.biopsych.2004.08.022

19 Krishnan, V. & Nestler, E. J. The molecular neurobiology of depression. Nature 455, 894-902 (2008). https://doi.org/10.1038/nature07455

20 Boldrini, M. et al. Hippocampal granule neuron number and dentate gyrus volume in antidepressant-treated and untreated major depression. Neuropsychopharmacology 38, 1068-1077 (2013). https://doi.org/10.1038/npp.2013.5

21 Cao, P. et al. Early-life inflammation promotes depressive symptoms in adolescence via microglial engulfment of dendritic spines. Neuron 109, 2573-2589.e2579 (2021). https://doi.org/10.1016/j.neuron.2021.06.012

22 Sierra-Mercado, D., Padilla-Coreano, N. & Quirk, G. J. Dissociable roles of prelimbic and infralimbic cortices, ventral hippocampus, and basolateral amygdala in the expression and extinction of conditioned fear. Neuropsychopharmacology 36, 529-538 (2011). https://doi.org/10.1038/npp.2010.184

23 Vertes, R. P. Differential projections of the infralimbic and prelimbic cortex in the rat. Synapse 51, 32-58 (2004). https://doi.org/10.1002/syn.10279

24 Warden, M. R. et al. A prefrontal cortex-brainstem neuronal projection that controls response to behavioural challenge. Nature 492, 428-432 (2012). https://doi.org/10.1038/nature11617

8. Since the authors also utilized females, did they analyze darting in the fear conditioning responses?

In our study, both male and female mice were used for conditioning behavioral responses. Video analysis revealed that neither sex exhibited darting behavior during CS-US trials or CS tests, as reported previously[25]. Freezing was the predominant behavioral response observed. The C57 genetic background of these mice may contribute to their reduced probability of displaying darting behavior [26].

Reference:

25 Gruene, T. M., Flick, K., Stefano, A., Shea, S. D. & Shansky, R. M. Sexually divergent expression of active and passive conditioned fear responses in rats. Elife 4 (2015). https://doi.org/10.7554/eLife.11352

26 Kafkafi, N. et al. Darting behavior: a quantitative movement pattern designed for discrimination and replicability in mouse locomotor behavior. Behav Brain Res 142, 193-205 (2003). https://doi.org/10.1016/s0166-4328(03)00003-2

9. Yes, the FST, TST and SPT are often utilized to assess “depressive-like behaviours” (or rather anti-depressant therapeutic screening). However, to consider being more

specific and discuss that these leads to a reduction of active coping or a switch to passive coping in the FST and TST and an increase in anhedonia in SPT.

We completely agree with your point regarding the need for more precise language when describing the outcomes of the behavioral tests. We apologize for the oversimplification and the use of anthropomorphic terms in our original description. To clarify their behavioral implications more precisely (This has now been updated in the manuscript): FST/TST measure a shift from active coping (e.g., climbing/struggling) to passive coping (immobility), reflecting behavioral despair rather than direct depression modeling. The Sucrose Preference Test (SPT) was performed to assess anhedonia, a core symptom of depression that is characterized by a reduced preference for a sucrose solution over plain water.

10. Why do the authors think there was an increase in open arm entries in the EPM? This does not necessarily indicate a locomotor effect. And the mice do not spend more time there, so what is happening?

Thank you for this insightful and critical comment. You raise an excellent point regarding the interpretation of the elevated plus maze (EPM) data. We conduct a more nuanced, fine-grained analysis of the animals' behavior to accurately discern their motivational state. The key distinction, as you rightly point out, is between increased exploration driven by reduced anxiety and non-specific hyperlocomotion.

To address this, we performed a new analysis of risk-assessment behaviors, which are highly sensitive measures of conflict and anxiety in the EPM. We specifically quantified protected head-dip (executed from within the closed arms or center square), unprotected head-dips (executed while on the open arms) and Stretch-attend postures (SAP)[27].

We observed a significant increase in unprotected head-dips in the treated group compared to controls (P = 0.0326). This finding is crucial, as an unprotected head-dip represents active exploration conducted from within the anxiogenic open arm. This indicates a greater willingness to engage in investigative behavior while in a potentially threatening environment. In contrast, the number of protected head-dips and stretch-attend postures (SAP) remained unchanged. This suggests that the initial decision-making process—the assessment of risk from the safety of the closed arms—was not altered.

Therefore, we interpret th*e dissociation betw*een increased open arm entry and unchanged open arm time as follows: Egfr deficient did not induce general hyperactivity nor did it alter the initial appraisal of risk. Instead, it specifically increased exploratory motivation and confidence once the animal was in the open arm. The mice entered the open arms more frequently and, upon entry, engaged in significantly

more active exploration (unprotected head-dips). This finding, combined with the unchanged total open arm time, indicates that the animals made more frequent, but shorter, visits to the open arms. The significant increase in unprotected head-dips demonstrates that these shorter visits were dedicated to active exploration rather than passive occupancy. We interpret this as a confident pattern of risk-assessment: they are entering the aversive space not to remain there, but to purposefully gather information before returning to safety.

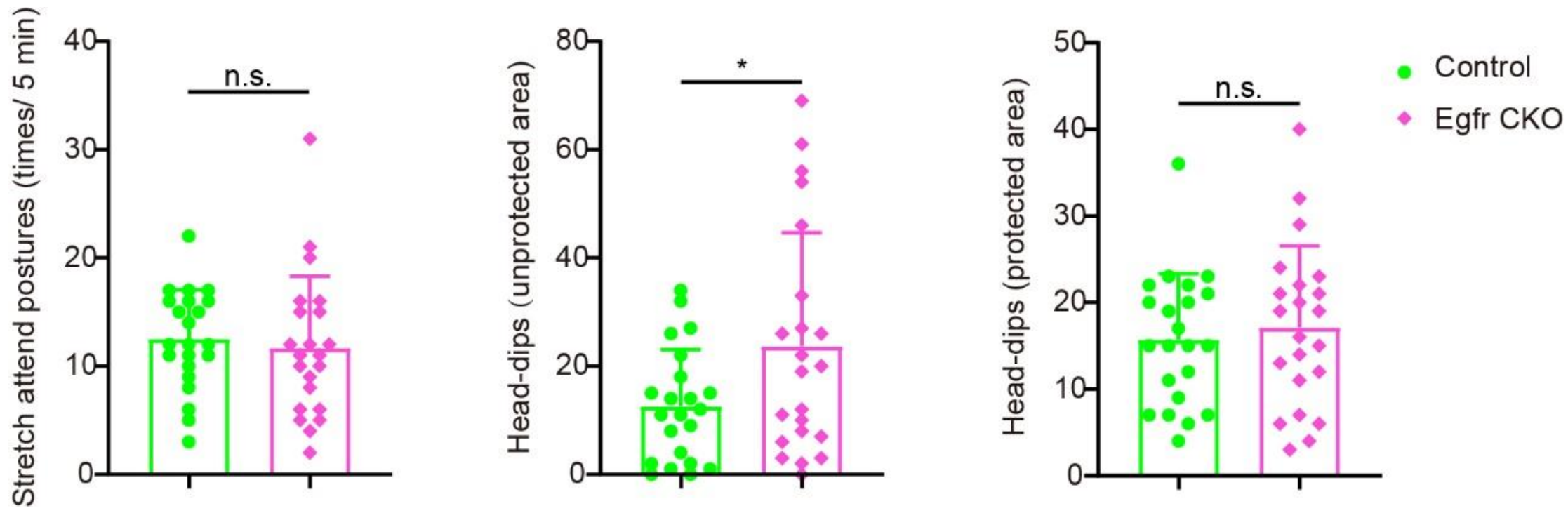

We believe this pattern of results—selective increase in unprotected exploratory behavior—strongly supports a specific anxiolytic-like effect rather than a locomotor confound. We are grateful for your comment, which has led to a more sophisticated and robust interpretation of our data. We will revise the manuscript accordingly to include these new findings and refine our discussion.

Reference：

27 Walf, A. A. & Frye, C. A. The use of the elevated plus maze as an assay of anxiety-related behavior in rodents. Nat Protoc 2, 322-328 (2007). https://doi.org/10.1038/nprot.2007.44

11. Did the authors consider assessing any additional behaviours, risk perception, repetitive etc, in their behavioural tasks?

We thank you for this insightful comment. We agree that assessing additional behavioural dimensions such as risk perception and repetitive behaviours would provide a more comprehensive characterization of the behavioural phenotype. As detailed in our response to comment #10 above, we have now included a discussion on the importance of assessing risk perception.

To determine if Egfr deficiency leads to repetitive behaviors, we quantified grooming, jumping, and circling in the open field test. The EGFR-deficient mice did not differ from control mice in any of these measures. This finding further demonstrates the circuit specificity of their neurodevelopmental deficits. It indicates that the delayed development of astrocytes primarily affects higher brain regions

mediating emotional integration (such as the prefrontal cortex and its associated limbic circuits)[28,29], while sparing the dorsal striatal pathways primarily responsible for habit formation and motor execution. This dissociation underscores that the regulation of neural circuits by developing astrocytes exhibits high regional and functional specificity, and their defects can lead to discrete, rather than global, behavioral phenotypes.

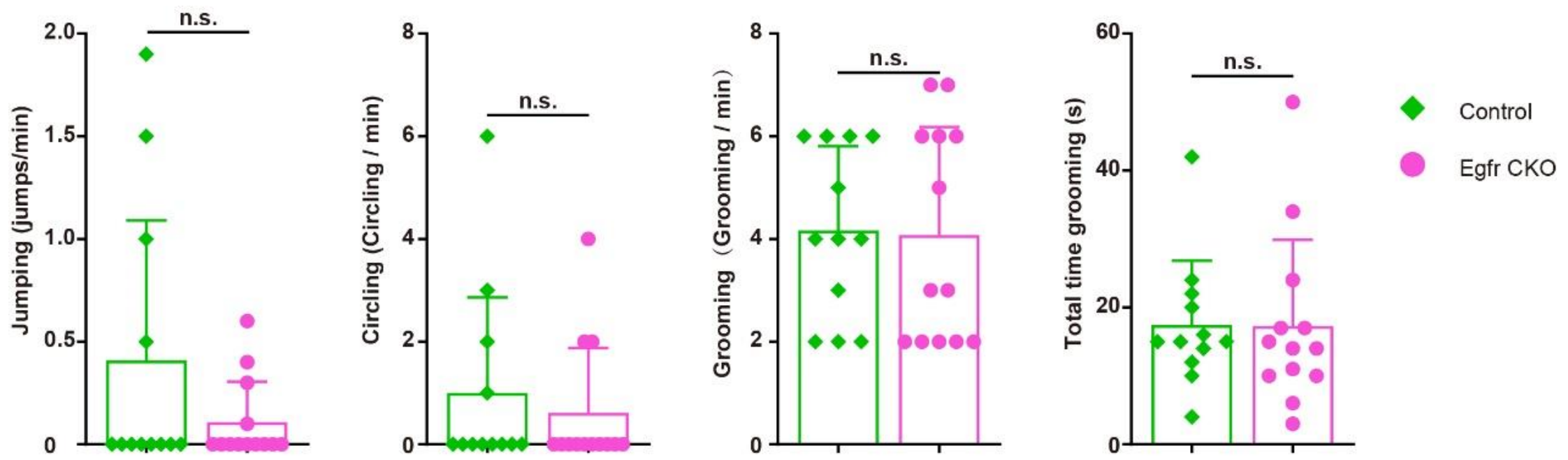

Reference：

28 Ahmari, S. E. & Dougherty, D. D. DISSECTING OCD CIRCUITS: FROM ANIMAL MODELS TO TARGETED TREATMENTS. Depress Anxiety 32, 550-562 (2015). https://doi.org/10.1002/da.22367

29 Aliane, V., Pérez, S., Bohren, Y., Deniau, J. M. & Kemel, M. L. Key role of striatal cholinergic interneurons in processes leading to arrest of motor stereotypies. Brain 134, 110-118 (2011). https://doi.org/10.1093/brain/awq285

12. Why would Sema6a-Plxna2/4 expression be elevated in the Egfr cKO, if this pathway is proposed to be part of the disrupted signaling between astrocytes and neurons?

As addressed in our response to the main concern, the expression of the Sema6a–Plxna2/4 receptor pair is not significantly altered in Egfr cKO and Map2k1/2 cKO compared with control (shown below). However, Sema6a-expressing astrocytes in the Egfr cKO cortex fail to migrate outward, thereby preventing direct contact with Plxna2/4-expressing neurons—a necessary condition for Sema6a–Plxna2/4 interaction and signaling.

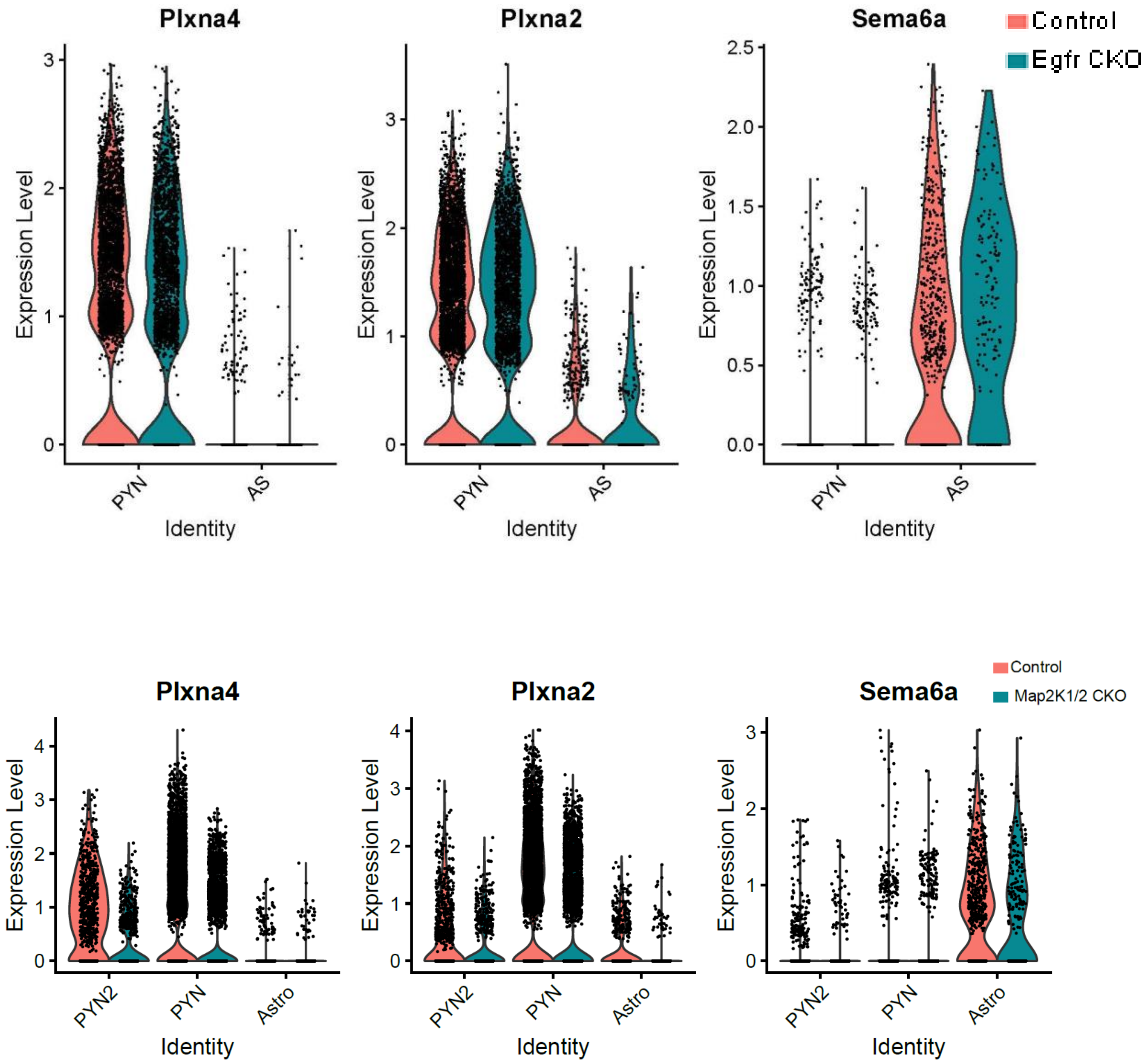

Violin plot of Sema6a, Plxna2 and Plxna4 expression in projection neurons (PYN) and astrocytes (AS). Data from the sc-RNA seq of P2 cortex.

13. Saying that “EPB41L2, PLXNA2, and PLXNA4 has a direct and significant causal effect on MDD” (Line 299), is an overstatement. Consider using associated with MDD, as is in the figure legend.

Thank you for catching this. You are absolutely right; ‘causal effect’ is an overstatement here. I've revised the text to say ‘is significantly associated with MDD’ to be more precise. The change has been made.

Methods Clarifications

14. Please confirm how many animals per litters were used for each endpoint. In general, no more than 2 mice per sex should be included unless combining for a single “n”. Please confirm if litters were considered. Were dams nulliparous prior to

experiment or no? Just to also confirm, were males and females equally utilized throughout all experiments?

We thank the reviewer for this critical question regarding the litter effect, which is a fundamental principle in developmental studies. We acknowledge that our experimental design did not strictly adhere to the ideal practice of using only one pup per sex per litter for each endpoint. We apologize for this oversight. All experimental groups were composed of offspring from a minimum of 6 independent litters. This ensured that our findings are not driven by a single litter's characteristics.

We confirm that all dams used in this study were nulliparous prior to the mating that generated the experimental offspring.

We confirm that both male and female offspring were used equally and throughout all experiments.

15. At what age were mice considered adults? 7-8 week old is still late adolescence to very early adulthood. Were they P60 or 7-8 weeks old (P49-P56) for behaviour?

We sincerely apologize for the previous miscalculation in our description. Since mice reach maturity at approximately postnatal day 60 (P60), behavioral testing began at P60. The entire experimental procedure required approximately 2–3 weeks to complete. Therefore, the age of the mice during testing ranged from 8 to 11 weeks old.

16. Please indicate the anatomical coordinates and sub-regions included in PFC for RNAseq studies (and the rest of the studies). Unclear the differentiation of regions in Figure S1b, there is potentially some overlap in those regions divided out. None of them would technically include the IL or PL, correct? But later in the manuscript, these are proposed as important regions for mediating these effects.

We thank the reviewer for this critical comment and the opportunity to clarify this important point. We apologize for the lack of clarity in our description of the prefrontal cortex (PFC) sub-regions sampled for RNA-seq. In our study, the tissue samples for RNA-seq were micropunched from the medial prefrontal cortex (including PrL, IL, ACC, along with a minimal portion of the primary motor cortex) of mice based on the Paxinos and Franklin mouse brain atlas (4th edition). We will amend the Methods section to explicitly state the anatomical coordinates (Bregma 1.98-1.70) and the specific sub-regions (PL, IL, ACC) that were included in our 'PFC' samples.

The regions are delineated according to the atlas shown below.

We total agree with you that none of them would technically include the IL or PrL. As outlined in the response to Question 2, this study aims to determine whether postnatal RGCs give rise to astrocytes and, if so, whether this process is random. However, our initial experimental design did not include the prefrontal cortex (PFC); it emerged as a focus as the research progressed.

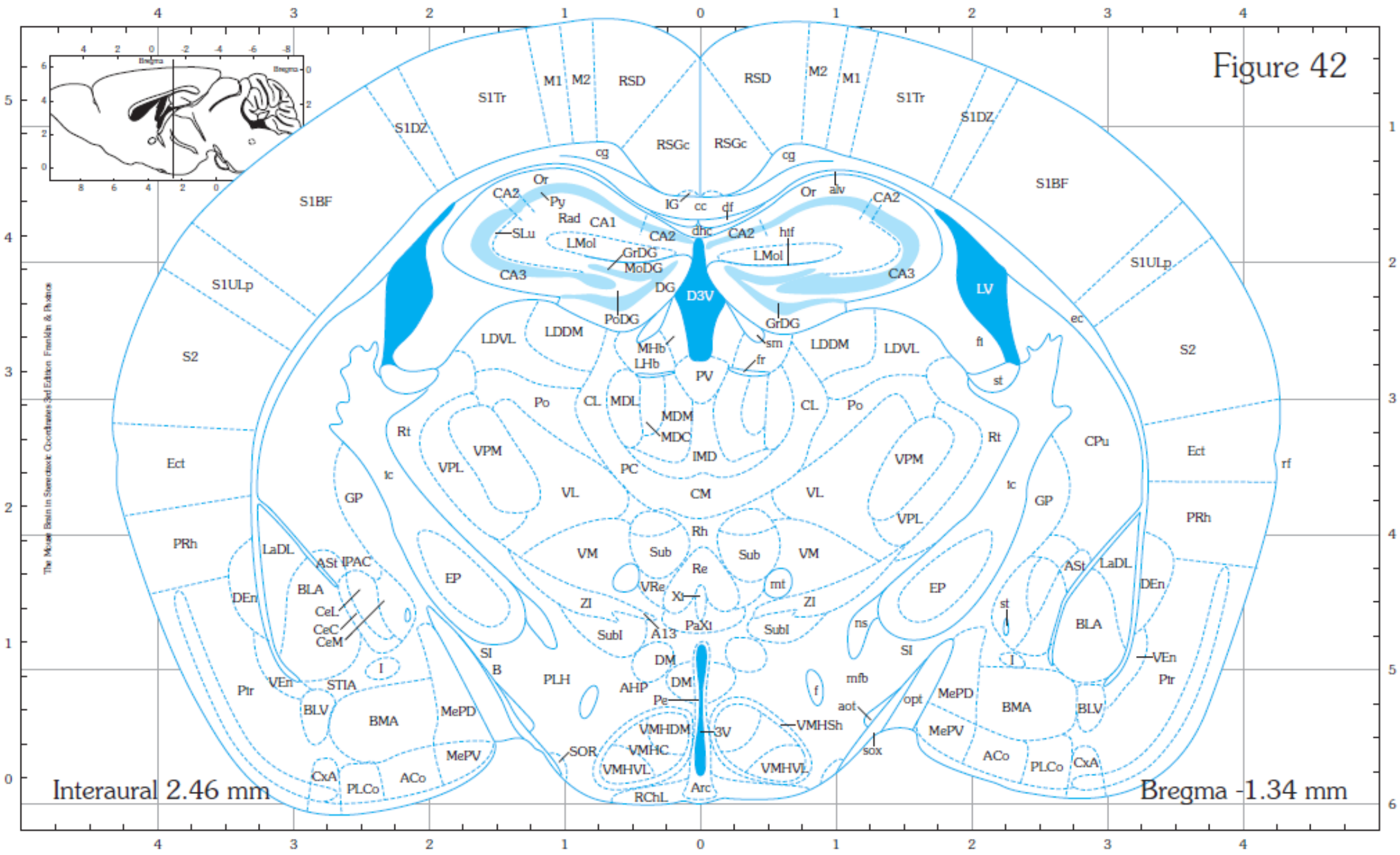

17. Missing many details about the many of the specific overexpression (when during development or sequences) in the text.

We thank the reviewer for this important comment. We agree that these details are crucial and have now comprehensively expanded the 'In Utero Electroporation' subsection in the Methods section and Supplementary Table 6.

18. The authors should consider a nested t-test for their ephys data to use animal as the "n."

We are grateful to the reviewer for raising this important point regarding statistical analysis. We have now re-analyzed our electrophysiology data using a nested approach (accounting for the non-independence of cells within the same animal) as recommended. Reassuringly, the results of this corrected analysis remain statistically significant and fully support our original conclusions (shown below). We have updated the Statistics subsection to the Supplementary Table 5, the p value in relevant figures, and the Results section to reflect this more rigorous analysis.

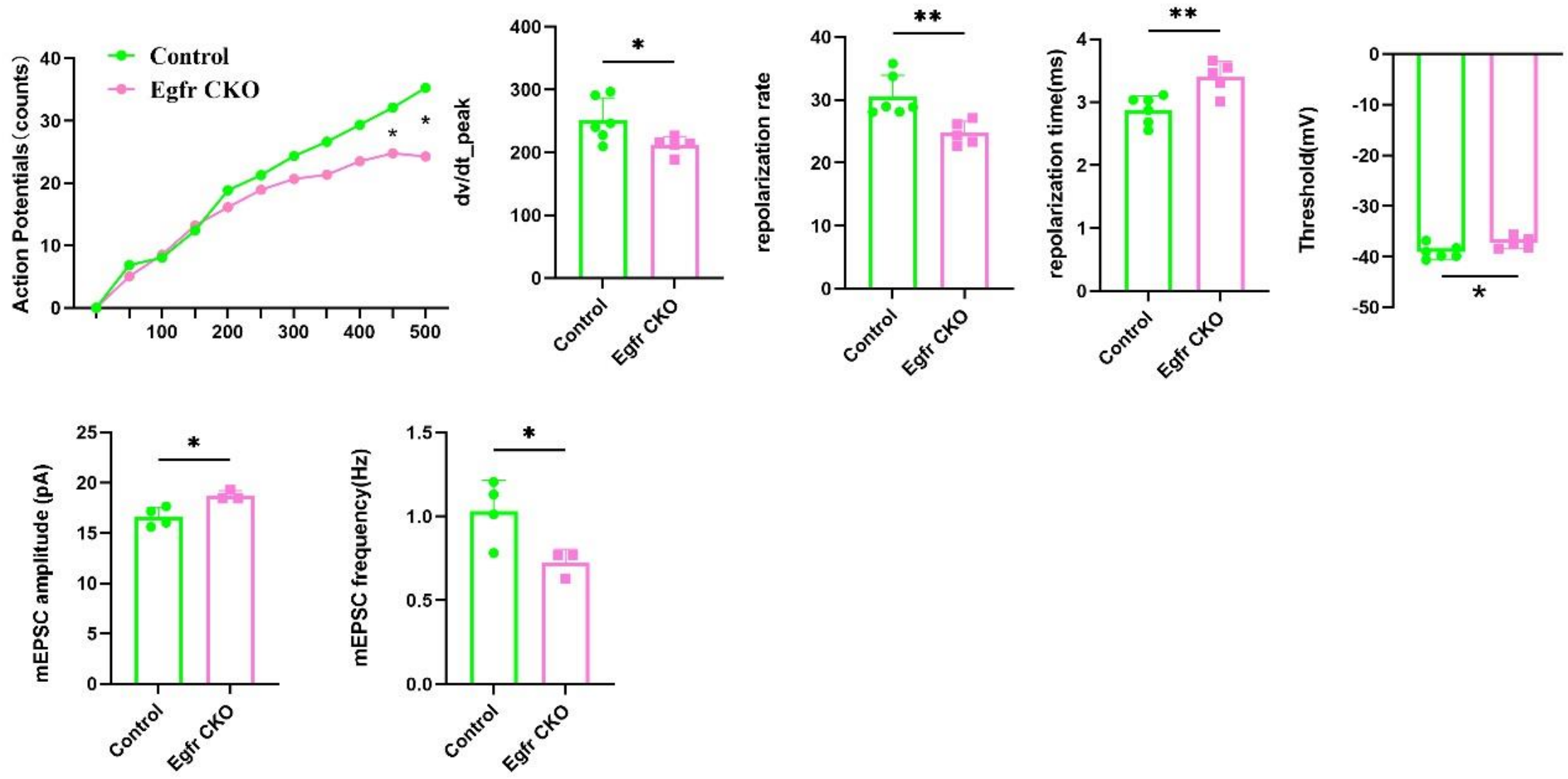

19. What was the order of the behavioural tasks? Were any performed on the same day?

The order of behavioral tests should follow the principle of "from low to high stress intensity", i.e., starting with less stressful tests before proceeding to more stressful ones. In this study, the experimental sequence is as follows: Open Field Test-Novel Object Recognition- Y-Maze- Elevated Plus Maze-Rotarod Test-Grip Strength Test-Fear Conditioning-Sucrose Preference Test -Tail Suspension Test-Forced Swim Test. A 7-day interval was maintained between fear conditioning and sucrose preference testing. Only one behavioral test was performed per day.

20. Statistics should be reported (i.e., t- and p-values should be reported in a supplemental table).

We thank you for this suggestion. We have now added a new Supplementary Table (Supplementary Table 5) that reports the complete statistical details for all quantitative analyses, including the specific tests used, test statistics (e.g., *t*, F), and exact *p*-values. This table is now referenced in the relevant figure legends and the Methods section.

Reviewer #3 (Remarks to the Author)

I co-reviewed this manuscript with one of the reviewers who provided the listed reports. This is part of the Nature Communications initiative to facilitate training in

peer review and to provide appropriate recognition for Early Career Researchers who co-review manuscripts.

We are grateful for your time and constructive suggestions, which have greatly improved our article.

Reviewer #4 (Remarks to the Author)

We are extremely grateful for your constructive suggestions, which have helped substantiate the conclusions. We have now carefully addressed all of your suggestions, either by providing clarification or by performing new experiments. Point-by-point responses to your comments are given below, with your comments given verbatim in black text, and our responses in purple:

This manuscript presents a comprehensive investigation into the role of delayed astrocyte-neuron crosstalk on neurodevelopment and its behavioral consequences using an Egfr knockout mouse model. The study effectively elucidates the critical importance of astrocyte-neuron interactions during the developmental critical period, highlighting the molecular mechanisms underlying impaired crosstalk and its impact on neuronal morphology, excitability, and depressive-like behaviors. The findings offer valuable insights into the potential link between astrocyte dysfunction and major depressive disorder (MDD), suggesting novel therapeutic targets for psychiatric disorders.
The manuscript is well-structured, with clear articulation of hypotheses, detailed experimental design, and thorough data analysis. The integration of single-cell RNA sequencing, electrophysiology, and behavioral assays provides a robust and multidimensional approach to understanding the complex interplay between astrocytes and neurons. However, some points require clarification and elaboration.

We thank you for your insightful comments and positive evaluations.

Major Comments:
Additional investigation into the compensatory mechanisms would strengthen the paper. The authors show that neurons exhibit reduced excitability and dendritic complexity in adulthood, but it remains unclear whether these changes are permanent or potentially reversible. This has important implications for the fundamental roles of neuron-astrocyte communication that should be addressed.

We sincerely thank the reviewer for this insightful and constructive comment. We agree that investigating the potential reversibility of the observed neuronal changes are crucial next steps to fully understand the roles of neuron-astrocyte communication. Consistent with the phenotype observed at P60, mPFC neurons in Egfr knockout mice continued to display reduced dendritic complexity at 6 months.

In addition, these mice similarly showed behavioral deficits, with a reduced sucrose preference and increased immobility in the FST and TST.

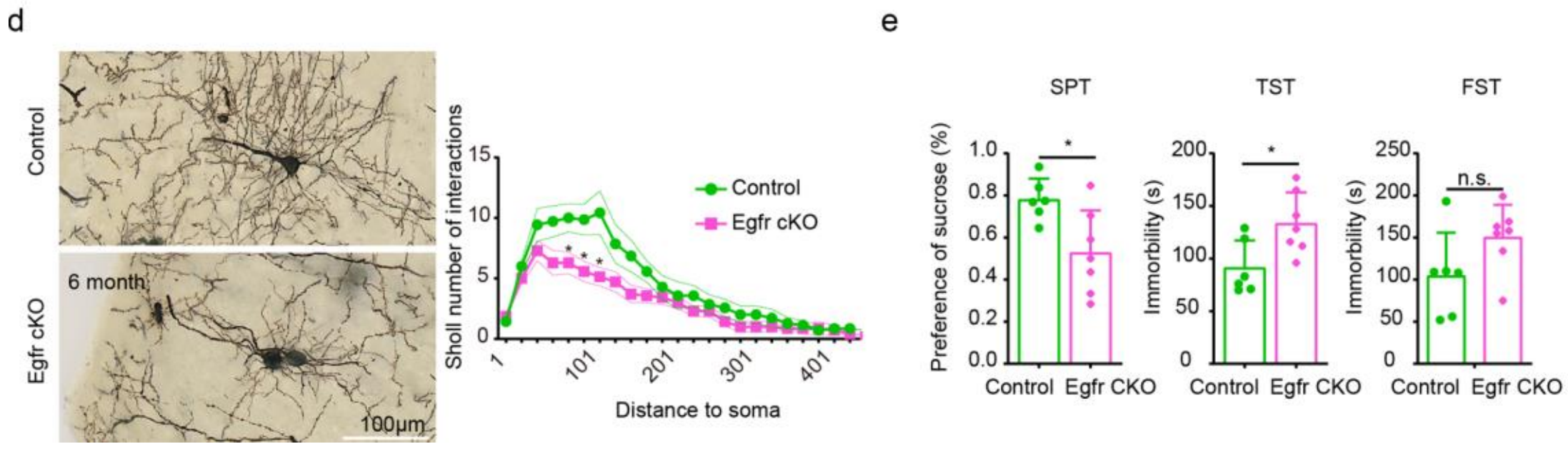

Analysis of dendritic morphology and depressive-like behaviors in 6-month-old Egfr KO and control mice. (d) Representative images and quantification of neuronal dendrites from the mPFC, revealed by Golgi staining. (e) Behavioral assessment including the sucrose preference test (SPT), forced swim test (FST), and tail suspension test (TST).

The manuscript presents compelling evidence linking the impaired EGFR-pERK-EPB41L2 axis to delayed astrocyte migration. However, the downstream effects on neuronal circuits are not fully elucidated. Additional experiments that validate the functional consequences of this impaired signaling on synaptic connectivity and network dynamics would strengthen the findings.

We thank you for your insightful suggestions. We initially found that Egfr KO leads to reduced dendritic complexity in neurons, while synaptic density on dendrites remained unchanged. Whole-cell patch-clamp recordings revealed a decrease in the frequency of miniature excitatory postsynaptic currents (mEPSCs). Together, these findings indicate an overall reduction in the total number of synaptic connections. In addition, we observed a decrease in the intrinsic excitability of Egfr KO neurons, characterized by reduced firing frequency, elevated action potential threshold, and slower repolarization rate.

However, an increase in mEPSC amplitude was detected, suggesting that despite the loss of synapses, the remaining ones may be functionally strengthened. This interpretation was further supported by molecular evidence: although western blot analysis showed only a mild, non-significant increase in total PSD95 protein, immunohistochemistry revealed brighter and larger PSD95 puncta, indicating a postsynaptic consolidation of this key scaffolding protein at individual synapses.

Subsequently, using optogenetic activation of callosal inputs, we identified a clear compensatory enhancement mechanism: increased oEPSC amplitude accompanied by decreased paired-pulse ratio (PPR), indicating elevated presynaptic neurotransmitter release probability as well as enhanced postsynaptic receptor function. Moreover,

under low-intensity light stimulation, a higher proportion of neurons were activated in the Egfr KO group, confirming their heightened sensitivity to inputs—i.e., under identical stimulation conditions, KO neurons were more likely to be activated. Together, these presynaptic and postsynaptic changes demonstrate a robust synaptic potentiation phenomenon. These data indicate that although Egfr KO weakens the overall structural foundation and intrinsic excitability of neurons, homeostatic plasticity mechanisms specifically strengthen the connectivity of the remaining synapses.

Nevertheless, this localized enhancement of synaptic function (coupled with simplified neuronal morphology and reduced intrinsic excitability) disrupted the balance of network activity. Ultimately, these alterations led to the emergence of depression-like behaviors in mice.

In summary, the circuit-level logic proceeds as follows: disruption of the EGFR–pERK–EPB41L2 axis first impairs structural connectivity (dendritic complexity) and intrinsic neuronal excitability. This, in turn, triggers a functional compensation through strengthening of remaining synapses, which disrupts the balance of network activity and finally results in depression-like behavior.

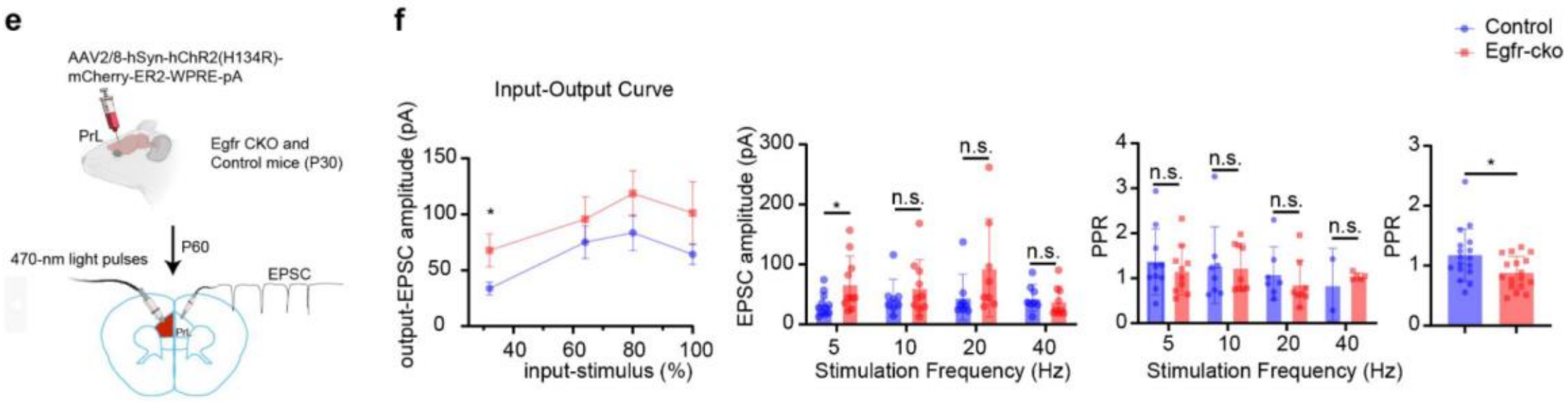

(e) Schematic diagram of the optogenetic experimental design in Egfr conditional knockout (CKO) mice and their control littermates. (f) Summary of the short-term synaptic dynamics recorded from the control and Egfr CKO groups. The input-output relationship is depicted in the line chart (left), while the bar plot (center) shows the response amplitude and paired-pulse ratio (PPR) at 32% intensity across various stimulation frequencies. An additional bar plot (right) details the PPR specifically at 5 Hz and 80% intensity.

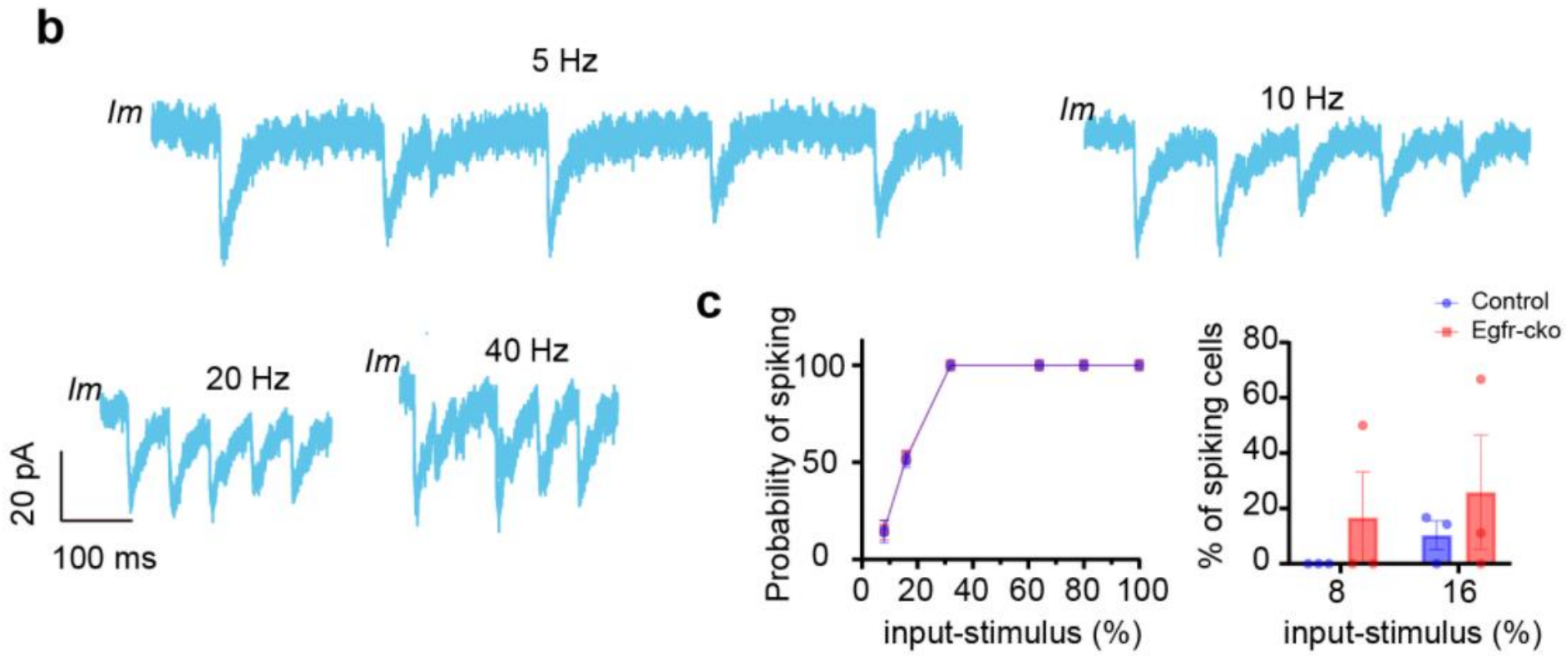

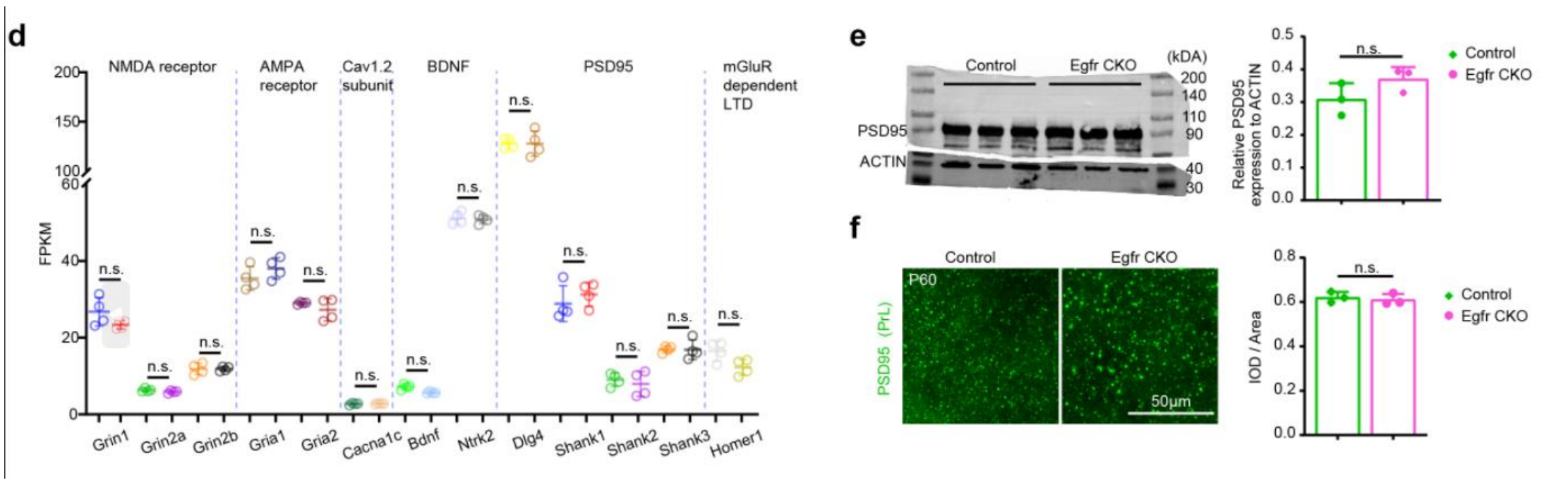

(b). Representative traces of synaptic dynamics in response to long-range input to the contralateral PFC across four stimulation frequencies (5, 10, 20, and 40 Hz; n = 3 mice per group, detailed cell numbers provided in Supplementary Table 5). (c). Left: Spike probability across a range of light intensities. Right: Percentage of activated cells in response to 8 Hz and 16 Hz stimulation. (d). RNA-seq analysis reveals no significant changes in the expression of synaptic plasticity-related genes in the PFC of Egfr CKO mice. (e–f). Western blot (e) and immunostaining (f) show unaltered PSD95 protein levels in the PFC of Egfr CKO mice (3 mice per group).

The role of the Sema6a-Plxna2/4 receptor pair is convincingly shown to influence dendritic complexity. Yet, its impact on synaptic plasticity and long-term potentiation (LTP) should be explored to provide a more comprehensive understanding of the behavioral phenotypes observed.

We thank the reviewer for this important suggestion. To evaluate the role of Sema6a-Plxna2/4 in synaptic plasticity, we performed in utero electroporation of shRNA targeting Plxna2/4 into the medial prefrontal cortex (mPFC) at E13.5 and measured long-term potentiation (LTP) using whole-cell recordings from cortical layer 5 pyramidal neurons. LTP serves as a key cellular model for how synaptic plasticity supports learning, memory, and other cognitive functions[30,31]. We found that the magnitude of LTP was comparable between control and Plxna2/4 konckdown groups (new data now included in Fig. S12b). Consistent with our behavioral findings from the Y maze, NOR, and Fear Conditioning tests, we observed no major cognitive impairments, which aligns with the intact LTP reported here.

Our results point to a model where Sema6a-Plxna2/4 signaling likely influences behavior through structural remodeling of dendritic arbors[332,33], a prerequisite for normal network computation[34], as opposed to directly altering synaptic potentiation. This distinction helps reconcile the intact LTP with the observed behavioral deficits, highlighting the critical role of structural plasticity in circuit function.

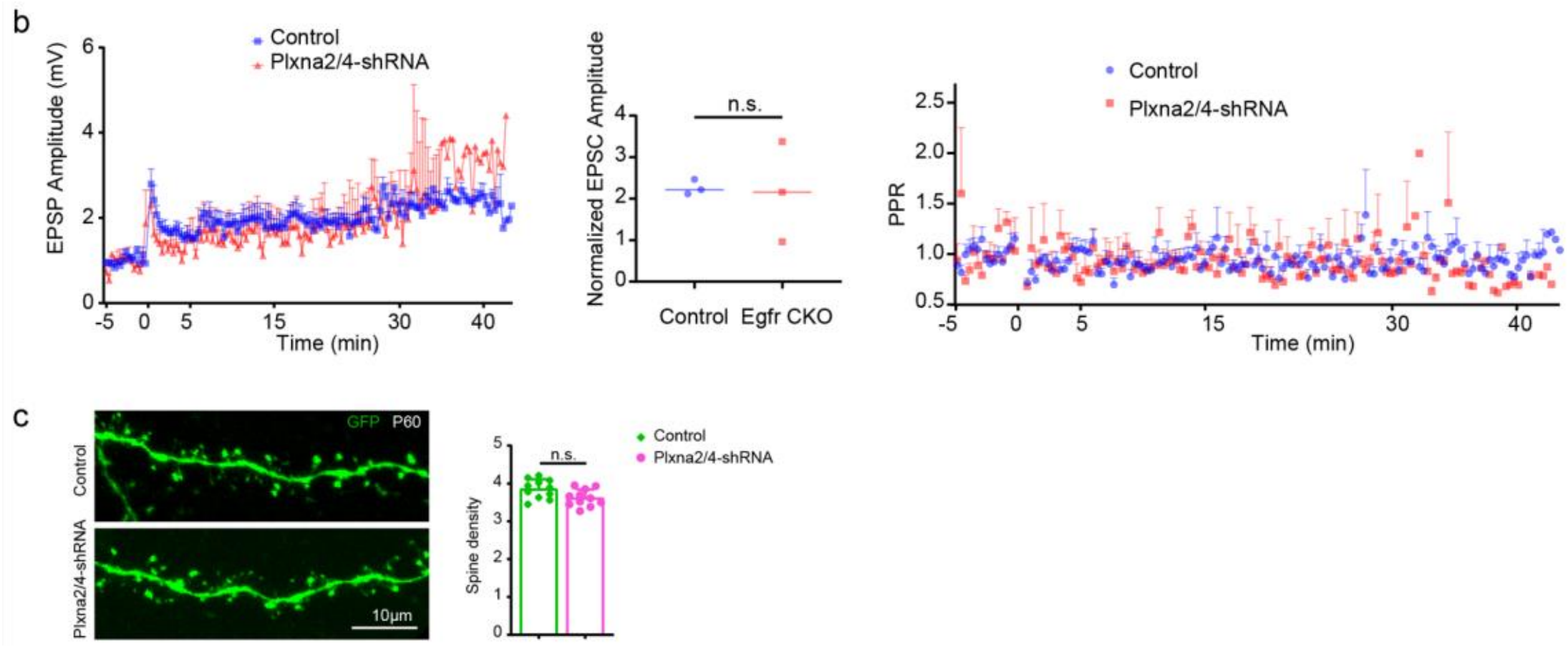

b-c. Knockdown of Plxna2/4 was performed in the mPFC via in utero electroporation at E13 and analyzed at 2 months. The data showed that neither the amplitude of long-term potentiation (LTP) nor dendritic spine density was significantly altered in the Plxna2/4-knockdown cortex compared with controls.

Reference：

30 Pałucha-Poniewiera, A., Bobula, B. & Rafało-Ulińska, A. The Antidepressant-like Activity and Cognitive Enhancing Effects of the Combined Administration of (R)-Ketamine and LY341495 in the CUMS Model of Depression in Mice Are Related to the Modulation of Excitatory Synaptic Transmission and LTP in the PFC. Pharmaceuticals (Basel) 16 (2023). https://doi.org/10.3390/ph16020288

31 Laroche, S., Davis, S. & Jay, T. M. Plasticity at hippocampal to prefrontal cortex synapses: dual roles in working memory and consolidation. Hippocampus 10, 438-446 (2000). https://doi.org/10.1002/1098-1063(2000)10:4<438::Aid-hipo10>3.0.Co;2-3

32 Holtmaat, A. & Svoboda, K. Experience-dependent structural synaptic plasticity in the mammalian brain. Nat Rev Neurosci 10, 647-658 (2009). https://doi.org/10.1038/nrn2699

33 Tran, T. S. et al. Secreted semaphorins control spine distribution and morphogenesis in the postnatal CNS. Nature 462, 1065-1069 (2009). https://doi.org/10.1038/nature08628

34 London, M. & Häusser, M. Dendritic computation. Annu Rev Neurosci 28, 503-532 (2005). https://doi.org/10.1146/annurev.neuro.28.061604.135703

While the depressive-like behaviors in Egfr knockout mice are well-documented, it would be beneficial to include additional behavioral tests to further substantiate the link to MDD, such as the social interaction test or novelty-suppressed feeding test. This would provide a more comprehensive behavioral profile and strengthen the translational relevance of the findings.

The specificity of the observed behavioral effects on depression-like phenotypes needs more thorough investigation. Additional behavioral tests examining other domains (anxiety, cognition, social behavior) would help establish the specificity of the phenotype.

We thank the reviewer for this constructive feedback. In response, we have performed additional behavioral testing to complement our study. The results, now presented in

Figure S9g, provide important support for anxiety and social behavior. We agree with the reviewer that a more comprehensive assessment of cognitive function would be valuable. After careful consideration, we felt that the suggested test primarily overlaps with the cognitive domains already evaluated by our current battery, which includes the Novel Object Recognition (NOR), Fear Conditioning tests and Y maze.

Our data indicate that the delayed astrocyte development resulting from Egfr deletion selectively disrupts the integrative function of specific neural circuits, leading to behavioral despair and anhedonia in mice (as observed in SPT, TST, and FST)[35]. However, it does not impair brain regions responsible for anxiety-related behaviors (SI Ratio, NSFT, EPM)[36,37] or fundamental motivation (Splash test)[38]. This behavioral phenotype is consistent with cellular-level observations: the reduction in macroscopic neuronal connectivity (decreased processes) alongside preserved microscopic connectivity (unchanged spine density) suggests an altered foundational architecture of neural networks. We hypothesize that this alteration preferentially impairs higher-order brain regions, such as the medial prefrontal cortex (mPFC), in integrating reward (NAc/VTA) and stress signals, ultimately resulting in anhedonia and behavioral despair. In contrast, anxiety-like behaviors mediated by instinctive functions of specific nuclei such as the amygdala remain unaffected or are even alleviated (e.g., increased open-arm entries in EPM). Thus, findings from our mouse model suggest that astrocyte deficits during development may induce specific abnormalities in neural network architecture, thereby eliciting behavioral despair and anhedonia rather than anxiety-like phenotypes. This provides evidence supporting the role of astrocytes in the precise developmental regulation of emotional circuits[39,40].

Reference：

35 Russo, S. J. & Nestler, E. J. The brain reward circuitry in mood disorders. Nat Rev Neurosci 14, 609-625 (2013). https://doi.org/10.1038/nrn3381

36 Tye, K. M. et al. Amygdala circuitry mediating reversible and bidirectional control of anxiety. Nature 471, 358-362 (2011). https://doi.org/10.1038/nature09820

37 Calhoon, G. G. & Tye, K. M. Resolving the neural circuits of anxiety. Nat Neurosci 18, 1394-1404 (2015). https://doi.org/10.1038/nn.4101

38 Salamone, J. D. & Correa, M. The mysterious motivational functions of mesolimbic dopamine. Neuron 76, 470-485 (2012). https://doi.org/10.1016/j.neuron.2012.10.021

39 Cao, X. et al. Astrocyte-derived ATP modulates depressive-like behaviors. Nat Med 19, 773-777 (2013). https://doi.org/10.1038/nm.3162

40 Wang, Q., Jie, W., Liu, J. H., Yang, J. M. & Gao, T. M. An astroglial basis of major depressive disorder? An overview. Glia 65, 1227-1250 (2017). https://doi.org/10.1002/glia.23143

The authors should discuss the potential influence of other neural circuits (e.g., serotonergic or dopaminergic pathways) on the observed depressive-like behaviors, as these pathways are also implicated in MDD.

We thank you for this insightful suggestion and expand the disscussion relating to

serotonergic or dopaminergic pathways. We agree that serotonergic and dopaminergic pathways are critically involved in Major Depressive Disorder. To systematically address this, we examined our RNA-seq data for alterations in key genes within these pathways in the PFC of EGFR-cKO mice. Interestingly, while the expression of most serotonin and dopamine receptors (e.g., Htr1a-Htr7, Drd1-Drd5) and the dopamine transporter (Slc6a3) remained unchanged, we observed a significant upregulation of two key catabolic enzymes, MAOB and COMT. This specific finding suggests a potential disruption in monoamine metabolism rather than direct receptor signaling. Increased degradation of dopamine and norepinephrine by these enzymes may reduce monoamine availability in synaptic clefts, contributing to depressive-like behaviors independently of receptor alterations. Early astrocyte loss might indirectly impair monoaminergic circuits via disrupted glutamate homeostasis or neurotrophic support[41,42].

Reference：

41 Mahmoud, S., Gharagozloo, M., Simard, C. & Gris, D. Astrocytes Maintain Glutamate Homeostasis in the CNS by Controlling the Balance between Glutamate Uptake and Release. Cells 8 (2019). https://doi.org/10.3390/cells8020184

42 Sanacora, G., Treccani, G. & Popoli, M. Towards a glutamate hypothesis of depression: an emerging frontier of neuropsychopharmacology for mood disorders. Neuropharmacology 62, 63-77 (2012). https://doi.org/10.1016/j.neuropharm.2011.07.036

The Mendelian randomization analysis linking EPB41L2, PLXNA2, and PLXNA4 to MDD is compelling. However, the manuscript would benefit from a more detailed discussion of the potential clinical implications, including how these findings could inform therapeutic strategies for MDD. Additionally, a comparative analysis of the temporal dynamics of astrocyte-neuron crosstalk between mice and humans would enhance the translational impact of the study.

We thank the reviewer for their positive assessment of our Mendelian randomization analysis and for these exceptionally valuable suggestions to enhance the clinical and translational impact of our manuscript. We have carefully considered these points and have revised the Discussion accordingly to address them. As suggested, we have now expanded the Discussion section to include a dedicated discussion on the clinical implications of targeting the EPB41L2-PLXNA2/4 axis. "Mendelian randomization study identifies a causal link between EPB41L2, PLXNA2, PLXNA4, and MDD, with potential clinical implications. Firstly, as cell surface receptors, PLXNA2 and PLXNA4 represent 'druggable' targets. Therapeutic strategies could be explored to modulate their signaling activities, for instance, by developing monoclonal antibodies or soluble receptor fragments. Secondly, the genetic variants or expression levels of these genes could potentially serve as biomarkers for identifying an MDD subgroup characterized by dysregulated Semaphorin-Plexin signaling, paving the way for stratified medicine in psychiatry. While translating these findings into clinics requires overcoming significant challenges—such as

elucidating their precise roles in specific neural circuits and achieving brain-targeted drug delivery—our findings highlight novel and promising avenues for understanding MDD etiology and developing mechanism-based therapeutics”.

For the comparative analysis of temporal dynamics across species, we thank the reviewer for raising this insightful point. In response, we have incorporated a new paragraph in the Results section (line 128-135) that addresses the comparative temporal dynamics of astrocyte-neuron crosstalk in both mice and humans.

Minor Comments:
Expand on the discussion regarding the relevance of the EGFR-pERK-EPB41L2 axis in other neuropsychiatric conditions, considering its role in synaptic regulation.

We thank the reviewer for this valuable suggestion to explore the broader relevance of our findings. Indeed, the EGFR-pERK-EPB41L2 axis we identified represents a novel, cohesive pathway for astrocyte-neuron communication in dendritic regulation. While direct evidence for this exact axis in other neuropsychiatric conditions is still emerging, strong independent links exist for its core components.

Our study established that EGFR-pERK-EPB41L2 axis as a mechanism for astrocyte-neuron crosstalk in synaptic regulation. Although direct evidence for this specific axis in other neuropsychiatric conditions is sparse, its components are independently linked to key pathologies. ERK/MAPK dysregulation drives synaptic and inflammatory deficits in ASD[43] and OCD[44]. EPB41L2 dysfunction disrupts postsynaptic density integrity[45,46], and astrocytic EGFR in the amygdala gates fear behavior[47]. Aberrations in regulators of the ERK/MAPK signalling cascade contribute to a spectrum of neurodevelopmental and psychiatric disorders, including autism spectrum disorder (ASD), attention deficit hyperactivity disorder (ADHD), Fragile X syndrome, and Schizophrenia[48].
Thus, we propose that disturbances in this axis may represent a convergent pathway for synaptic dysfunction across disorders. Future studies should validate its role in ASD, OCD, and related conditions, leveraging our findings as a mechanistic foundation.

Reference:

43 Eissa, N. et al. Simultaneous Antagonism at H3R/D2R/D3R Reduces Autism-like Self-Grooming and Aggressive Behaviors by Mitigating MAPK Activation in Mice. Int J Mol Sci 24 (2022). https://doi.org/10.3390/ijms24010526

44 Ullrich, M. et al. OCD-like behavior is caused by dysfunction of thalamo-amygdala circuits and upregulated TrkB/ERK-MAPK signaling as a result of SPRED2 deficiency. Mol Psychiatry 23, 444-458 (2018). https://doi.org/10.1038/mp.2016.232

45 Scott, C., Keating, L., Bellamy, M. & Baines, A. J. Protein 4.1 in forebrain postsynaptic density preparations: enrichment of 4.1 gene products and detection of 4.1R binding proteins. Eur J Biochem 268, 1084-1094 (2001). https://doi.org/10.1046/j.1432-1327.2001.01968.x
46 Baines, A. J. & Bennett, V. Synapsin I is a spectrin-binding protein immunologically related to erythrocyte protein 4.1. Nature 315, 410-413 (1985). https://doi.org/10.1038/315410a0
47 Chung, E. N. et al. Psychedelic control of neuroimmune interactions governing fear. Nature 641, 1276-1286 (2025). https://doi.org/10.1038/s41586-025-08880-9
48 Iroegbu, J. D., Ijomone, O. K., Femi-Akinlosotu, O. M. & Ijomone, O. M. ERK/MAPK signalling in the developing brain: Perturbations and consequences. Neurosci Biobehav Rev 131, 792-805 (2021). https://doi.org/10.1016/j.neubiorev.2021.10.009

Some of the figure panels would benefit from additional quantification, particularly in Figure 2, where some morphological changes are shown qualitatively.

We thank the reviewer for this suggestion. We agree that quantitative analysis will strengthen the evidence for the morphological changes described in Figure 2 (current Figure 3). These new quantitative data have been added to the revised Figure 3, and the corresponding descriptions have been included in the Figure Legend and Methods section.

The authors should provide more detail about the age-specific differences in astrocyte-neuron communication patterns in the human data presented in Figure 1d.

Thank you for this insightful comment. We agree that a more detailed analysis of age-specific differences would strengthen our findings (please refer to Fig 1e and manuscript line 107-126).

Statistical analyses should be more clearly described in some figure legends, particularly regarding the number of biological replicates used (For example, Fig. 2a, 4c, and 4d).

We appreciate the reviewer's comment regarding statistical clarity. We have now revised all relevant figure legends to explicitly state the number of biological replicates (n) for each experiment. The specific 'n' for each panel and the statistical test used are now clearly indicated in each figure legend. We have also updated the supplementary table to further detail our statistical approach (Supplementary Table 5).

Additional controls for the specificity of the EGFR knockout effects on astrocytes

versus other cell types would be valuable.

We sincerely thank you for this insightful comment, which also raised by Reviewer 5. We acknowledge the well-known limitation of the hGFAP-Cre driver line. GFAP promoter activity can indeed be transiently present in neural stem cells (around E13.5), meaning recombination may occur beyond mature astrocytes. However, several lines of evidence support that the observed phenotypes are primarily attributable to Egfr deletion in the astrocyte lineage: Distinct Developmental Timing: Cortical radial glia generate pyramidal neurons from E13 to E16, and begin producing glial cells (including astrocytes) after E16[49]. Critically, Egfr expression in glial precursors initiates around E16, largely after the major period of neurogenesis. This temporal separation reduces the likelihood of direct Egfr deletion in neuronal progenitors significantly influencing pyramidal neuron development. In our Egfr CKO mice, we observed no significant alterations in the morphology of oligodendrocytes and astrocytes, nor in oligodendrocyte function such as myelination (Figure S10).

Consistent with our findings, Zhang et al. used clonal analysis to demonstrate that Egfr deletion markedly impairs astrocyte generation in the rostral brain, while the number of oligodendrocytes remains largely unaffected [50,51].
Collectively, these data strongly suggest that our model reliably reflects the function of EGFR in astrocyte development and its impact on pyramidal neurons.

Finally, we have revised the Discussion to explicitly acknowledge this limitation, stating:
"In addition, it is important to note that the hGFAP-cre driver used in this study is active in radial glia and their progeny. Although the combined evidence strongly points to an astrocyte-specific mechanism, future studies using astrocyte-specific Cre lines, when they become available, would be valuable to conclusively rule out potential contributions from other cell types."

Reference:

49 Li, X. et al. Decoding Cortical Glial Cell Development. Neurosci Bull 37, 440-460 (2021). https://doi.org/10.1007/s12264-021-00640-9

50 Zhang, X. et al. Clonal Analysis of Gliogenesis in the Cerebral Cortex Reveals Stochastic Expansion of Glia and Cell Autonomous Responses to Egfr Dosage. Cells 9 (2020). https://doi.org/10.3390/cells9122662

51 Zhang, X. et al. Bulk and mosaic deletions of Egfr reveal regionally defined gliogenesis in the developing mouse forebrain. iScience 26, 106242 (2023). https://doi.org/10.1016/j.isci.2023.106242

Fig. 2a, b: The criteria for morphological analysis of cells should be more explicitly defined in the Methods section.

We have provided a detailed description of the method as follows: To quantify dendritic arborization complexity, Sholl analysis was performed on reconstructed pyramidal neurons using *Fiji* with the Sholl Analysis plugin. The analysis commenced

at a radius of 1 µm from the soma and extended to 281 µm, with a radial increment of 20 µm. The primary metric quantified was the number of intersections at each concentric circle (the intersection count).

Given that the cells in Fig. 2a and 2b are too simple for Sholl analysis, we instead quantified the number of terminal points for each cell.

Reviewer #5 (Remarks to the Author):

The authors have investigated the consequences of conditional Egfr deletion. They find that this manipulation affects the maturation of astrocytes, cell marker expression, and cellular morphology. They also reveal that it leads to altered action potential firing and changes of synaptic transmission and neuronal morphology. The involvement of ERK-related signaling pathways and semaphorin 6A is investigated as well. There are concerns regarding, for instance, some experimental approaches and the presentation of the results.

1) Many experiments use the conditional deletion of Egfr using a commercially provided mouse line in which exon 2 of Egfr is 'floxed' and which was not fully characterized, crossed with a hGFAP-cre mouse line. It is unclear where the latter came from because a review is cited instead of an original paper characterizing the mouse line. This is problematic because it is well known that various cell types transiently show GFAP promoter activity and that, therefore, cre expression and cre-dependent recombination are not restricted to a single cell type (Casper and McCarthy, 2006). Without a careful characterization of the cell types from which Egfr is deleted and their respective percentages the data obtained with these animals is difficult to interpret.

We sincerely thank you for this insightful and critical comment. We agree that the specificity of genetic tools is paramount for the correct interpretation of data, and we appreciate the opportunity to address these concerns.

Regarding the citation of the hGFAP-Cre mouse line: The reviewer is absolutely correct that citing a review article for the source of a transgenic line is insufficient. We apologize for this oversight and have now updated the manuscript to include the primary reference for the generation and initial characterization of the hGFAP-Cre mouse line[52] in the Methods section.

We fully acknowledge the well-known limitation of the hGFAP-Cre driver line, as rightly noted by the reviewer. GFAP promoter activity can indeed be transiently present in neural stem cells (around E13.5), meaning recombination may occur beyond mature astrocytes. However, several lines of evidence support that the observed phenotypes are primarily attributable to Egfr deletion in the astrocyte lineage: Distinct Developmental Timing: Cortical radial glia generate pyramidal neurons from E13 to E16, and begin producing glial cells (including astrocytes) after

E16[49]. Critically, Egfr expression in glial precursors initiates around E16, largely after the major period of neurogenesis. This temporal separation reduces the likelihood of direct Egfr deletion in neuronal progenitors significantly influencing pyramidal neuron development. In our Egfr CKO mice, we observed no significant alterations in the morphology of oligodendrocytes and astrocytes, nor in oligodendrocyte function such as myelination (Figure S10).

Consistent with our findings, Zhang et al. used clonal analysis to demonstrate that Egfr deletion markedly impairs astrocyte generation in the rostral brain, while the number of oligodendrocytes remains largely unaffected[50,51]
Collectively, these data strongly suggest that our model reliably reflects the function of EGFR in astrocyte development and its impact on pyramidal neurons.

Finally, we have revised the Discussion to explicitly acknowledge this limitation, stating:
"In addition, it is important to note that the hGFAP-cre driver used in this study is active in radial glia and their progeny. Although the combined evidence strongly points to an astrocyte-specific mechanism, future studies using astrocyte-specific Cre lines, when they become available, would be valuable to conclusively rule out potential contributions from other cell types."

Regarding the citation of the Egfr^{flox} mouse line: We thank the reviewer for this comment. The floxed-Egfr mice used in our study, which targets exon 2, are widely used and validated in the field [53,54]. While a full characterization was not performed, we provide direct evidence of successful knockout: our RNA-seq data confirms exon 2 deletion, and this is followed by a reduction in downstream MAPK/ERK signaling (Fig. 3g, S2b). Furthermore, we have directly compared this exon 2 floxed model (which has the added benefit of expressing a traceable truncated protein) with an exon 3 floxed model [50,51,55]. The fact that both independent mouse lines exhibit identical cytological and behavioral phenotypes provides strong functional validation that our key findings are a direct result of Egfr deletion.

Zhuo, L., Theis, M., Alvarez-Maya, I., Brenner, M., Willecke, K., & Messing, A. (2001). hGFAP-cre transgenic mice for manipulation of glial and neuronal function in vivo. Genesis (New York, N.Y. : 2000), 31(2), 85–94.

Reference:

49 Li, X. et al. Decoding Cortical Glial Cell Development. Neurosci Bull 37, 440-460 (2021). https://doi.org/10.1007/s12264-021-00640-9

50 Zhang, X. et al. Clonal Analysis of Gliogenesis in the Cerebral Cortex Reveals Stochastic Expansion of Glia and Cell Autonomous Responses to Egfr Dosage. Cells 9 (2020). https://doi.org/10.3390/cells9122662

51 Zhang, X. et al. Bulk and mosaic deletions of Egfr reveal regionally defined gliogenesis in the developing mouse forebrain. iScience 26, 106242 (2023).

https://doi.org/10.1016/j.isci.2023.106242
52 Zhuo, L. et al. hGFAP-cre transgenic mice for manipulation of glial and neuronal function in vivo. Genesis 31, 85-94 (2001). https://doi.org/10.1002/gene.10008
53 Liu, B. et al. CRISPR-mediated ablation of overexpressed EGFR in combination with sunitinib significantly suppresses renal cell carcinoma proliferation. PLoS One 15, e0232985 (2020). https://doi.org/10.1371/journal.pone.0232985
54 Maklad, A. et al. The EGFR is required for proper innervation to the skin. J Invest Dermatol 129, 690-698 (2009). https://doi.org/10.1038/jid.2008.281
55 Lee, T. C. & Threadgill, D. W. Generation and validation of mice carrying a conditional allele of the epidermal growth factor receptor. Genesis 47, 85-92 (2009). https://doi.org/10.1002/dvg.20464

2) The manuscript is difficult to read and understand because terms are not well explained. It seems that 'crosstalk' refers to all potential interactions that astrocytes and neurons are capable of, which includes everything from the astrocytic role in neuronal maturation and synaptogenesis to their role as signaling partners defining synaptic function, plasticity and complex behaviors. This seems too broad and unspecific to be useful given the current knowledge of the many ways that astrocytes contribute to brain function. The same applies to the term 'communication', which is also used in a very broad fashion. Inferring such communication from single-cell RNA sequencing data (e.g. lines 84-85) seems to ignore most of the complexity of cell-to-cell communication in the brain.

We sincerely thank the reviewer for highlighting this important point regarding the need for experimental validation of cell–cell interactions. We fully agree that functional studies would provide valuable future insights. In the present work, we aimed to leverage the strengths of high-throughput transcriptomics and state-of-the-art computational algorithms, which represent an established and powerful paradigm for mapping putative cellular communication events[4]. Cell-cell interactions (CCIs) constitute the fundamental basis of multicellular life, enabling cellular populations to coordinate and execute complex biological functions[1-3]. Specifically, CCIs involve processes wherein cells activate signaling pathways in neighboring cells through specific molecules—such as ligands, receptors, and functional proteins—as well as membrane-associated structures like the extracellular matrix, membrane protrusions, and extracellular vesicles. These interactions regulate gene expression and drive corresponding cellular functions. Among various CCI mechanisms, ligand-receptor interactions (LRIs) represent the predominant mode of regulation. In LRIs, a ligand secreted by one cell binds to its cognate receptor on another, initiating intercellular signal transduction[4]. This form of LRI-mediated communication, also referred to as cell-cell communication (CCC), plays a pivotal role in both physiological and pathological processes. For instance, during neural development, microglia promote the proliferation of GABAergic neurons by secreting IGF1, which acts on

neuronal IGF receptors (IGFR)[5]. Similarly, cortical astrocytes regulate the formation of synapse-specific connections through neuronal Sonic hedgehog (Shh) signaling via the Ptch1 receptor[6]. Furthermore, RABID-seq has identified Sema4D-PlexinB1, Sema4D-PlexinB2, and Ephrin-B3–EphB3 as key ligand-receptor pairs mediating microglia-astrocyte interactions[7]. These interactions not only contribute to the pathogenesis of central nervous system disorders but also represent promising therapeutic targets for neurodegenerative diseases. In summary, CCIs exert precise and extensive control over neurodevelopment, inflammatory modulation, synaptic plasticity, and emotional behaviors through intricate ligand-receptor signaling networks in both health and disease.
In our study, we propose that Sema6a–Plxna2/4 ligand-receptor pairs play critical roles in the development of pyramidal neurons. In accordance with your and the other reviewers' insightful suggestions, we are conducting additional experiments to further validate the functions of this ligand-receptor pair (please refer to the manuscript).

References :

1 Zhou, X. et al. Circuit Design Features of a Stable Two-Cell System. Cell 172, 744-757.e717 (2018). https://doi.org/10.1016/j.cell.2018.01.015

2 Rouault, H. & Hakim, V. Different cell fates from cell-cell interactions: core architectures of two-cell bistable networks. Biophys J 102, 417-426 (2012). https://doi.org/10.1016/j.bpj.2011.11.4022

3 Bonnans, C., Chou, J. & Werb, Z. Remodelling the extracellular matrix in development and disease. Nat Rev Mol Cell Biol 15, 786-801 (2014). https://doi.org/10.1038/nrm3904

4 Armingol, E., Baghdassarian, H. M. & Lewis, N. E. The diversification of methods for studying cell-cell interactions and communication. Nat Rev Genet 25, 381-400 (2024). https://doi.org/10.1038/s41576-023-00685-8

5 Yu, D. et al. Microglia regulate GABAergic neurogenesis in prenatal human brain through IGF1. Nature (2025). https://doi.org/10.1038/s41586-025-09362-8

6 Xie, Y. et al. Astrocyte-neuron crosstalk through Hedgehog signaling mediates cortical synapse development. Cell Rep 38, 110416 (2022). https://doi.org/10.1016/j.celrep.2022.110416

7 Clark, I. C. et al. Barcoded viral tracing of single-cell interactions in central nervous system inflammation. Science 372 (2021). https://doi.org/10.1126/science.abf1230

3) The data is incompletely described. The number of experiments, mice, samples and cells are mostly missing. There is insufficient information given about statistical tests and their results in the manuscript. There are incompletely labeled figures and unexplained abbreviations. For instance, in the experiments with Sema6a expression in Fig. 4d, it is unclear how often these experiments were performed, whether there are significant differences, and the x-axis label is missing. Consequently, it is often not possible to follow the conclusions presented by the authors.

Thank you for your feedback. We apologize for these oversights. We have now revised the manuscript to include all missing experimental details, sample sizes, statistical tests, and complete figure labels. A full description of the statistical methods and results has been added to the Methods section and a new Supplementary Table (Supp Tab 5). We believe the revised manuscript now allows for a clear evaluation of the results.

Casper KB, McCarthy KD (2006) GFAP-positive progenitor cells produce neurons and oligodendrocytes throughout the CNS. Mol Cell Neurosci 31:676–684.

地址：上海市东安路131号
ADDRESS No.131 DONGAN ROAD SHANGHAI
电话(TEL) : 021-54237641 邮编 (CODE): 200032
iobs@fudan.edu.cn

Jan. 10, 2026

Response to Reviewers' Comments

We sincerely thank all reviewers for their careful evaluation and constructive comments. We appreciate the time and effort invested in the review process, which has substantially improved the clarity, rigor, and overall impact of our manuscript. Below, we address each comment in detail and indicate how the manuscript has been revised accordingly. Your comments given verbatim in black text, and our responses in blue.

Reviewer #1 (Remarks to the Author):

Overall the authors have addressed the reviewers' concerns. However, in a few instances, inclusions of the points made to reviewers should be included in the manuscript and aren't (in general, for the future, recommend indicating where in the manuscript different aspects of the revisions are included). I have included a non-exhaustive list, but encourage the authors to double check the reviewers' comments and make sure that the responses, when able to be, are updated in the manuscript text.

Response: We thank you for noting that some points addressed in the responses were not sufficiently reflected in the manuscript text. We apologize for this oversight. In the revised version, we have carefully ensured that all substantive responses are now explicitly incorporated into the manuscript, with corresponding revisions clearly indicated in the appropriate sections.

Reviewer 1

Point 1. Thank you to the authors for their summary of crosstalk. However, as multiple reviewers mentioned, there's a conflation of cell-cell interactions broadly versus one specific ligand-receptor interaction. Encourage the authors to be specific in their title and their conclusions.

Response: We agree that greater specificity regarding the molecular mechanisms underlying astrocyte–neuron interactions substantially strengthen the manuscript. Accordingly, we have revised the title to explicitly reflect the signaling axis investigated: **Delayed astrocyte development disrupts Sema6a–Plxna2/4 signaling and leads to depressive-like behavior.**

In parallel, we have revised the Results and Discussion sections to frame our conclusions around defined ligand–receptor interactions and signaling pathways, rather than broadly referring to astrocyte–neuron crosstalk. These changes improve conceptual precision and align the conclusions more closely with the presented data.

In addition, we have expanded the Introduction to include a concise discussion of cell–cell communication and astrocyte–neuron signaling, providing appropriate context for the reader (Page 2, Line 53-60)

Point 2, are the violin plots in the updated manuscript?

地址：上海市东安路131号
ADDRESS No.131 DONGAN ROAD SHANGHAI
电话(TEL) : 021-54237641 邮编 (CODE): 200032
iobs@fudan.edu.cn

Response: Yes. We thank you for the confirmation. The violin plot has been included in the revised manuscript (please see Supplementary Fig. 12a).

Point 7 about references was meant to indicate to include them in the text.

Response: Thank you for the clarification. The relevant references have now been inserted into the main text.

Point 8 Cued and contextual fear conditioning: The assessment of darting behavior has now been explicitly described in the Methods section (Page 29, Line1121-1124).

Points 10–11 Observations regarding elevated plus maze performance and repetitive behaviors have now been incorporated into the Results section (Page 8-9, Line324-330).

Points 14, 15, 19. Please make sure this information is included in the text.

Response: As requested, the relevant information has been incorporated into the manuscript to improve clarity (Page 26, Line992-1006).

In addition to those updates, I think the authors could include a bit more of a link between their original findings, which are outside of the regions they cite as being important for behaviours (Reviewer 1 Point 16). Can they include any IHC of the Pre- or Infra-limbic cortices with regards the altered astrocyte organization (Figure 2,3, Supplemental Figure 1-3, 5, 8, etc)? If not, this should be discussed in the manuscript.

Response: We thank you for this insightful suggestion to better connect our findings with prefrontal circuitry, particularly the prelimbic (PL) and infralimbic (IL) cortices, which are relevant to the behavioral phenotypes examined. In response, we performed additional analyses and have included new data from the prefrontal cortex in the revised manuscript (Supplementary Fig 3).

While a systematic IHC analysis across all previously examined regions within the PL/IL cortices was beyond the scope of the current revision, the newly added data provide an initial link between altered astrocyte organization and these behaviorally relevant regions. In addition, as suggested by the reviewer, we have expanded the Discussion to explicitly address this issue. A dedicated paragraph now discusses how the astrocytic deficits identified in our study may relate to the established roles of PL/IL circuits in mood regulation (Page 12, Line446-453; Page 7, Line256-258; Page 8, Line319-324).

Missing references for sentences on lines:
60-67
70-74

125-126
133-135
207-210
257-259
297-302
409-413
448-462

Response: Thank you for pointing out these omissions. Appropriate references have now been added at the indicated locations.

Reviewer #3 (Remarks to the Author):

I co-reviewed this manuscript with one of the reviewers who provided the listed reports. This is part of the Nature Communications initiative to facilitate training in peer review and to provide appropriate recognition for Early Career Researchers who co-review manuscripts.

Response: We acknowledge the Nature Communications co-review initiative and thank both reviewers, including the early-career researcher, for their contributions. All points raised in the reports have been fully addressed in the revised manuscript.

Reviewer #4 (Remarks to the Author):

This revision presents a solid and comprehensive mechanistic framework linking astrocyte developmental timing with neuronal circuit maturation and mood regulation. The addition of long-term behavioral and electrophysiological validation greatly enhances the manuscript's impact and suitability for publication.

Response: We appreciate your positive assessment of the revised manuscript and are grateful for the constructive feedback. We are pleased that the added long-term behavioral and electrophysiological data enhance the mechanistic framework and overall impact of the study.

Reviewer #5 (Remarks to the Author):

The authors have not convincingly addressed my previous comments. Regarding the first comment, for example, they now cite the paper describing the hGFAP-cre mouse line (Zhuo et al., 2001). This study demonstrates that cre expression in this line begins around embryonic day 13 in cells that later give rise to neurons, astrocytes, and other cell types. Both this and other work (Casper and McCarthy, 2006; Zhuo et al., 2001) further show that cre expression is heterogeneous, with reporter activity observed only in subpopulations of certain cell types. From this relatively poorly defined population of cells, Egfr is deleted before and during their differentiation. It is also deleted from cells within the postnatal neurogenic niches that exhibit GFAP promoter activity. It remains unclear to this reviewer how solid conclusions can be drawn from this rather undefined experimental manipulation.

Response: We thank you for raising these important concerns, which are critical for proper

interpretation of our findings. To address them, we performed additional analyses and revised the manuscript to clarify the temporal and cellular context of EGFR deletion.

1. **Temporal onset and specificity of hGFAP-Cre–mediated EGFR recombination**

Previous studies and our own analyses indicate that hGFAP-Cre robustly labels the majority of cortical radial glial cells and their progeny, with recombination detectable from approximately embryonic day E13 (**Fig. 1a**).

To further ensure specificity, we generated an *Egfr* Exon3 conditional knockout allele, which abolishes EGFR protein expression without producing truncated products. Immunohistochemical and biochemical analyses confirmed efficient cortical deletion of EGFR, with residual expression restricted to regions exhibiting minimal hGFAP-Cre activity (**Fig. 1b, c**).

2. **Endogenous EGFR expression during cortical development**

Using immunostaining, RNA in situ hybridization, and single-cell RNA sequencing, we examined endogenous EGFR expression across developmental stages. These analyses consistently show minimal EGFR expression before E16, followed by a marked increase from E16–E18, primarily in astrocyte-lineage progenitors and immature astrocytes. Postnatally, EGFR expression is largely restricted to astrocytes, with negligible expression in neuronal or oligodendrocyte lineages (**Figs. 2–3**).

3. **Assessing effects on non-astrocytic lineages**

Quantitative analyses of neuronal and oligodendrocyte populations in adult mutant mice revealed no significant differences compared with controls, while astrocyte number and maturation were altered (**Fig. 4**). EGFR expression was not detected in the adult ventricular zone, and no change in olfactory bulb interneuron numbers was observed, arguing against a major contribution of altered adult neurogenesis.

4. **Independent validation using an inducible astrocyte-specific Cre line**

To further isolate the contribution of astrocytic EGFR during postnatal development, we employed Aldh1l1-CreERT2 to induce recombination after the completion of cortical neurogenesis. This approach recapitulated key phenotypes observed in the hGFAP-Cre model, including altered pyramidal neuron morphology and depressive-like behaviors (**Fig. 5**), providing independent support for an astrocyte-associated mechanism.

5. **Interpretation of recombination timing and phenotype attribution**

Taken together, these data clarify the temporal and cellular context of EGFR deletion in the hGFAP-Cre; *Egfr* model. Although recombination initiates during early embryogenesis, endogenous EGFR expression emerges predominantly after the peak of cortical neurogenesis and is enriched in

地址：上海市东安路131号
ADDRESS No.131 DONGAN ROAD SHANGHAI
电话(TEL) : 021-54237641 邮编 (CODE): 200032
iobs@fudan.edu.cn

astrocyte-lineage cells. The convergence of developmental expression analyses, lineage-specific assessments, and inducible-Cre validation supports an interpretation in which the observed adult phenotypes are closely associated with disrupted EGFR-dependent astrocyte development.

Figure 1.

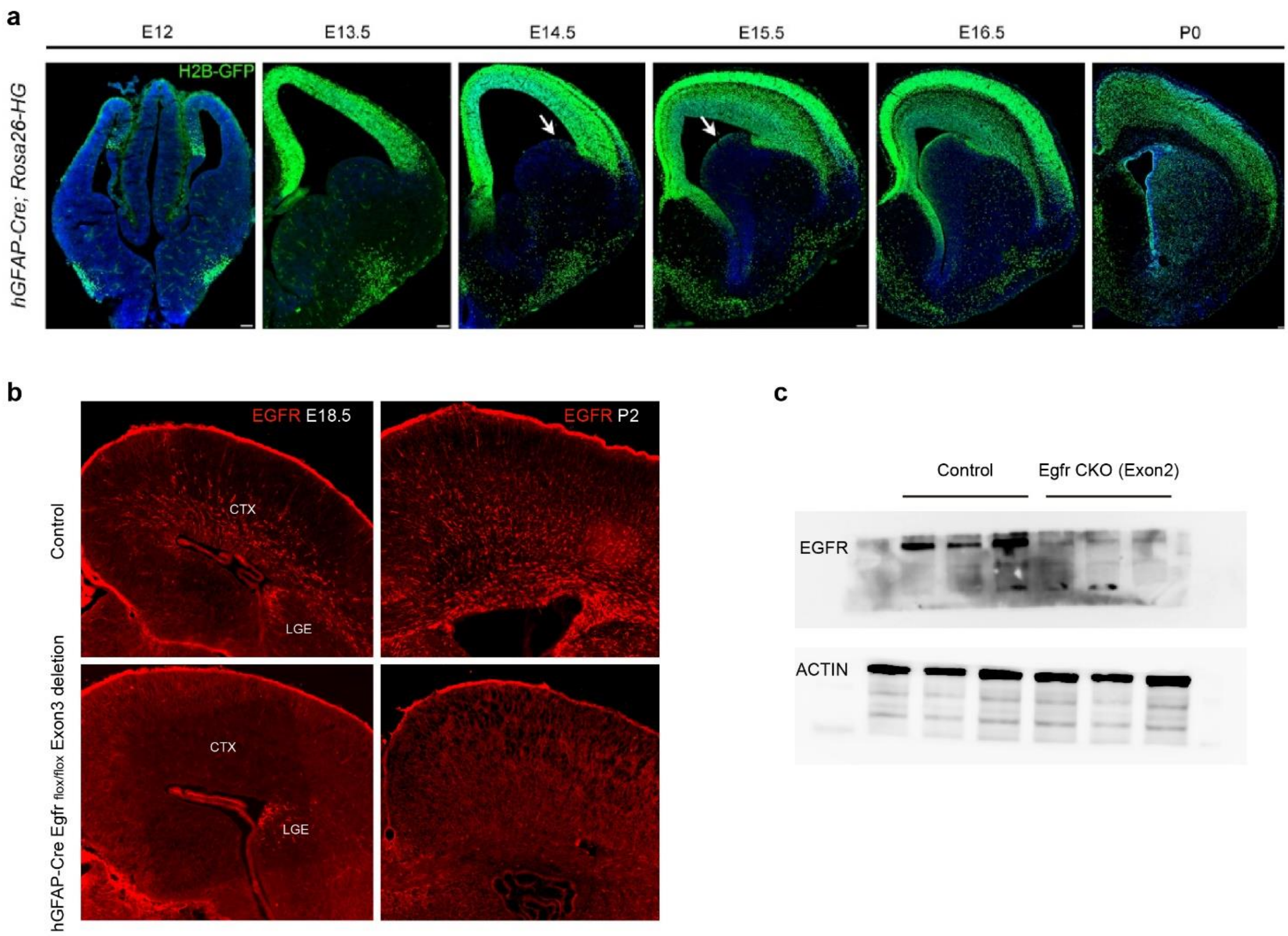

Figure 1. Efficient cortical deletion of EGFR using hGFAP-Cre.

a. hGFAP-Cre mice were crossed with Rosa26-HG (H2b) reporter mice to label radial glial lineages, resulting in widespread GFP expression in cortical radial glial cells and their progeny (Li Z. *et al.* Protein Cell, 2024).

b. Immunostaining for EGFR in Egfr $^{flox/flox}$; hGFAP-Cre mice at embryonic day 18 (E18) and postnatal day 2 (P2) shows a marked reduction of EGFR signal throughout the cortex compared with littermate controls. Residual EGFR expression is observed in the lateral ganglionic eminence (LGE), a region in which hGFAP-Cre activity is minimal (arrows; see panel a)

c. Western blot analysis of cortical tissue at postnatal day 14 (P14) shows a strong reduction of EGFR protein levels in Egfr $^{flox/flox}$; hGFAP-Cre mice relative to controls.

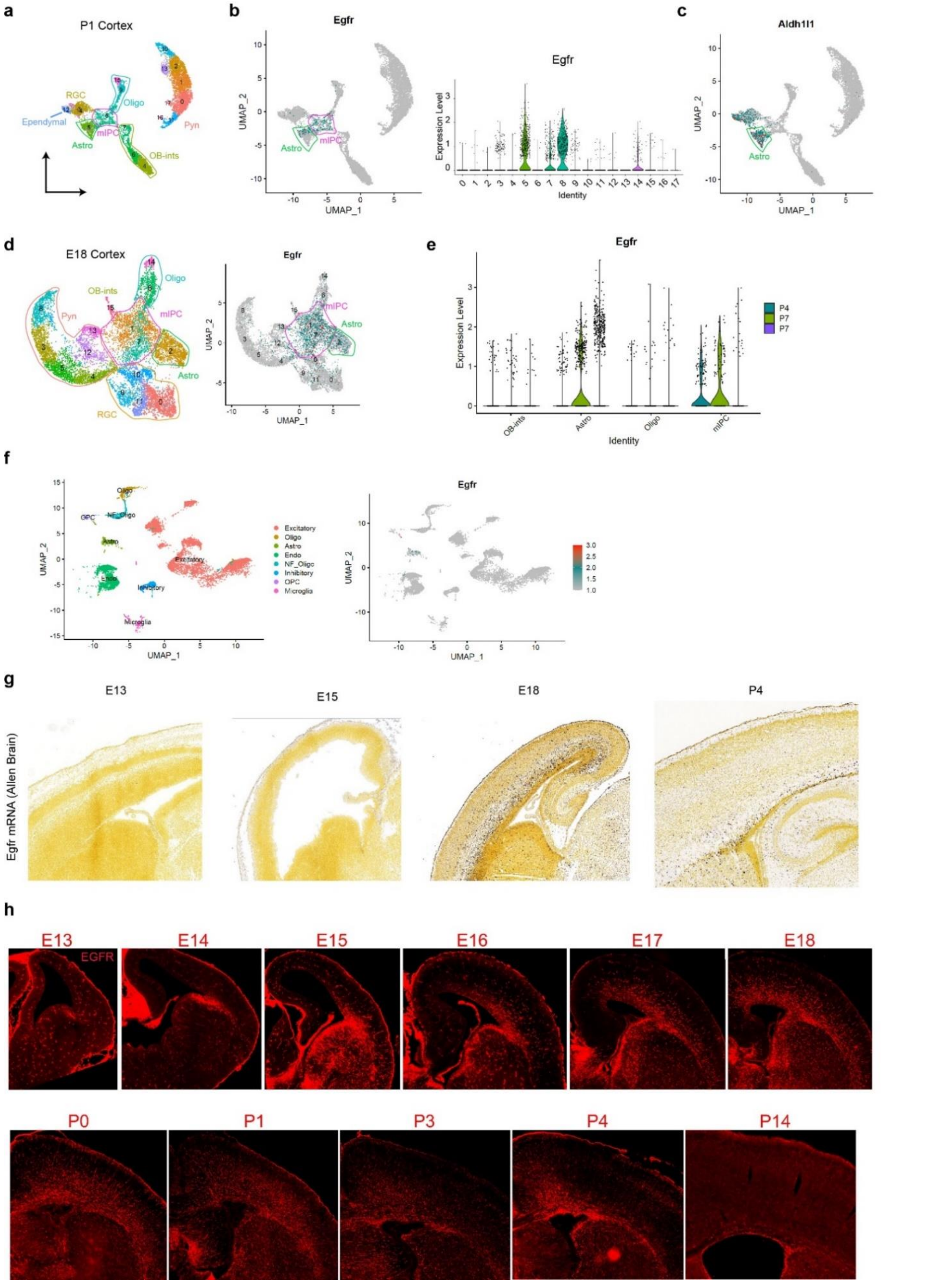

Figure 2. **Developmental expression of *Egfr* in cortical astrocyte-lineage cells revealed by single-cell**

地址：上海市东安路131号
ADDRESS No.131 DONGAN ROAD SHANGHAI
电话(TEL) : 021-54237641 邮编 (CODE): 200032
iobs@fudan.edu.cn

transcriptomic and histological analyses.

a. UMAP projection of single-cell RNA-sequencing data showing seven major cortical cell types.

b. UMAP projection and corresponding violin plot illustrating *Egfr* expression across cortical cell populations.

c. UMAP projection showing expression of *Aldh1l1*, indicating astrocyte-lineage cells.

d. UMAP projection highlighting *Egfr* expression in multipotent intermediate progenitor cells (MIPCs) and astrocyte-lineage cells in the E18 cortex.

e. Violin plots showing *Egfr* expression in astrocytes and MIPCs at postnatal days P4 and P7.

f. UMAP projection showing reduced *Egfr* expression at P21.

g. RNA in situ hybridization illustrating *Egfr* mRNA expression in the cortex at E18 and P4, but not at E13 or E15 (data from the Allen Brain Atlas).

h. Immunostaining showing the spatial and temporal expression pattern of EGFR in the cortex from E13 to P14.

Figure 3.

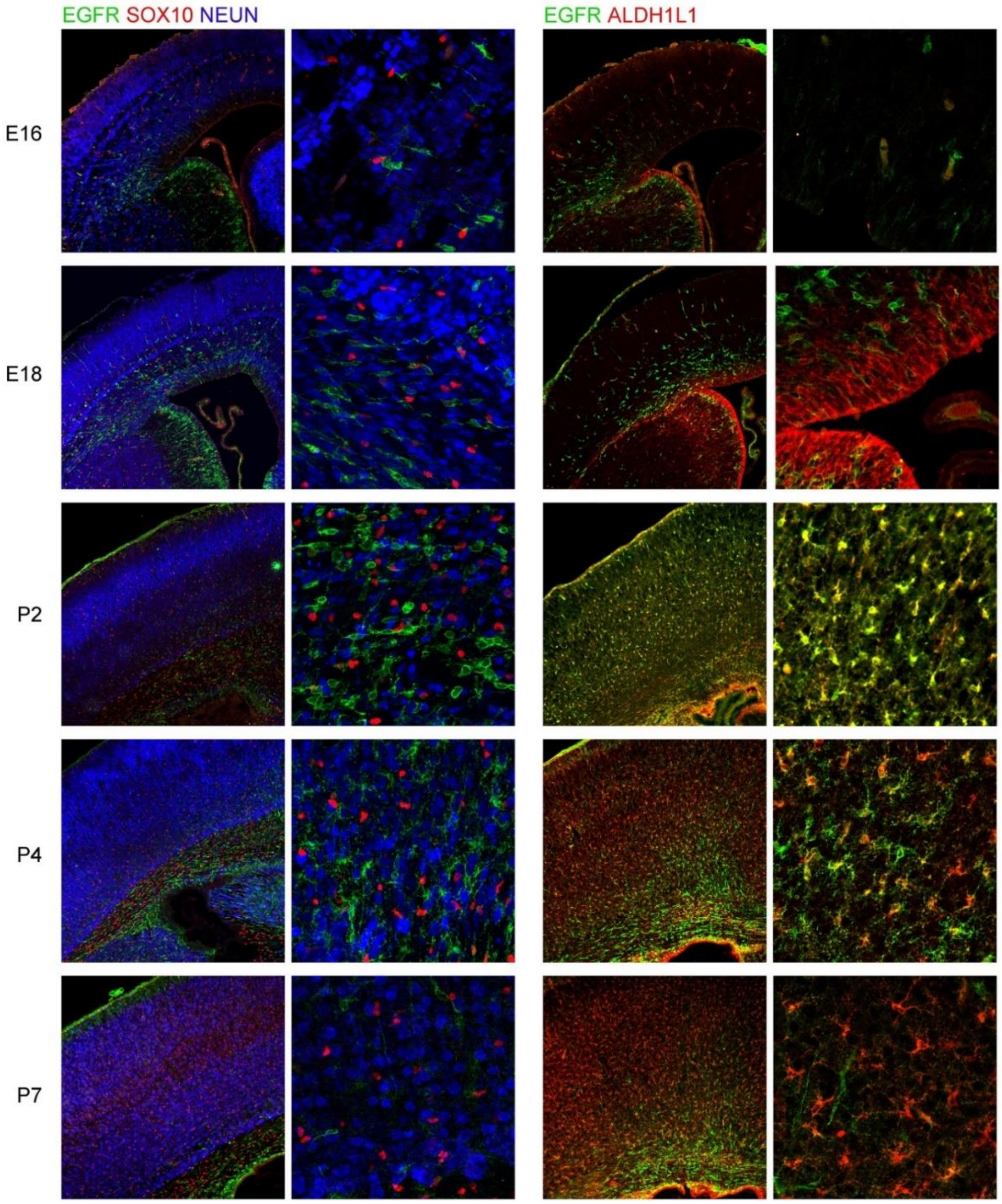

Figure 3. EGFR expression preferentially associates with astrocytes rather than neuronal or oligodendrocyte lineages.

Figure 4.

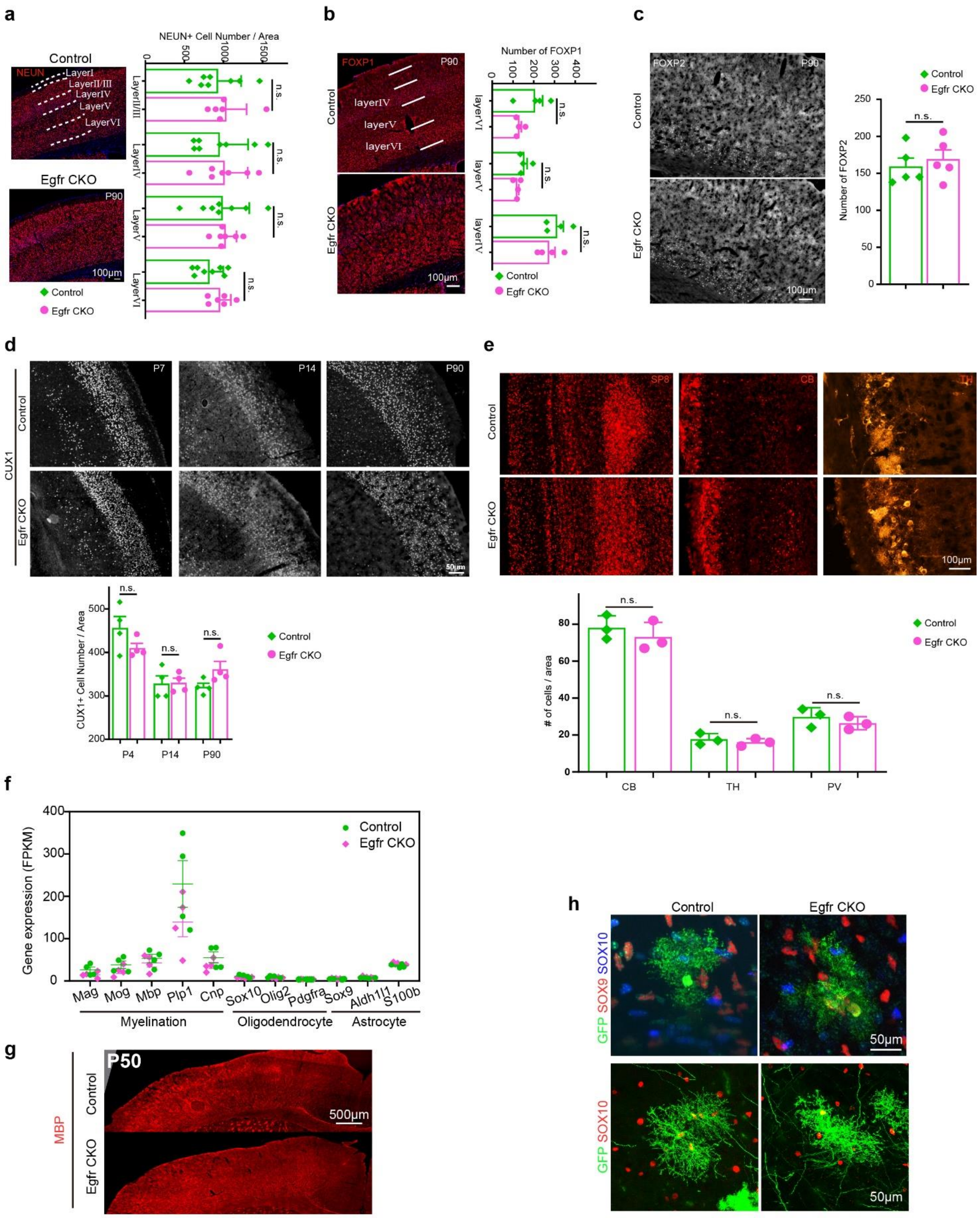

Figure 4. **Neuronal development is largely preserved following cortical *Egfr* deletion**.

a-d. Representative images and quantification of NEUN-, CUX1-, FOXP1-, and FOXP2-positive cells in the cortex of *Egfr* conditional knockout (CKO) mice and control littermates (n = 4–8 mice per group; each data

地址：上海市东安路131号
ADDRESS No.131 DONGAN ROAD SHANGHAI
电话(TEL) : 021-54237641 邮编 (CODE): 200032
iobs@fudan.edu.cn

point represents an individual mouse).

e. Immunostaining of olfactory bulb interneuron markers and quantification of the corresponding cell numbers (n = 3 mice per group).

f. Dot plot showing the expression of genes associated with myelination, oligodendrocytes, and astrocytes.

g. Immunostaining for myelin basic protein (MBP) in the cortex of *Egfr* CKO mice and controls at P50.

h. In utero electroporation of a pCAG-GFP plasmid into the cortex of *Egfr* CKO and control mice at P0, followed by analysis at P21. Representative images showing the morphology of astrocytes and oligodendrocytes.

Figure 5.

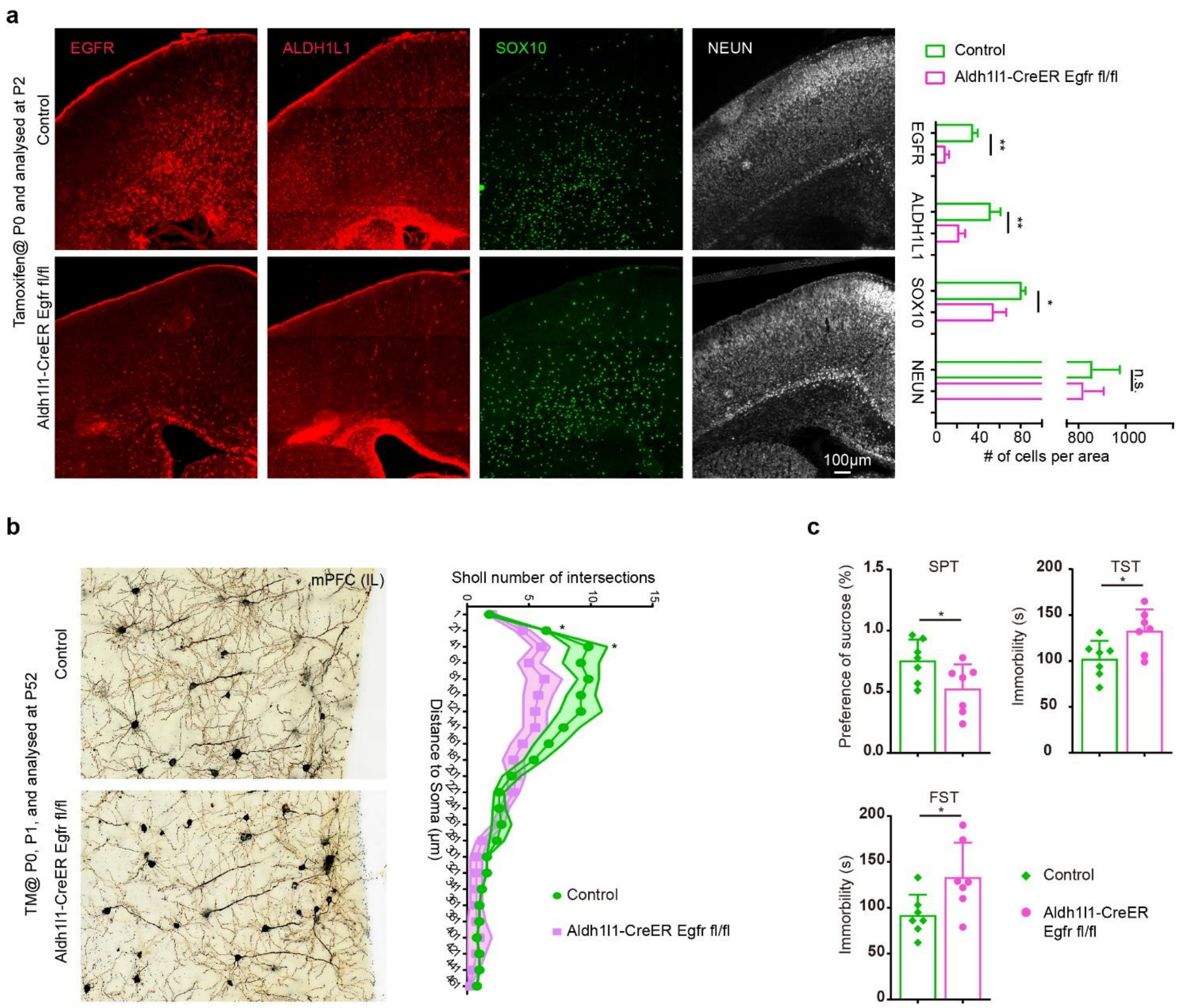

Figure 5. Astrocyte-specific deletion of *Egfr* is associated with altered neuronal morphology and behavioral phenotypes

a. Schematic of the astrocyte-specific conditional knockout strategy. *Egfr*$^{flox/flox}$ mice were crossed with *Aldh1l1*-CreERT2 mice, and tamoxifen was administered at postnatal days P0 and P1. Cortical tissue was analyzed at P2 to assess recombination efficiency

地址：上海市东安路131号
ADDRESS No.131 DONGAN ROAD SHANGHAI
电话(TEL) : 021-54237641 邮编 (CODE): 200032
iobs@fudan.edu.cn

b. Representative images of Golgi-stained neurons in the infralimbic cortex (IL) of Aldh1l1-CreER; Egfr $^{flox/flox}$ mice and their littermate controls at P52.

c. Behavioral assessment of *Aldh1l1*-CreERT2; *Egfr* $^{flox/flox}$ mice and littermate controls across a battery of depression-related behavioral tests at P60. For behavioral experiments, tamoxifen (50 mg/kg) was administered intraperitoneally at embryonic days E16, E17, and E18 to induce recombination

Finally, the hGFAP-Cre line remains one of the most widely used and genetically validated tools for studying astrocyte function in vivo. Its well-established utility is demonstrated by numerous high-impact studies. For instance, seminal work relying on this model has shown that astrocyte-specific deletion of Pten induces cellular hypertrophy, while knockout of Glutamine Synthetase results in neonatal lethality. Such findings have provided foundational insights into the critical roles of astrocytes in brain development, homeostasis, and neurological disorders. (He Y, et al. Glia. 2010. PMID: 20140959；Nakase T, et al. Cell Commun Adhes. 2003. PMID: 14681050；Li T, et al. Glia. 2020. PMID: 31868275；Malik S, et al. Front Mol Neurosci. 2017. PMID: 29259541；Howng SY, et al. PLoS One. 2015. PMID: 25962146 ；Ge S, et al. J Mol Neurosci. 2009. PMID: 19340610；Xia M, et al. Theranostics. 2025. PMID: 40213654 ；S Iacobas, et al. Brain Res. PMID: 22771707; Xin-Chun Ye, et al. Stroke. 2018. PMID: 29212737; Fujita M, et al. Biol Pharm Bull. 2018. PMID: 29709916; Bajenaru ML, et al. Mol Cell Biol. 2002. PMID: 12077339). Our study follows this same established paradigm. The phenotypes we observed occurred in adulthood and were supported by additional experimental validation, allowing us to confidently attribute them to EGFR loss in astrocytes. Therefore, we believe our conclusions, based on this widely accepted model, are robust and valid.

We hope that these revisions and additional analyses adequately address your concerns. We believe that the revised manuscript now presents a clearer and more rigorous interpretation of the data, and we are grateful for the thoughtful feedback that has strengthened this work.

REVIEWERS' COMMENTS

Reviewer #1 (Remarks to the Author):

The revision has addressed all our concerns

Response: We are very pleased to receive your positive evaluation of our revised manuscript and thank you for the thoughtful comments. We also wish to express our sincere gratitude for the time and guidance you have devoted to enhancing our work.

Reviewer #3 (Remarks to the Author):

I co-reviewed this manuscript with one of the reviewers who provided the listed reports. This is part of the Nature Communications initiative to facilitate training in peer review and to provide appropriate recognition for Early Career Researchers who co-review manuscripts.

Response: We acknowledge the Nature Communications co-review initiative and thank both reviewers, including the early-career researcher, for their contributions. All points raised in the reports have been fully addressed in the revised manuscript. We also extend our sincere thanks to you for your time and guidance throughout the review process.
