# Supplementary material for: Delayed astrocyte development impairs Sema6a-Plxna2/4-mediated astrocyte-neuron crosstalk and causes depressive-like behavior
Source: Nat Commun. 2026 Jul 18;17:8800. doi: 10.1038/s41467-026-75697-z (PMC13494039; doi:10.1038/s41467-026-75697-z)
Supplement: Supplementary file 2 [file 41467_2026_75697_MOESM1_ESM.pdf]

Delayed Astrocyte Development Impairs Sema6a -Plxna2/4-Mediated Astrocyte-Neuron Crosstalk and Causes Depressive-like Behavior

Xin Jiang[1,*], Yanqing Qi[1,*], Lin Yang[1, 2], Feihong Yang[1], Rongliang Guo[1, 3], Liang Li[1], Kun Wang[1], Lichen Sun[4], Dan Dai[5], Hanchen Liu[5], Yanjing Gao[1], Mengge Sun[1], Xiaolei Song[1], Zhuangzhi Zhang[1], Zhejun Xu[1], Bin Luo[6], Yunli Xie[1], Zhengang Yang[1], Miao He[1, ✉] & Dashi Qi[4, ✉] & Xiaodan Zhang[1, 7, ✉] & Guoping Liu[1, 5, 8, ✉]

[1]State Key Laboratory of Brain Function and Disorders, Ministry of Education Frontiers Center for Brain Science, Institutes of Brain Science, Department of Rehabilitation, Huashan Hospital, Fudan University, Shanghai, China.

[2] Department of Neuroscience and Kavli Institute for Neuroscience, Yale School of Medicine, New Haven, CT, USA.

[3] Department of Central Laboratory, Affiliated Hospital of Hebei University of Engineering, Handan, Hebei, China.

[4] Center for Clinical Research and Translational Medicine, Yangpu Hospital, School of Medicine, Tongji University, Shanghai 200090, P.R. China.

[5] Neurovascular Center, Changhai Hospital, Institute of Neuroscience, Key Laboratory of Molecular Neurobiology, NMU, Shanghai, China.

[6] Chinese Institutes for Medical Research (CIMR), Beijing, China.

[7] Obstetrics & Gynecology Hospital of Fudan University, Shanghai Key Lab of Reproduction and Development, Shanghai Key Lab of Female Reproductive Endocrine Related Diseases, 200433, Shanghai, China.

[8] State Key Laboratory of Neurology and Oncology Drug Development, Nanjing, China.

[*]These authors contributed equally: Xin Jiang, Yanqing Qi.

✉e-mail: hem@fudan.edu.cn; qidashi@fudan.edu.cn; xdzhang14@fudan.edu.cn; Gpliu@fudan.edu.cn

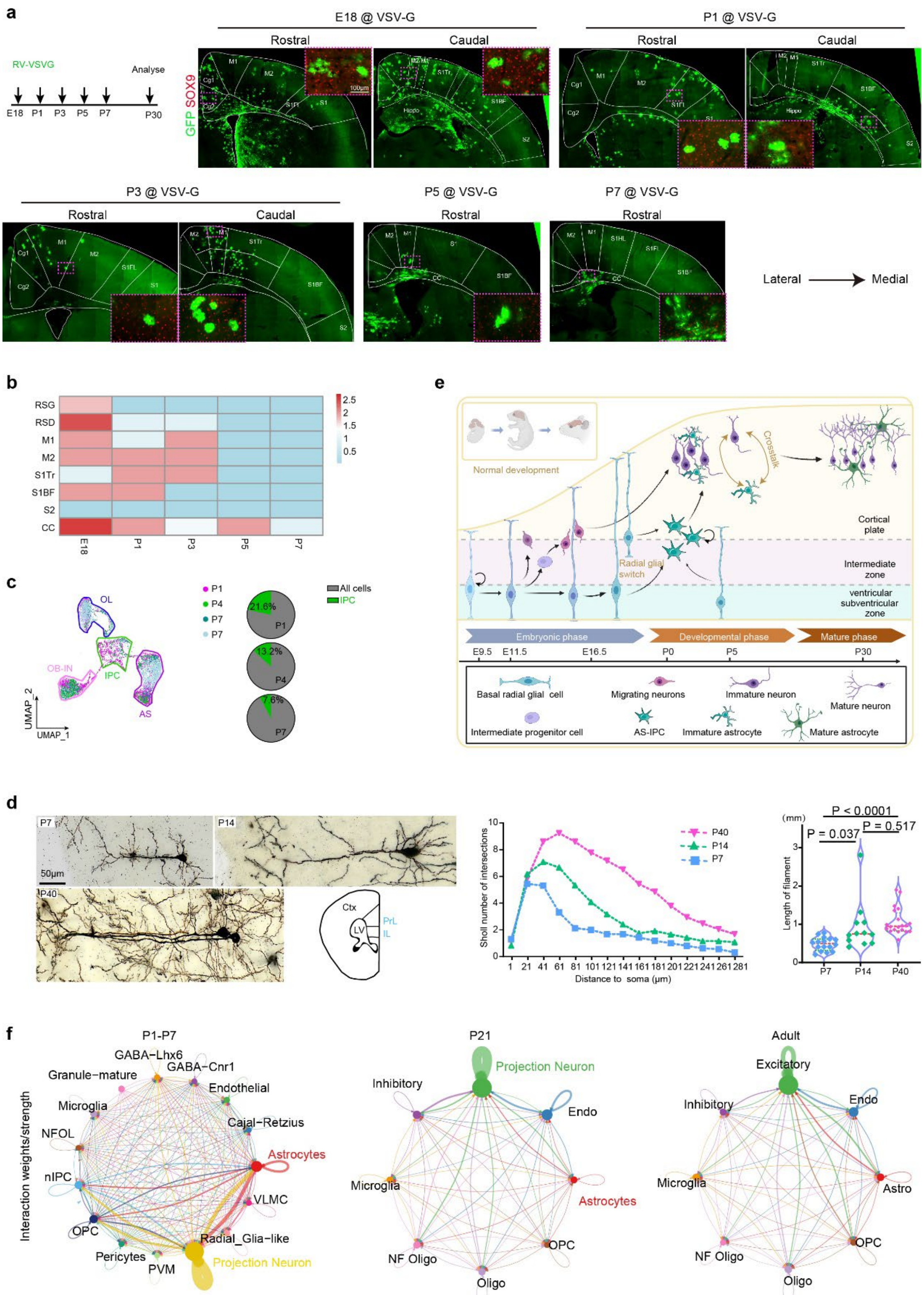

Supplementary Fig.1 | Astrocytes and neurons engage in close crosstalk during the first postnatal week. a. VSV-G retroviruses were injected in the lateral ventricles of mice from embryonic day 18 (E18) to postnatal day 7 (P7), and analyzed the labeled glia cells at P30. **b.** A heatmap shows the density of GFP-positive glia cells from different brain regions (n = 3 mice per group). **c.** UMAP visualization of the cortical cells (including astrocytes, olfactory bulb (OB) interneurons, oligodendrocytes and

intermediate progenitor cells) of P1, P4 and P7. **d.** Representative images of projection neurons of PFC usinng Golgi-Cox staining (left). Sholl analysis and length of filament of the projection neurons (right) (*n* = P7 (12 cells), P14 (16 cells), P40 (20 cells); mean ± SD; unpaired two-tailed Student's *t* test with Welch's correction). **e.** Summary of the developmental timeline for mouse cortical pyramidal neurons and astrocytes (Created in BioRender. Liu, G. (2026) https://BioRender.com/79dz396). Radial glial cells generate cortical astrocytes from E16 to P5, with immature astrocytes closely interacting with immature neurons during the first postnatal week. **f.** Circular plots depicting the interaction strength between each cell type in the cortex at P1-P7 (left), P21 (middle) and adult (right) stages. Detailed statistics are in Supplementary Data 5. Source data are provided as a Source Data file.

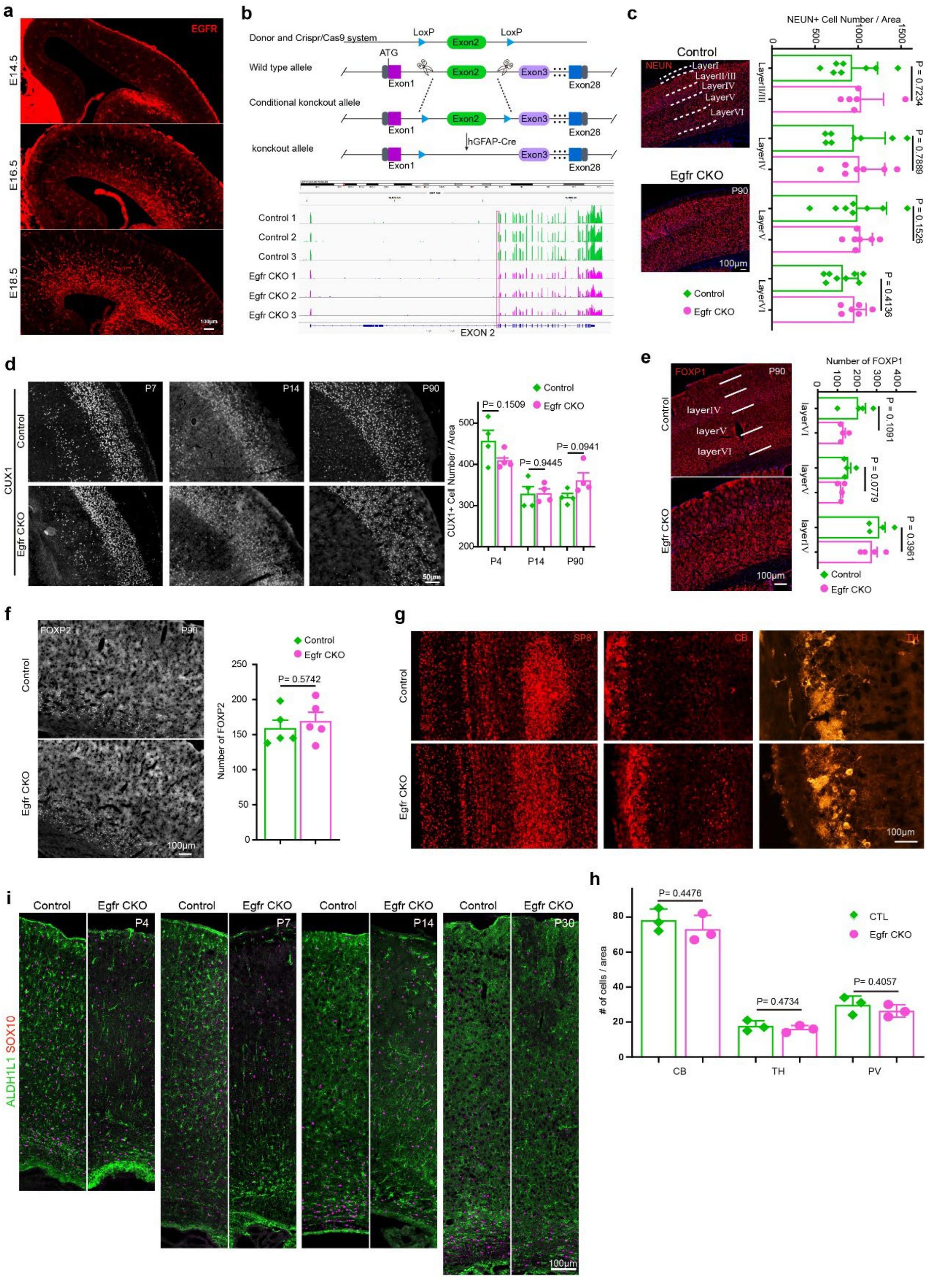

Supplementary Fig.2 | *Egfr* knockout impairs the development of astrocytes but not neurons. a. EGFR immunostaining at different embryonic stages. **b.** Schematic representation of the *Egfr* knockout allele and validation of RNA-seq data from the P1 cortex **c-f.** Representative images of NEUN, CUX1, FOXP1, and FOXP2 staining in the *Egfr* knockout cortex and control littermates, with quantification of cell numbers (*n* = 8 control mice and 7 *Egfr*-CKO mice (c); 4 mice per group (d-f); mean ± SD; unpaired two-tailed Student's *t* test). **g-h.** Immunostaining of OB interneuron markers and

quantification of the corresponding cell numbers (n = 3 mice per group; mean ± SD; unpaired two-tailed Student's t test). **i.** Expression of ALDH1L1 and SOX10 in *Egfr*-CKO mice and controls across P4 to P30. Detailed statistics are in Supplementary Data 5. Source data are provided as a Source Data file.

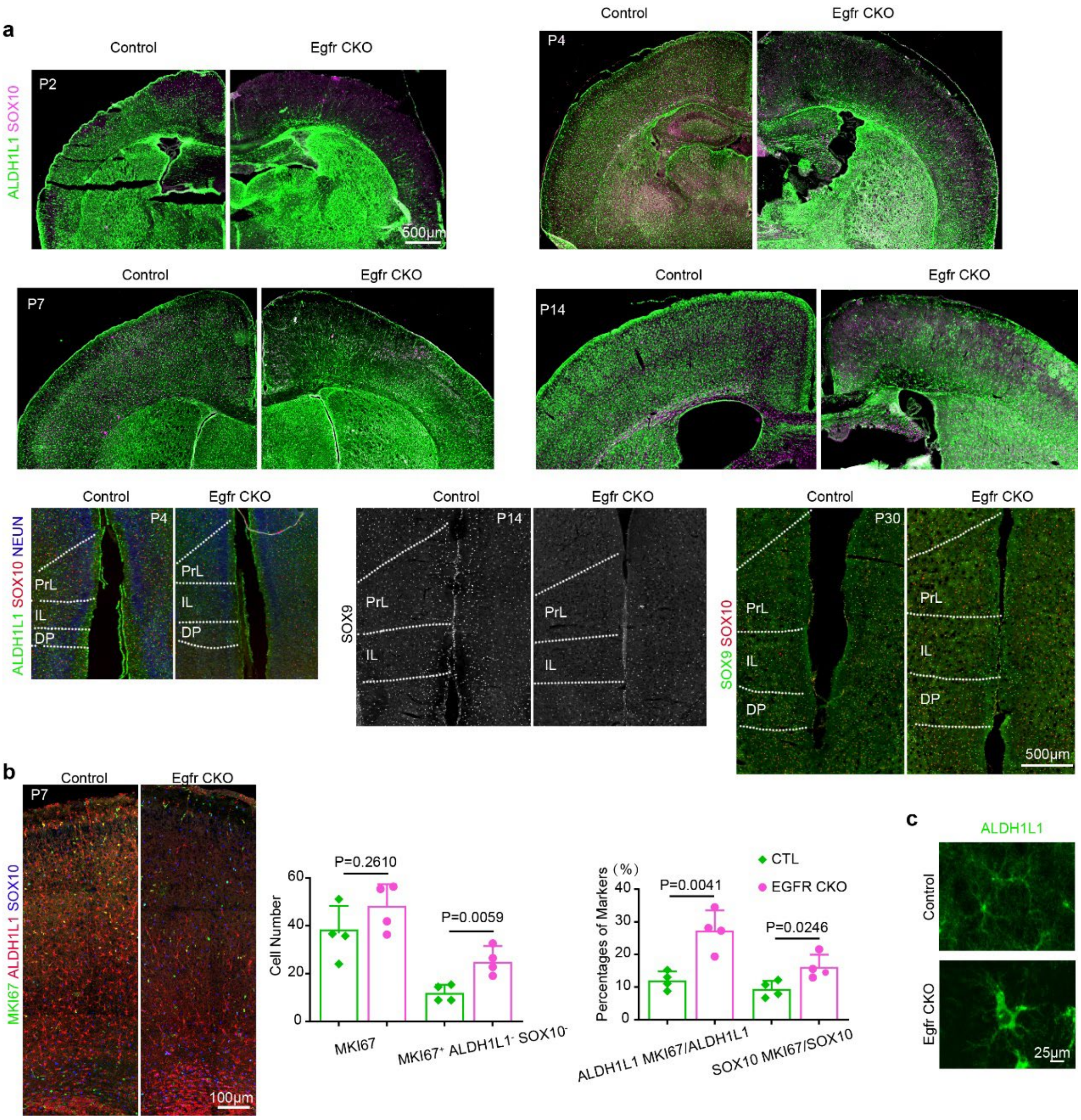

Supplementary Fig.3 | Astrocytes restoration through local proliferation. a. Expression of ALDH1L1 and SOX10 in *Egfr* knockout mice and controls across P2 to P14, showing astrocyte restoration. The bottom row shows astrocytes and oligodendrocytes in the mPFC region at P4, P14, and P30. **b.** Co-staining of ALDH1L1 and SOX10 with the proliferation marker KI67, with quantification of cell numbers and the ratio of proliferating astrocytes and oligodendrocytes (n = 4 mice per group; mean ± SD; unpaired two-tailed Student's t test). **c.** High-magnification images of ALDH1L1 in the cortex of *Egfr* knockout mice and their littermate controls, revealing significantly more active or altered morphology in the knockout group. Source data are provided as a Source Data file.

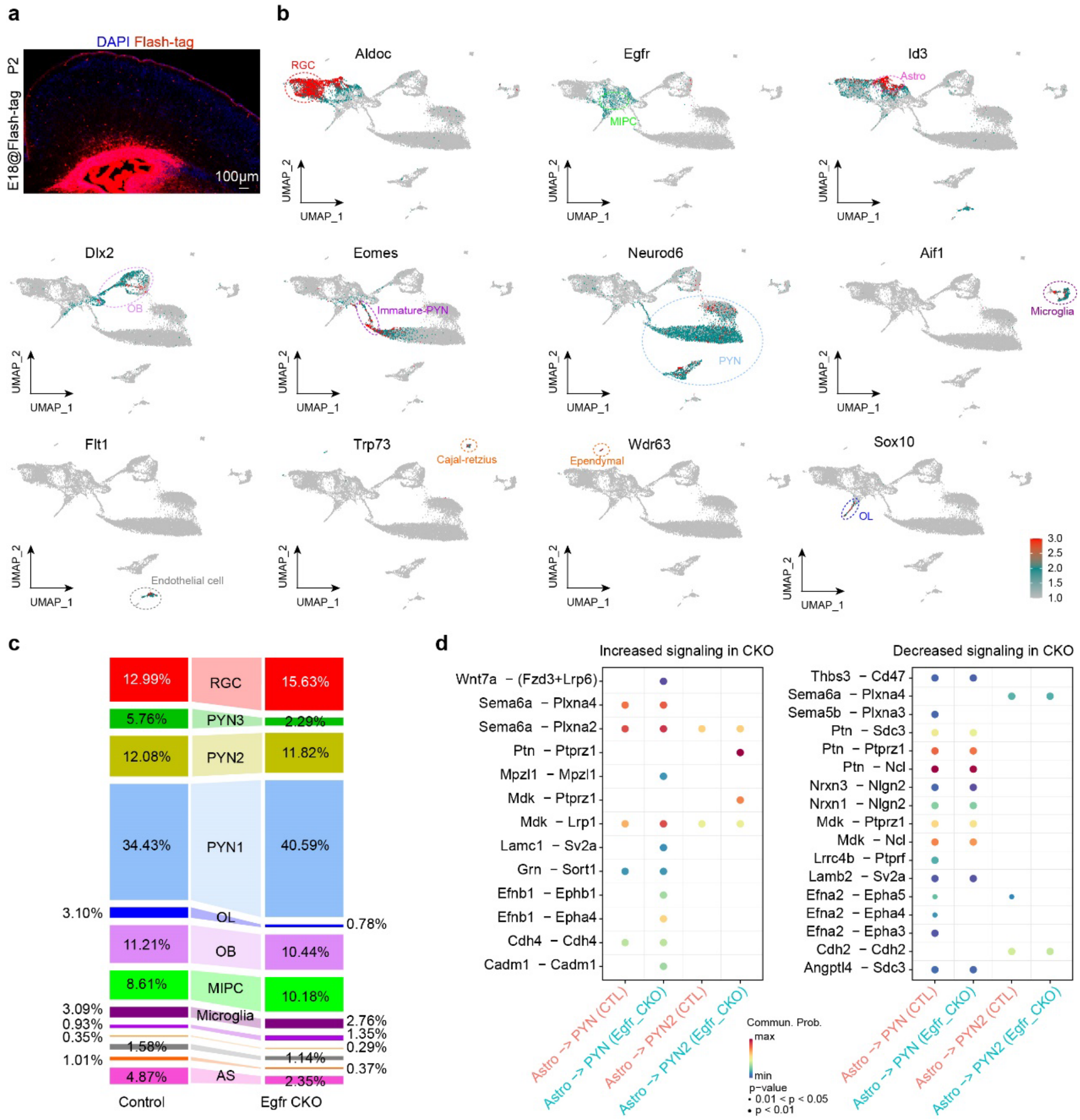

Supplementary Fig.4 | Single-cell RNA-seq data of *Egfr* knockout cortex and controls. a. Representative image showing labeled cells used for single-cell RNA-seq experiment. **b.** UMAP plot illustrating the expression of representative markers. **c.** Bar plot showing the percentages of number of each cluster. **d.** Dot plot showing altered signaling pathways in the *Egfr* CKO cortex. RGC: radial glia cells; MIPC: multipotent intermediate progenitor cells; OL: oligodendrocyte.

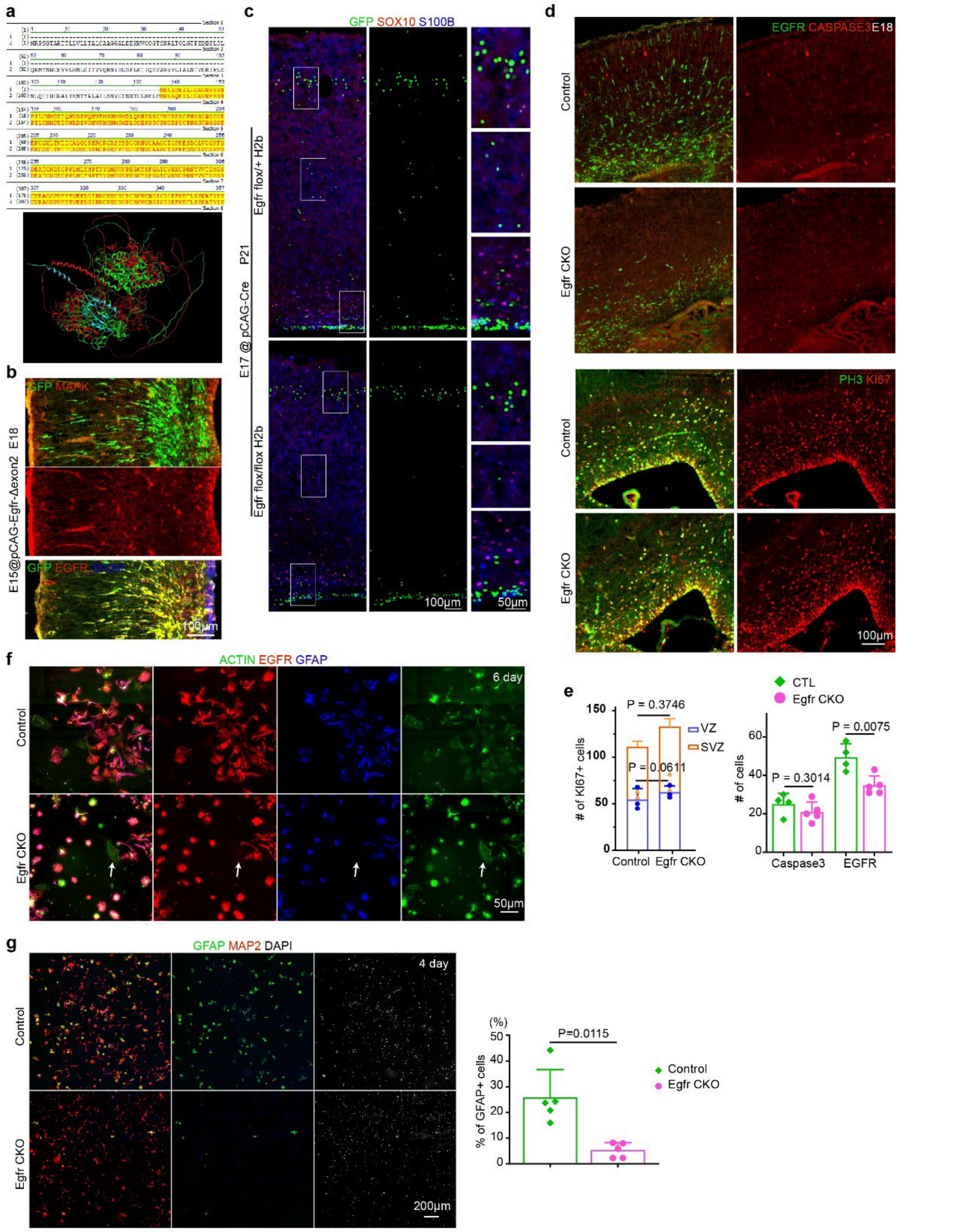

Supplementary Fig.5 | *Egfr* regulates the generation of astrocytes by modulating the migration of progenitor cells.

a. The truncated EGFR protein is initiated at AA137. The predicted truncated protein as determined by AlphaFold2, differs from the full-length protein. **b.** Plasmid expressing truncated EGFR proteins were electroporated into the mouse cortex at E15 and analyzed 3 days later. **c.** pCAG-Cre plasmids were electroporated into the ventricular zone of *Egfr* ^{flox} H2b mice at E17 and analyzed at P21. **d-e.** Representative images of CASPASE3, KI67 and PH3 staining for *Egfr* knockout mice and the control

mice at E18 (top); quantification of CASPASE3-, KI67-, and PH3-positive cells, respectively (n = 3 mice per group (KI67) 4 control mice and 5 *Egfr*-CKO mice (CASPASE3, EGFR); mean ± SD; unpaired two-tailed Student's t test). **f-g.** The neocortex of *Egfr* knockout mice and their littermate controls were dissected at P0 for primary cell culture in serum medium (**f**) and in serum-free medium (**g**) (n = 5; mean ± SD; unpaired two-tailed Student's t test with Welch's correction). Quantification data show that the number of astrocytes in knockout group was significantly reduced when cultured in serum-free medium. Detailed statistics are in Supplementary Data 5. Source data are provided as a Source Data file.

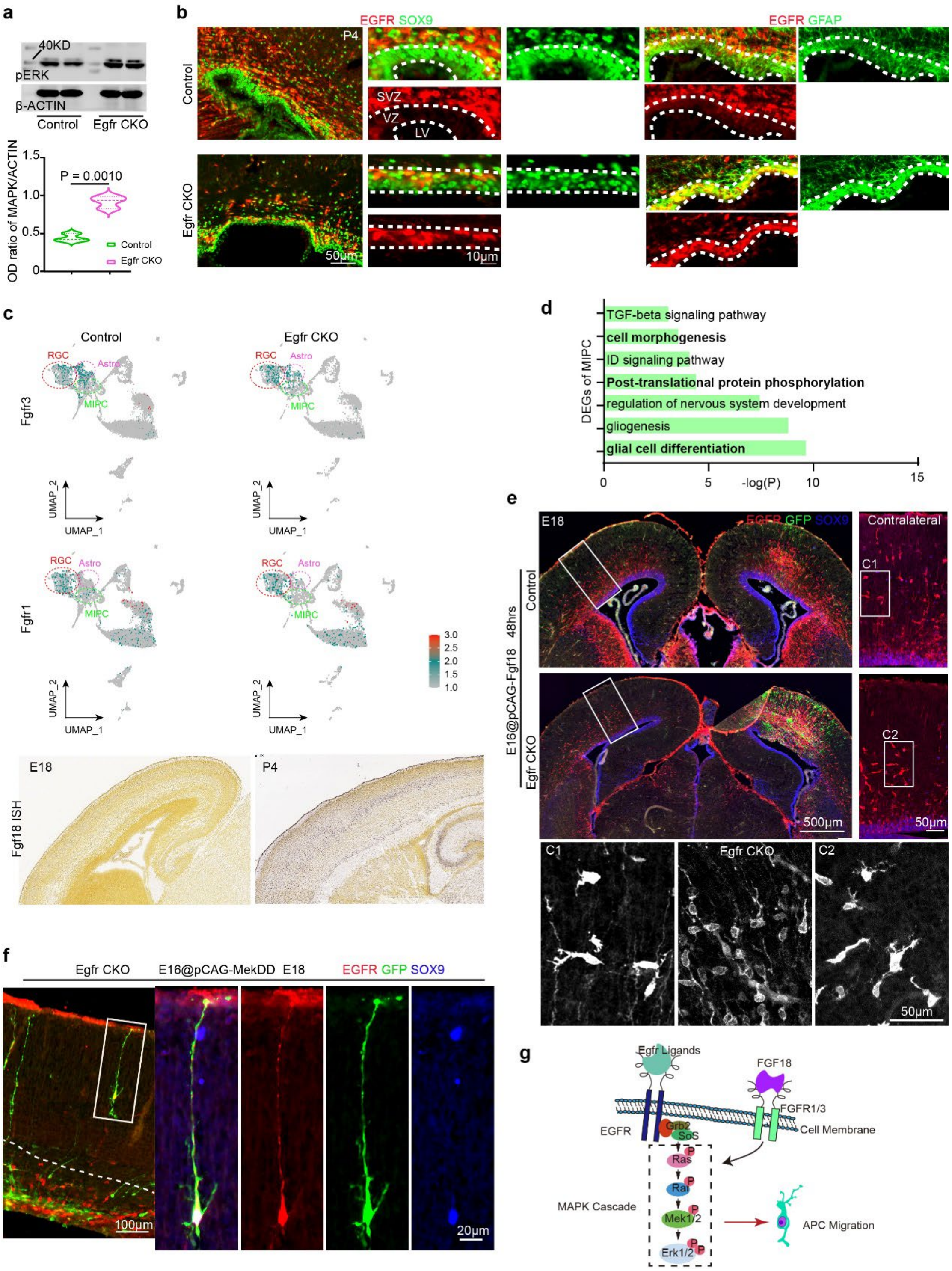

Supplementary Fig.6 | MAPK signaling promotes the migration of glia progenitor cells. a. Western blot analysis showing upregulation of pERK (MAPK) protein for the *Egfr* knockout cortex compared with the controls (n = 3 mice per group; mean ± SD; unpaired two-tailed Student's t test). **b.** Co-immunostaining of EGFR with SOX9 and GFAP in the cortex at P4. **c.** UMAP plot shows the expression of *Fgfr3* and *Fgfr1*. RNA in situ hybridization of *Fgf18* of mouse cortex at E18 and P4 (bottom, Allen brain). **d.** GO analysis of differentially expressed genes of MIPCs. **e-f.** pCAG-*Fgf18* or pCAG-*MekDD* plasmids were electroporated into the ventricular zone of *Egfr* knockout and

the littermate control mice at E16, and the mice were analyzed 2 days later. **g.** Diagram illustrating how the MAPK cascade promotes the migration of astrocyte precursor cells. Detailed statistics are in Supplementary Data 5. Source data are provided as a Source Data file.

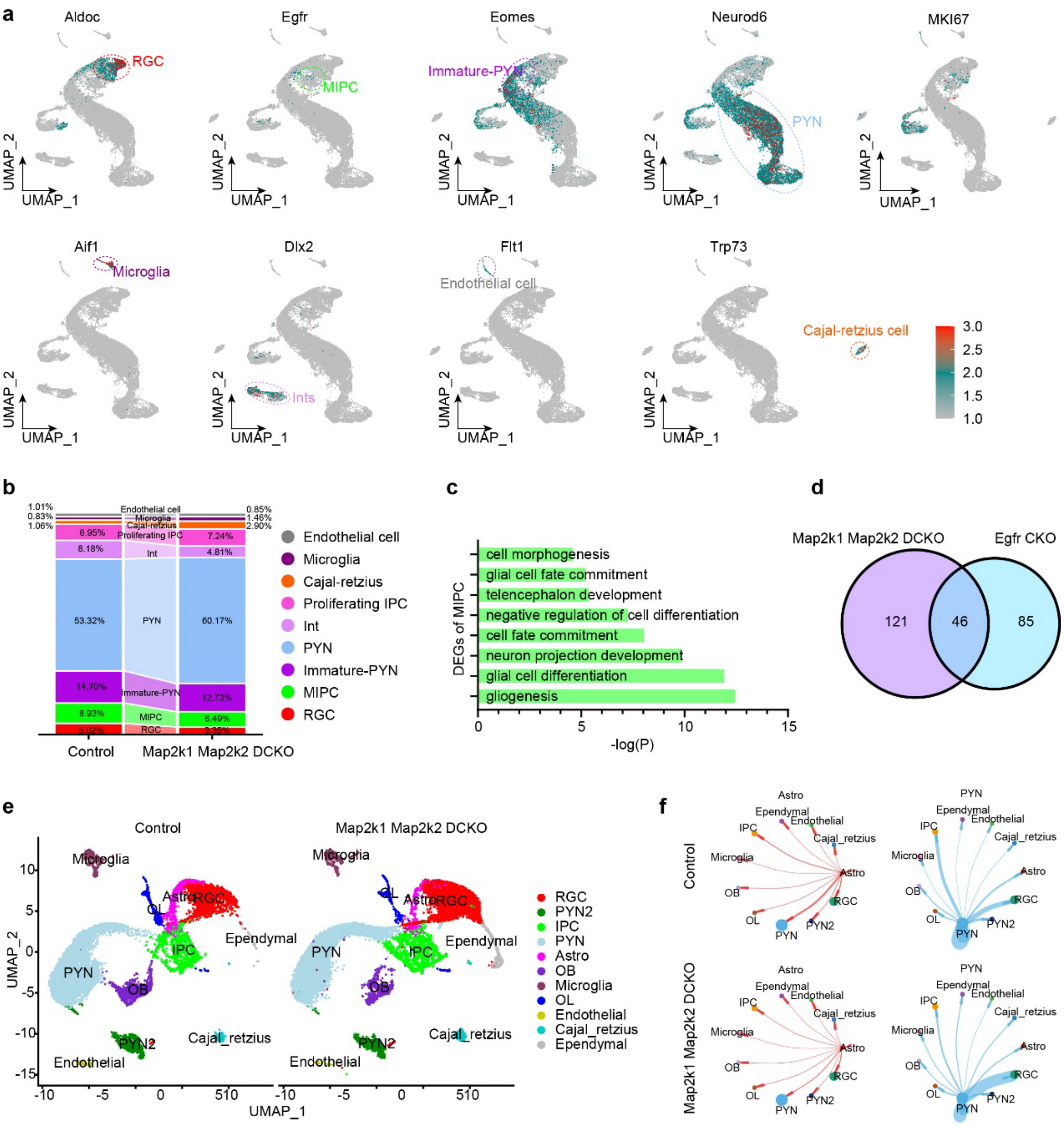

Supplementary Fig.7 | Single cell RNA-seq data of *Map2k1/2* double knockout cortex(hGFAP-Cre) and the controls. a. UMAP plot showing the expression of representative markers. **b.** Bar plot showing the percentages of number of each cluster. **c.** GO analysis of differentially expressed genes of multipotent MIPCs. **d.** Venn plot illustrates the number of differentially expressed genes of MIPCs in both *Map2k1/2* DCKO cortex and the *Egfr* knockout cortex. **e.** Flash-tag were injected into the ventricule of the *Map2k1/2* CKO mice at E18, and the labeled cells were sorted at P2 for single-cell RNA-sequencing. UMAP plot showing 11 cortical cell types. **f.** Cell chat analysis of the control cortex and the *Map2k1/2*-CKO cortex.

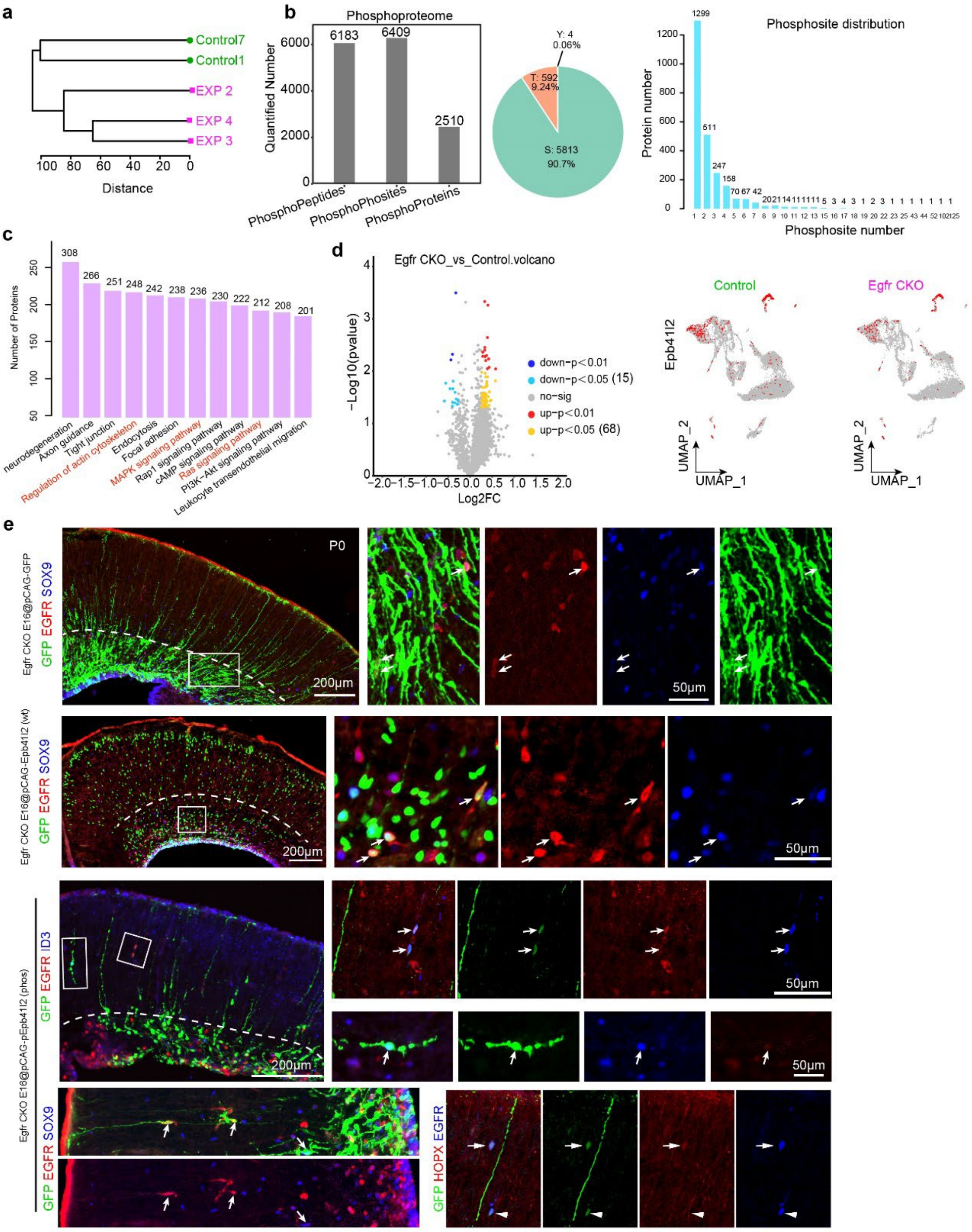

Supplementary Fig.8 | Phosphorylated EPB41L2 as a significant downstream target regulated by the EGFR-MAPK signaling pathway for glial migration. a. Hierarchical clustering of Liquid chromatography–mass spectrometry (LC-MS) data of the *Egfr* knockout cortex and the controls. **b.** Overview of LC-MS data. **c.** The top 12 KEGG pathways enriched for phosphorylated proteins. **d.** Volcano plot showing the differentially expressed proteins (left) and the UMAP plot displaying the expression of *Epb41l2* (right). **e.** pCAG-GFP, pCAG-*Epb41l2* (wt), or pCAG-*Epb41l2* (phosphorylated) plasmids were electroporated into the ventricle zone of *Egfr*-CKO mice at E16, and GFP-positive glia progenitor cells were analyzed at P0.

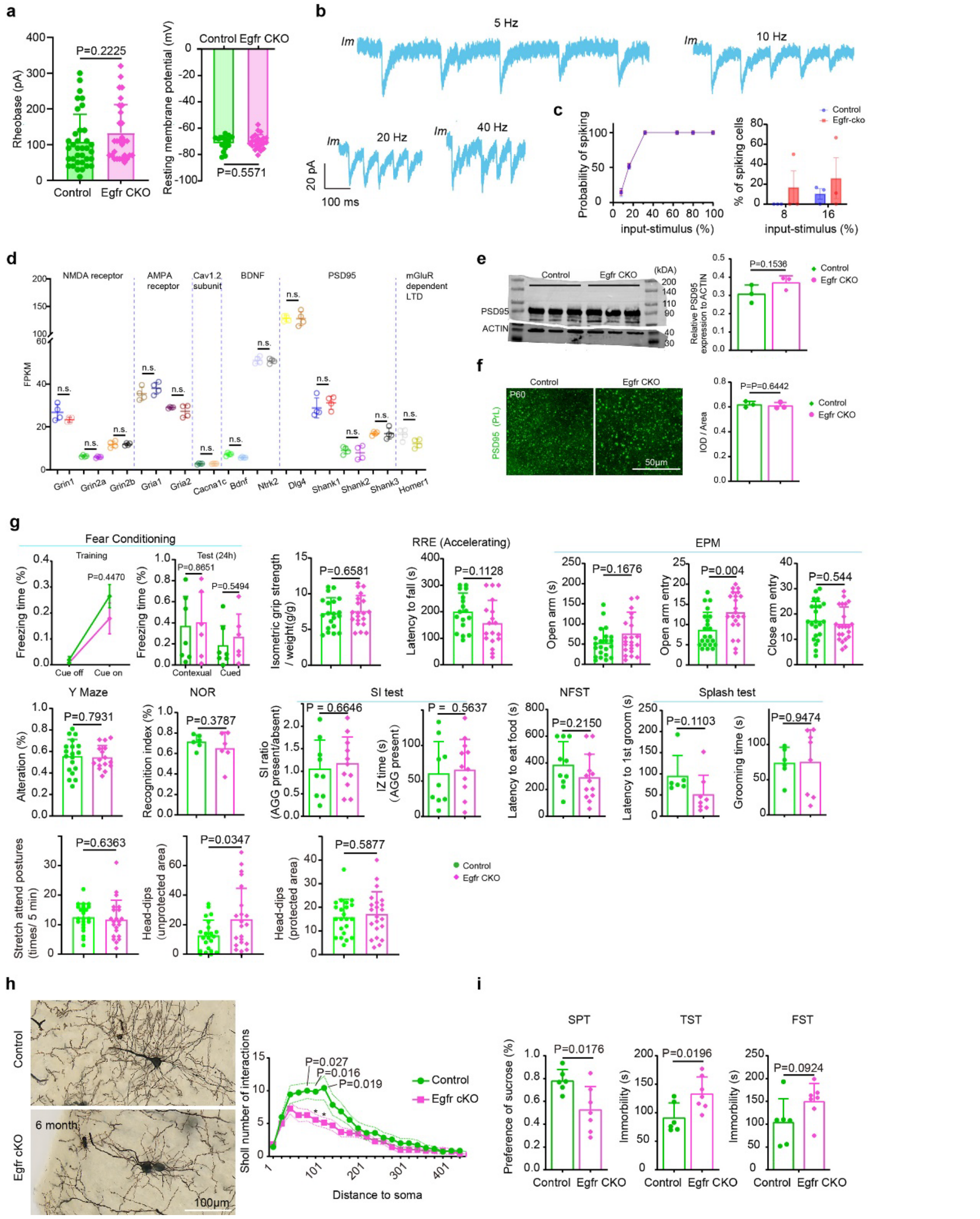

Supplementary Fig.9 | *Egfr* knockout impairs dendritic arborization and leads to depressive-like behaviors, but has no effect on dendritic spine morphology or plasticity. a. Electrophysiological properties of deep-layer pyramidal neurons in the PFC, including rheobase and resting membrane potential (n = 15 control cells and 23 *Egfr*-CKO cells (rest potential), n = 6 control mice with 35 cells vs. 5 *Egfr*-CKO mice with 29 cells (rheo base); mean±SD; unpaired two-tailed Student's t-test). **b.** Representative traces of synaptic dynamics in response to long-range input to the contralateral PFC across four stimulation frequencies (5, 10, 20, and 40 Hz). **c.** Left: Spike probability across a range of light intensities. Right: Percentage of activated cells in response to 8 Hz and 16 Hz stimulation (n = 3 mice per group, mean values or

mean±SD). **d.** RNA-seq analysis reveals no significant changes in the expression of synaptic plasticity-related genes in the PFC of *Egfr*-CKO mice (*n* = 4 mice per group, mean±SD; unpaired two-tailed Student's *t*-test). **e–f.** Western blot (**e**) and immunostaining (**f**) show unaltered PSD95 protein levels in the PFC of *Egfr* CKO mice (*n* = 3 mice per group, mean±SD; unpaired two-tailed Student's *t*-test). **g.** Behavioral performance of 2-month-old *Egfr*-CKO and control mice in the tests outlined in Figure **4g**. (Detailed statistics are in Supplementary Data 5.) **h.** Representative images of Golgi-stained prefrontal cortex and corresponding Sholl analysis from 6-month-old *Egfr* CKO and littermate control mice (*n* = 7 cells per group, mean values; unpaired two-tailed Student's *t*-test). **i.** Performance of 6-month-old *Egfr* CKO and control mice in depression-related behavioral assays (*n* = 6 control mice and 7 *Egfr*-CKO mice, mean±SD; unpaired two-tailed Student's *t*-test). Source data are provided as a Source Data file.

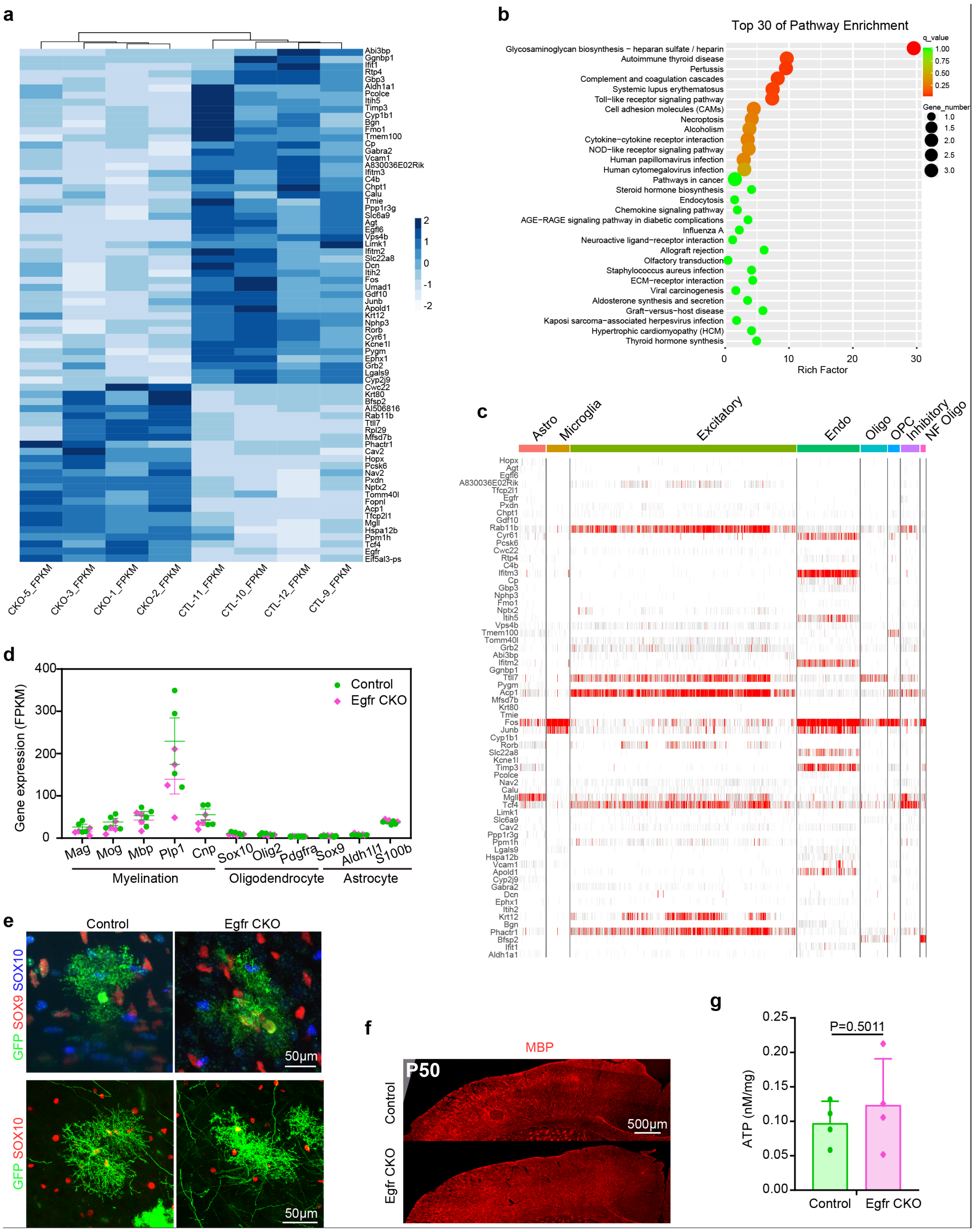

Supplementary Fig.10 | The properties of astrocytes in the PFC of *Egfr* knockout mice show no significant difference compared with those of control mice.

a. The PFC of *Egfr* knockout and their littermate controls were dissected for bulk RNA sequencing at P50. Heatmap showing the differentially expressed genes (DEGs) between the two groups. **b.** Pathway analysis of DEGs mentioned above**. c.** A heatmap illustrating the cell type specificity (PFC of adult mice) of all differentially expressed genes presented in figure **a**. **d.** Dot plot displaying the expression of genes associated with the myelination process, oligodendrocytes, and astrocytes. **e.** pCAG-GFP plasmid was electroporated into the cortex of *Egfr* CKO and control mice at P0 and analyzed at P21. Representative images showing the morphology of astrocytes and

oligodendrocytes. **f.** MBP stainning of the *Egfr* CKO cortex and the controls at P50. **g.** ATP measurement results showing ATP levels in the medium derived from PFC and of *Egfr* CKO mice, compared to those of the control group, at P60 (n= 4 mice per group, mean±SD; unpaired two-tailed Student's *t*-test). Detailed statistics are in Supplementary Data 5. Source data are provided as a Source Data file.

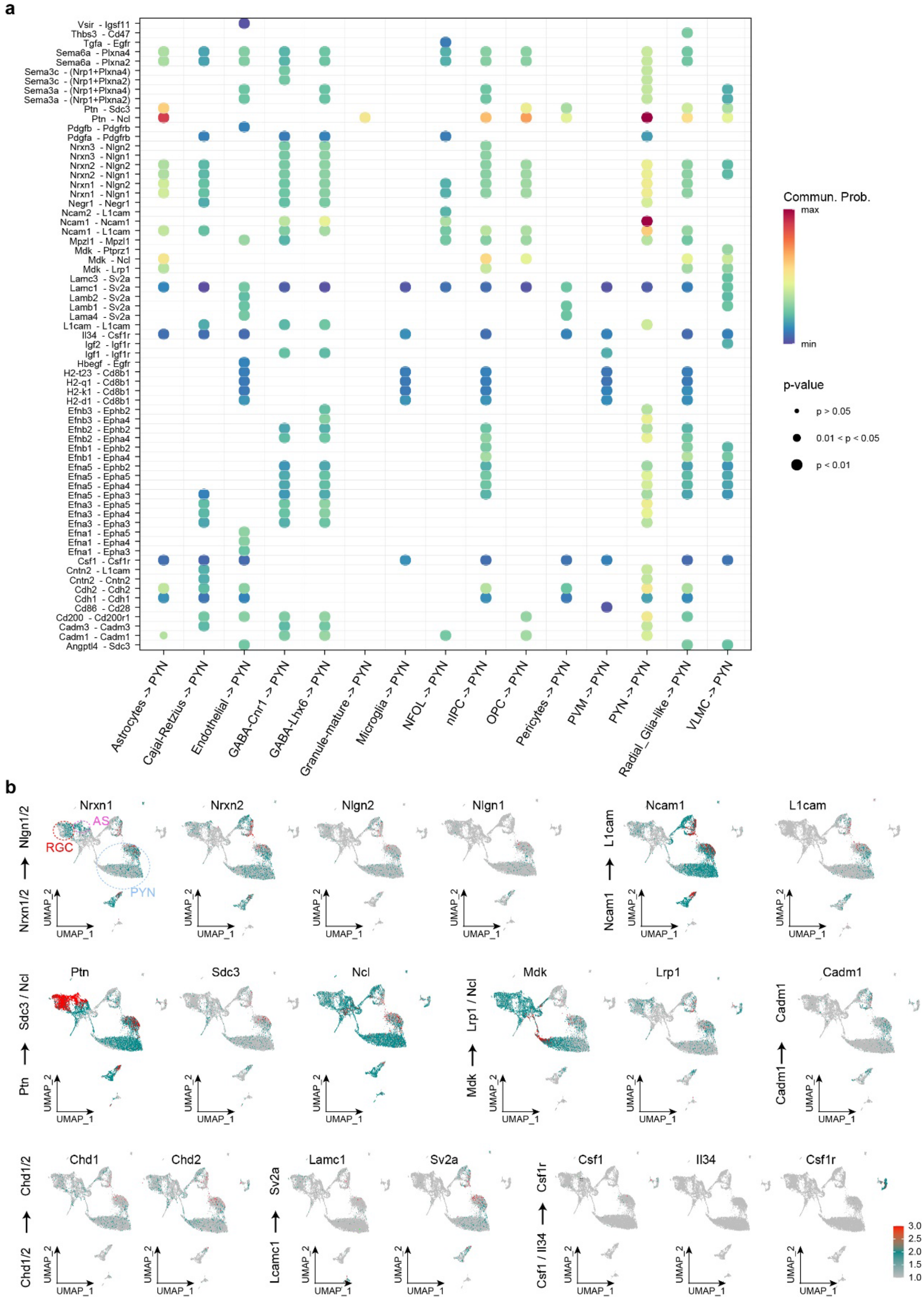

Supplementary Fig.11 | Neurons engage in close crosstalk with other cell types during the first postnatal week. a. Dot plot showing the ligand-receptor pairs related to neurons. **b.** UMAP plot depicting the expression of ligand-receptor pairs between astrocytes and neurons.

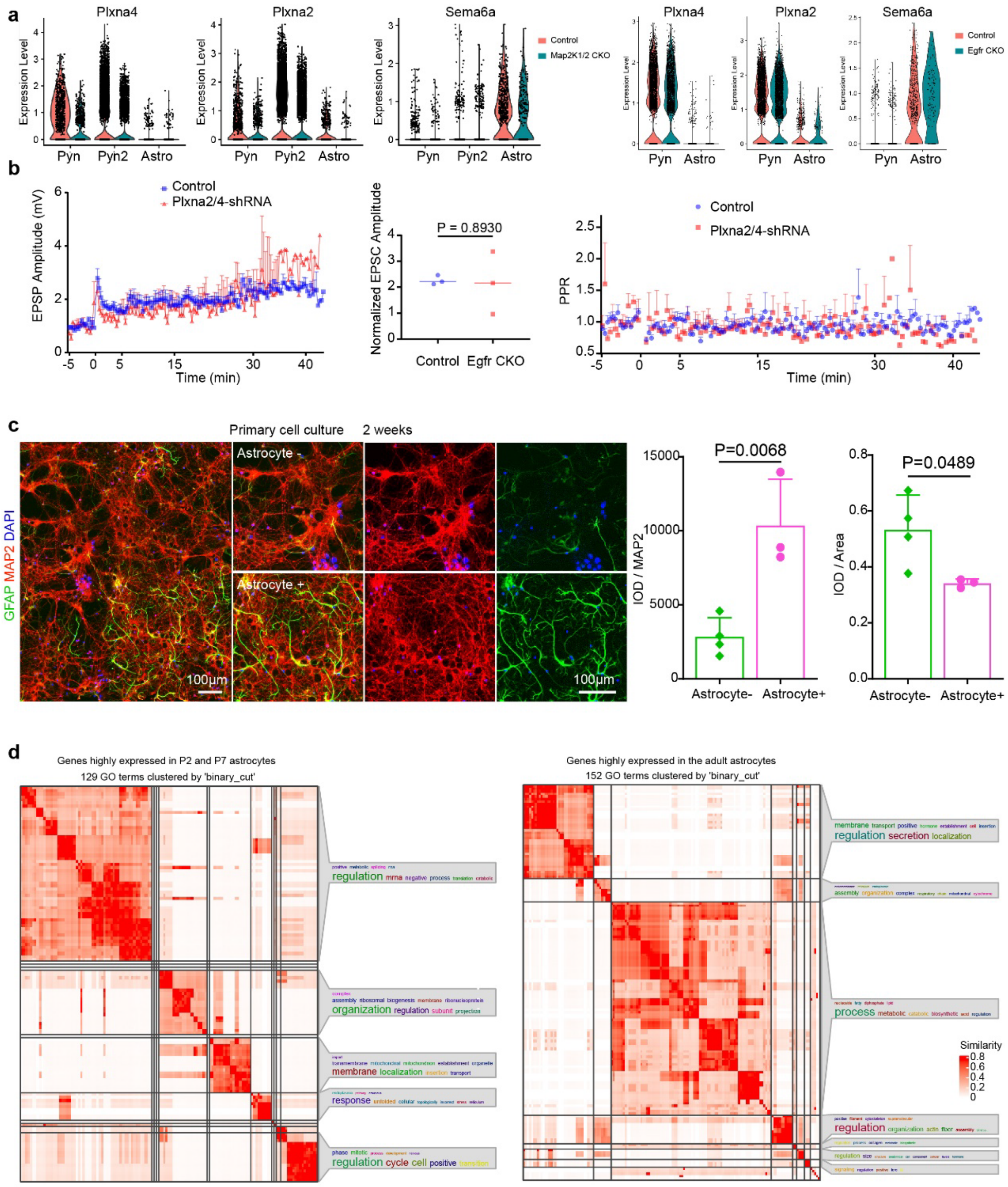

Supplementary Fig.12 | Direct contact between astrocytes and neurons Enhances neuronal complexity. a. The violin plot depicts the expression of *Plxna2/4* and *Sema6a* in projection neurons and astrocytes from both *Mapk1/2* DCKO and *Egfr* CKO cortex samples. **b.** To evaluate the role of *Plxna2/4*, we delivered *Plxna2/4*-shRNA or a control plasmid into the mouse medial prefrontal cortex (mPFC) by in utero electroporation at E13.5 and performed LTP recordings in adulthood (postnatal day 60, P60; n = 3 per group, mean±SD; unpaired two-tailed Student's t-test). The amplitude, normalized amplitude, and paired-pulse ratio (PPR) for the *Plxna2/4* knockdown and control mice are presented in the plot, respectively. **c.** The cortices of P0 mice were dissected for 2 weeks of primary cell culture. The MAP2 integral optical density of regions with or without astrocytes were estimated using ImageJ software (n = 4(Astrocyte^{-}), 3(Astrocyte^{+}); mean±SD; unpaired two-tailed Student's t-test). **d.** Genes highly

expressed in immature astrocytes and mature astrocytes were separately used for GO analysis by simplifyEnrichment package. Detailed statistics are in Supplementary Data 5. Source data are provided as a Source Data file.

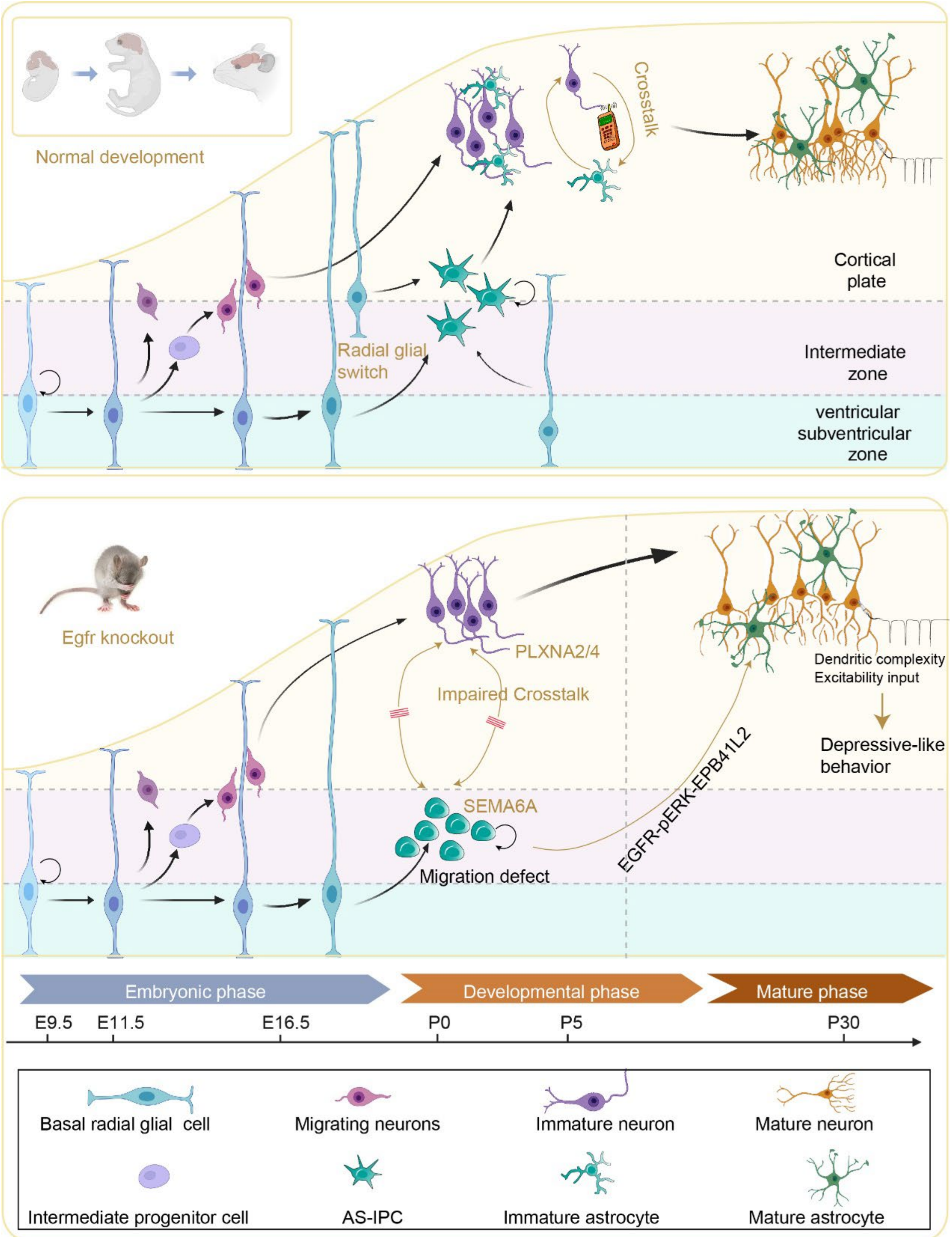

Supplementary Fig.13 | Graphical abstract. Delayed crosstalk between astrocytes and neurons reduces the complexity and excitability of neurons, leading to depressive-like behaviors. (Created in BioRender. Liu, G. (2026) https://BioRender.com/79dz396)

Source Data of blots

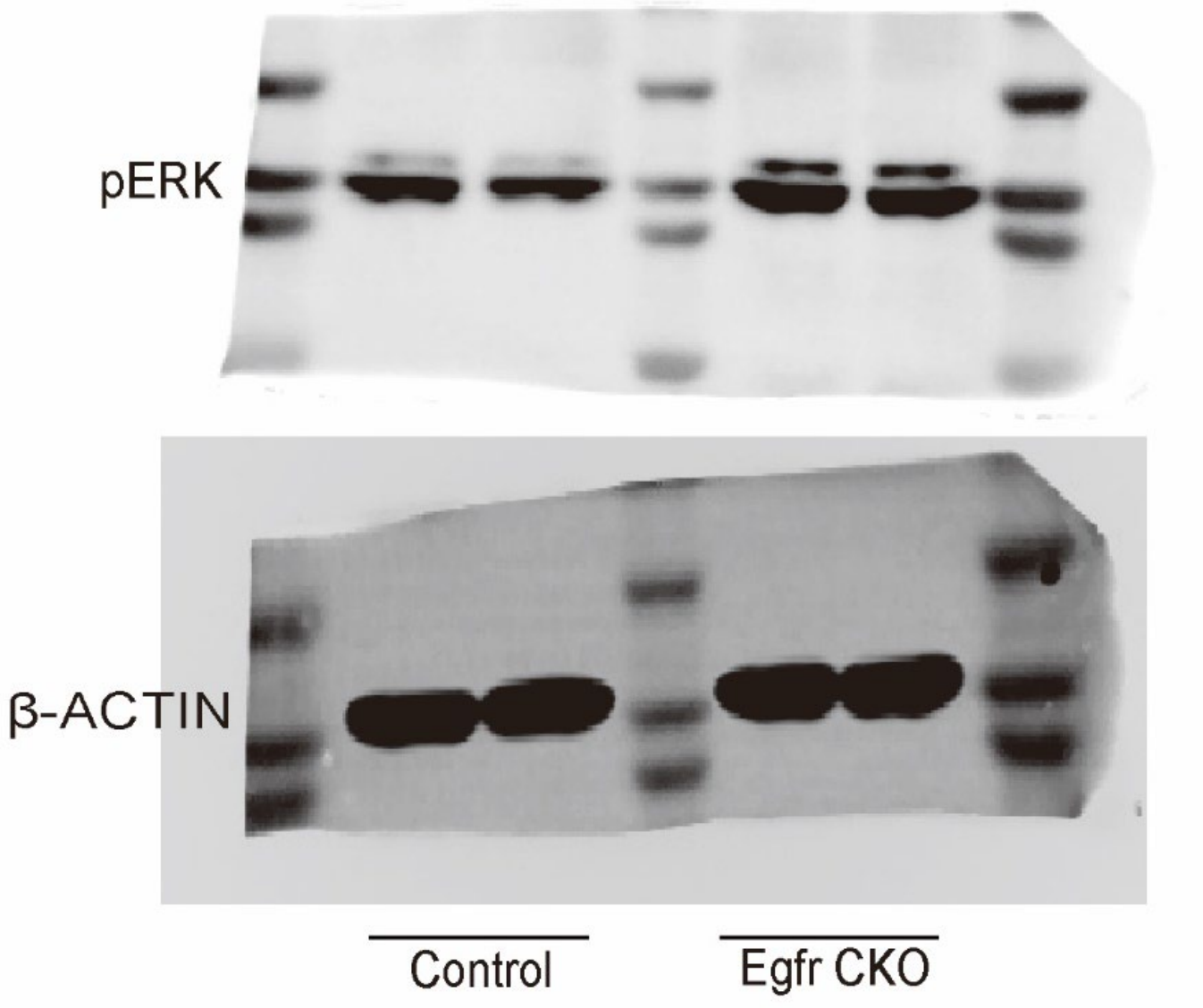

Uncropped raw blot for MAPK and β-ACTIN
