# Supplementary material for: Delayed astrocyte development impairs Sema6a-Plxna2/4-mediated astrocyte-neuron crosstalk and causes depressive-like behavior
Source: Nat Commun. 2026 Jul 18;17:8800. doi: 10.1038/s41467-026-75697-z (PMC13494039; doi:10.1038/s41467-026-75697-z)
Supplement: Supplementary file 3 [file 41467_2026_75697_MOESM2_ESM.pdf]

Description of Additional Supplementary Files

File name: Supplementary Data 1
Description: Cell communication analysis of P1, P4 and P7 mouse cortex.

File name: Supplementary Data 2
Description: Cell communication analysis of human prefrontal cortex from fetal to adult stages.

File name: Supplementary Data 3
Description: Cell communication analysis of the *Egfr* knockout cortex and the littermate controls at P2.

File name: Supplementary Data 4
Description: RNA sequencing of the prefrontal cortex in *Egfr* knockout mice and their littermate controls at P50.

File name: Supplementary Data 5
Description: This file contains detailed statistical information.

File name: Supplementary Data 6
Description: This file details the construction of all plasmids used in this study.
