# Supplementary material for: Delayed astrocyte development impairs Sema6a-Plxna2/4-mediated astrocyte-neuron crosstalk and causes depressive-like behavior
Source: Nat Commun. 2026 Jul 18;17:8800. doi: 10.1038/s41467-026-75697-z (PMC13494039; doi:10.1038/s41467-026-75697-z)
Supplement: Supplementary file 4 [file 41467_2026_75697_MOESM9_ESM.pdf]

nature portfolio

Corresponding author(s): Guoping Liu

Last updated by author(s): Jun 8, 2026

Reporting Summary

Nature Portfolio wishes to improve the reproducibility of the work that we publish. This form provides structure for consistency and transparency in reporting. For further information on Nature Portfolio policies, see our Editorial Policies and the Editorial Policy Checklist.

Statistics

For all statistical analyses, confirm that the following items are present in the figure legend, table legend, main text, or Methods section.

n/a	Confirmed	
☐	☒	The exact sample size (*n*) for each experimental group/condition, given as a discrete number and unit of measurement
☐	☒	A statement on whether measurements were taken from distinct samples or whether the same sample was measured repeatedly
☐	☒	The statistical test(s) used AND whether they are one- or two-sided<br>*Only common tests should be described solely by name; describe more complex techniques in the Methods section.*
☒	☐	A description of all covariates tested
☐	☒	A description of any assumptions or corrections, such as tests of normality and adjustment for multiple comparisons
☐	☒	A full description of the statistical parameters including central tendency (e.g. means) or other basic estimates (e.g. regression coefficient) AND variation (e.g. standard deviation) or associated estimates of uncertainty (e.g. confidence intervals)
☐	☒	For null hypothesis testing, the test statistic (e.g. *F*, *t*, *r*) with confidence intervals, effect sizes, degrees of freedom and *P* value noted<br>*Give P values as exact values whenever suitable.*
☒	☐	For Bayesian analysis, information on the choice of priors and Markov chain Monte Carlo settings
☒	☐	For hierarchical and complex designs, identification of the appropriate level for tests and full reporting of outcomes
☒	☐	Estimates of effect sizes (e.g. Cohen's *d*, Pearson's *r*), indicating how they were calculated

Our web collection on statistics for biologists contains articles on many of the points above.

Software and code

Policy information about availability of computer code

Data collection	no previously unreported software was used
Data analysis	no previously unreported software was used

For manuscripts utilizing custom algorithms or software that are central to the research but not yet described in published literature, software must be made available to editors and reviewers. We strongly encourage code deposition in a community repository (e.g. GitHub). See the Nature Portfolio guidelines for submitting code & software for further information.

Data

Policy information about availability of data

All manuscripts must include a data availability statement. This statement should provide the following information, where applicable:

- Accession codes, unique identifiers, or web links for publicly available datasets
- A description of any restrictions on data availability
- For clinical datasets or third party data, please ensure that the statement adheres to our policy

The Single-cell RNA-seq and bulk RNA-seq that support the findings of this study are available at Gene Expression Omnibus under accession number GSE282783 (https://www.ncbi.nlm.nih.gov/geo/query/acc.cgi?acc=GSE282783). Proteome data are available at iprox (PXD058068, https://www.iprox.cn//page/project.html?id=IPX0010286000). Human109 (fetal to adult) and mouse110,111 PFC Single-cell RNA-sequencing data (postnatal and adult) are from GEO dataset. No custom code was used. Source Data are provided with Source Data file. All other data are available from the corresponding authors upon request.

Research involving human participants, their data, or biological material

Policy information about studies with human participants or human data. See also policy information about sex, gender (identity/presentation), and sexual orientation and race, ethnicity and racism.

Reporting on sex and gender	not involving human participants
Reporting on race, ethnicity, or other socially relevant groupings	not involving human participants
Population characteristics	not involving human participants
Recruitment	not involving human participants
Ethics oversight	not involving human participants

Note that full information on the approval of the study protocol must also be provided in the manuscript.

Field-specific reporting

Please select the one below that is the best fit for your research. If you are not sure, read the appropriate sections before making your selection.

☒ Life sciences ☐ Behavioural & social sciences ☐ Ecological, evolutionary & environmental sciences

For a reference copy of the document with all sections, see nature.com/documents/nr-reporting-summary-flat.pdf

Life sciences study design

All studies must disclose on these points even when the disclosure is negative.

Sample size	no sample size calculation was performed.
Data exclusions	no data were excluded
Replication	all experiments were replicated at least 3 times.
Randomization	not relevant
Blinding	The experimenters were blinded to the group assignments during the behavioral tests.

Reporting for specific materials, systems and methods

We require information from authors about some types of materials, experimental systems and methods used in many studies. Here, indicate whether each material, system or method listed is relevant to your study. If you are not sure if a list item applies to your research, read the appropriate section before selecting a response.

Materials & experimental systems

n/a	Involved in the study	
☐	☒	Antibodies
☒	☐	Eukaryotic cell lines
☒	☐	Palaeontology and archaeology
☐	☒	Animals and other organisms
☒	☐	Clinical data
☒	☐	Dual use research of concern
☒	☐	Plants

Methods

n/a	Involved in the study	
☒	☐	ChIP-seq
☒	☐	Flow cytometry
☒	☐	MRI-based neuroimaging

Antibodies

Antibodies used	GFP (Chicken, Aves Labs GFP-1020), tdTomato (Goat, SICGEN Ab8181), ASCL1 (Rabbit, Cosmo Bio SKT01-003), SP8 (Goat, Santa Cruz Biotechnology Sc-104661), KI67 (Mouse, BD Pharmingen 556003), OLIG2 (Rabbit, OasisBiofarm OB-PRB066), ALDH1L1 (Rabbit, Abcam, ab10712968), EGFR (Goat, R&D Systems, AB-355937), GFAP(Rat, Invitrogen, 13-0300), SOX10 (Guinea pig, R&D Systems, AF2864), SOX9 (Rabbit, Abcam, ab185966). HOPX (Rabbit, Proteintech, 11419-1-AP), PDGFRA (Rat, BD Pharmingen, 558774), ID3 (Rabbit, Biocheck, BCH-4/17-3), S100B (Rabbit, DAKO, Z0311), GS (Rat, oasis biofarm, OB-PRT016), NEUN (Rabbit, Abcam, ab177487), CUX1 (Rabbit, Santa Cruz Biotechnology Sc-13024), MAP2 (Rabbit, Proteintech, 17490-1-AP), beta-ACTIN (Mouse, abclonal, AC004), CB (Rabbit, Swant), FOXP1(Rabbit, Abcam, ab314488), PH3 (Rabbit, Millipore 06-570), FOXP2 (Goat, Santa Cruz, sc-21069), Cleave Caspase-3 (Rabbit, Cell Signaling, 9661), Tyrosine Hydroxylase (TH; mouse, sc-25269), PSD95(Rabbit, Abcam, 238135) .
Validation	GFP, https://cdn.shopify.com/s/files/1/0512/5793/4009/files/GFP_datasheet.pdf; tdTomato,https://www.origene.com/catalog/antibodies/tag-antibodies/ab8181-200-tdtomato-goat-polyclonal-antibody; ASCL1, https://www.cosmobiousa.com/products/anti-ash-1-pab;SP8 Rabbit Anti-SP8:https://datasheets.scbt.com/sc-104661.pdf; KI67:https://www.bdbiosciences.com/zh-cn/products/reagents/flow-cytometry-reagents/research-reagents/single-color-antibodies-ruo/purified-mouse-anti-ki-67.550609?tab=antibody_details; ;Rabbit Anti-OLIG2:https://www.oasisbiofarm.net/oasisFiles/20250713/1752374286417.pdf; ALDH1L1 ,https://www.abcam.cn/products/primary-antibodies/aldh1l1-antibody-astrocyte-marker-ab87117, EGFR, https://www.mndsystems.com/cn/products/human-egfr-antibody af231, GFAP,https://www.thermofisher.cn/cn/zh/antibody/product/GFAP-Antibody-clone-2-2B10-Monoclonal/13-0300, SOX10, https://www.oasisbiofarm.cn/#/productdetails?prold-aa03f9aeb86f45e6bf6e05b1d6ab60cf&exp2; SOX9,https://www.abcam.com/en-us/products/primary-antibodies/sox9-antibody-epr14335-78-ab185966; HOPX, https://www.ptgcn.com/products/HOPX-Antibody-11419-1-AP.htm, PDGFRA,https://www.bdbiosciences.com/zh-cn/products/reagents/western-blotting-and-molecular-reagents/western-blot-reagents/purified-rat-anti-mouse-cd140a.558774?tab=product_details; ID3, https://www.creativebiolabs.net/anti-id3-recombinant-antibody-clone-bch-4-17-3-114146.htm, S100B:https://www.labome.com/product/Dako/Z0311.html; GS,https://m.ny-bio.com/pd.jsp?mid=3&pid=166051&sortName=name&groupId=475&desc=true; NEUN,https://www.abcam.com/en-us/products/primary-antibodies/neun-antibody-epr12763-neuronal-marker-ab177487, CUX1,https://www.citeab.com/antibodies/809835-sc-13024-cdp-antibody-m-222; MAP2,https://www.ptgcn.com/Products/MAP2B-Antibody-17490-1-AP.htm; beta-ACTIN,https://abclonal.com.cn/catalog/AC004; CB,https://shop.swant.com/cb38a-calbindin-1-ml/CB38a-1-mL; FOXP1, https://www.abcam.cn/products/primary-antibodies/foxp1-antibody-epr26484-79-ab314488; PH3,https://www.sigmaaldrich.cn/CN/zh/product/mm/06570; FOXP2,https://www.scbt.com/p/foxp2-antibody-n-16; Cleave Caspase-3,https://www.cellsignal.com/products/primary-antibodies/cleaved-caspase-3-asp175-antibody/9661; Tyrosine Hydroxylase,https://www.scbt.com/p/th-antibody-f-11; PSD95,https://www.abcam.com/en-us/products/primary-antibodies/psd95-antibody-epr23124-118-synaptic-marker-ab238135.

Animals and other research organisms

Policy information about studies involving animals; ARRIVE guidelines recommended for reporting animal research, and Sex and Gender in Research

Laboratory animals	The background strains of transgenic mice used in this study included C57BL/6J and ICR (CD1). CD1, hGFAP-Cre, Rosa-YFP2 flox, Rosa-H2B-GFP flox mice were described previously. Map2k1 (exon 2) flox, Map2k2 (exon 4-9) flox, Egfr (exon 2) flox were constructed by GemPharmatech, Nanjing, China.
Wild animals	No wild animals were used in the study.
Reporting on sex	Males and females equally utilized throughout all experiments
Field-collected samples	No field-collected samples were used in the study
Ethics oversight	All experiments involving mice were performed in accordance with the Fudan University, Shanghai Medical College Laboratory Animal Research Center guidelines.

Note that full information on the approval of the study protocol must also be provided in the manuscript.

Plants

Seed stocks	No plants were used in the study.
Novel plant genotypes	No plants were used in the study.
Authentication	No plants were used in the study.
